# Bisphenol B Exposure Induces Miscarriage by Suppressing Migration/Invasion and Migrasome Formation

**DOI:** 10.1002/advs.202504871

**Published:** 2025-11-21

**Authors:** Wenxin Huang, Manli Wang, Yi Sun, Xiaoping Yue, Xun Li, Yanbing Lin, Geng Guo, Depeng Zhao, Qigang Fan, Xiaoling Ma, Zhihong Zhang, Shuaishuai Xing, Xiaoting Shen, Huidong Zhang

**Affiliations:** ^1^ Research Center for Environment and Female Reproductive Health The Eighth Affiliated Hospital Sun Yat‐sen University Shenzhen 518033 China; ^2^ NHC Key Laboratory of Male Reproduction and Genetics Guangdong Provincial Reproductive Science Institute (Guangdong Provincial Fertility Hospital) Guangzhou 510600 China; ^3^ Key Laboratory of Pharmaceutical Quality Control of Hebei Province College of Pharmaceutical Sciences Hebei University Baoding 071002 China; ^4^ Department of General Surgery The First Hospital of Lanzhou University Lanzhou 730000 China; ^5^ Department of Emergency Cerebrovascular Disease Center First Hospital of Shanxi Medical University Taiyuan 030001 China; ^6^ Department of Reproductive Medicine Shenzhen Maternity and Child Healthcare Hospital Women and Children's Medical Center Southern Medical University Shenzhen Guangdong Province 518033 China; ^7^ Department of Reproductive Medicine The First Hospital of Lanzhou University The First Clinical Medical School of Lanzhou University Lanzhou 730000 China; ^8^ MOE Key Laboratory of Coal Environmental Pathogenicity and Prevention Shanxi Medical University Taiyuan 030001 China

**Keywords:** environmental BPB (bisphenol B), female trophoblast cell migration/invasion, migrasome formation, non‐coding RNA lnc‐HZ04, PKCA/RAC1/CXCL12, unexplained miscarriage

## Abstract

Unexplained miscarriage (UM) remains challenge due to unclear pathogenesis and biological mechanisms. BPB (Bisphenol B), an extensively used endocrine disrupting chemical, has been widely detected out in human. Migrasomes are newly identified cellular organelles with a large number of unknown functions. However, whether and how BPB exposure may suppress migrasome formation (MF) to induce miscarriage are completely unknown. In this study, it is found that higher urinary BPB levels are associated with the suppressed MF in villous tissues and unexplained miscarriage. It is further confirmed that BPB exposure suppresses MF in the mouse placenta and thus induces miscarriage. Supplement with Pkca or Tspan4, two essential proteins for migration/invasion (MI) and MF, can efficiently treat against BPB‐induced miscarriage. In biological mechanisms, BPB up‐regulates ER levels, enhances its interactions with the lnc‐HZ04 promoter region, and thus promotes ER‐mediated lnc‐HZ04 transcription. Subsequently, lnc‐HZ04 suppresses TCF4‐mediated PKCA transcription and subsequently suppresses MI and MF. Collectively, this study not only identifies BPB as a novel risk factor for unexplained miscarriage, discovers novel pathogenesis and biological mechanisms in BPB‐induced miscarriage, but also provides potential targets for treatment against unexplained miscarriage.

## Graphical abstract

BPB (Bisphenol B) exposure up‐regulates ER (estrogen receptor) levels, enhances its interactions with the lnc‐HZ04 promoter region, and thus promotes ER‐mediated lnc‐HZ04 transcription. Subsequently, lnc‐HZ04 suppresses TCF4 (transcription factor 4)‐mediated PKCA (protein kinase C alpha) transcription and subsequently suppresses migration/invasion and migrasome formation.

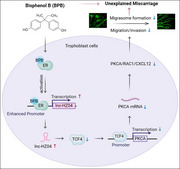

## 1 Introduction

First‐trimester miscarriage (or early pregnancy loss) is one of the most common complications in pregnancy.^[^ ^1^, ^2^ ^]^ Approximately 15–25% pregnant women experience spontaneous miscarriage, and 1–5% suffer from recurrent miscarriage.^[^ ^3^ ^]^ Many factors might induce miscarriage, including chromosomal abnormalities, uterine deformation, hormonal abnormalities, infections, psychological trauma and stressful life events, and immune disorders.^[^ ^4^ ^]^ However, there are still 50% causes unidentified,^[^ ^5^ ^]^ which are generally termed as unexplained miscarriage (UM). Increasing evidences have indicated that exposure to environmental toxicants is highly associated with UM.^[^ ^6^, ^7^, ^8^ ^]^ However, the underlying mechanisms are still largely unexplored.

Endocrine disrupting chemicals (EDCs) can interfere with hormone action and disrupt endocrine functions that harm human and animal health.^[^ ^9^ ^]^ Bisphenol A (BPA), which has been widely used in industrial production, is one of the most abundant EDCs^[^ ^10^ ^]^ and has shown various adverse effects on reproductive systems.^[^ ^11^, ^12^, ^13^, ^14^, ^15^, ^16^ ^]^ Due to the toxicity of BPA (Bisphenol A),^[^ ^17^ ^]^ BPB (Bisphenol B) is emerged as a substitute of BPA and has been widely used in the production of polycarbonate plastics and resins.^[^ ^18^ ^]^ BPB has been widely detected out from various foods, such as canned foods, drinks, meat, peeled tomatoes, beverages, and milk for infants.^[^ ^19^, ^20^, ^21^ ^]^ Moreover, BPB is also more prone to bio‐accumulation because it is resistant to aerobic and anaerobic bio‐degradation.^[^ ^10^ ^]^ It has been reported that BPB are detected from human serum samples (median 0.906 ng mL^−1^ and max 2.52 ng mL^−1^) in Shandong Province (China) and from urine samples (median 0.033 ng mL^−1^ and max 0.142 ng mL^−1^) in Guangdong Province (China).^[^ ^22^, ^23^ ^]^ Animal model experiments also show that BPB exposure suppresses meiotic maturation and damages oocyte quality by altering a lot of events, including spindle assembly and chromosome alignment, acetylation of α‐tubulin, DNA damage, epigenetic modifications, and ER (estrogen receptor) dynamics in mouse oocytes.^[^ ^20^ ^]^ However, whether and how BPB exposure might induce miscarriage is completely unknown and should be urgently explored.

Trophoblast cells play crucial roles in a healthy pregnancy. They invade the uterine spiral artery for vascular remodeling, invade the uterine decidual to promote embryo implant, and secrete hormones to regulate embryonic development.^[^ ^24^ ^]^ Dysfunctions of trophoblast cells are always related with female reproductive diseases, such as miscarriage.^[^ ^25^ ^]^ It has been reported that inhibition of human trophoblast cell migration/invasion may induce miscarriage.^[^ ^26^, ^27^, ^28^ ^]^ Notably, trophoblast cells are very sensitive to environmental toxicants. It has been reported that phthalates, parabens, polychlorinated biphenyls, or organophosphate might induce trophoblast cell dysfunctions.^[^ ^29^ ^]^ In our recent studies, we have found that exposure to BPDE (benzo(a)pyrene‐7,8‐dihydrodiol‐9,10‐epoxide) increases the apoptosis levels and suppresses proliferation, invasion, and migration in human trophoblast cells.^[^ ^28^, ^30^ ^]^ However, whether BPB exposure might suppress trophoblast migration/invasion to induce miscarriage is completely unknown and should be explored.

Migrasome is a newly identified cellular organelle with vesicle‐like morphology.^[^ ^31^, ^32^, ^33^ ^]^ During cell migration, fibers are protruded from the rear of cells, and migrasomes grow on these fibers. When cells migrate away, the fibers break, and migrasomes are left behind.^[^ ^34^ ^]^ Cellular components such as cytosol can be released from cells through migrasomes. It has been reported that migrasomes govern critical cellular processes including mitochondrial quality control,^[^ ^35^ ^]^ cell–cell communications,^[^ ^36^ ^]^ and lateral transfer of mRNAs and proteins.^[^ ^37^ ^]^ Meanwhile, migrasome formation strictly depends on cell migration. Blocking migration using Myosin II inhibitor or modulating cell adhesions inhibits migrasome formation in MGC803 cells.^[^ ^34^, ^35^ ^]^ However, whether migrasomes might form during human trophoblast cells migrate and whether BPB exposure might affect this process are largely unknown. Moreover, whether the dysfunctions of migrasome formation might be associated with miscarriage is also largely unclear.

Migrasome formation could be regulated by tetraspanin family proteins, such as TSPAN4 (tetraspanin 4) and TSPAN9(tetraspanin 9), which promote migrasome formation. Conversely, knockdown of these tetraspanins suppressed migrasome formation.^[^ ^35^ ^]^ Meanwhile, ROCK1 (Rho associated coiled coil‐containing protein kinase 1) could also regulate migrasome formation; and knockdown of ROCK1 reduces the number of migrasomes generated in zebrafish embryo.^[^ ^38^ ^]^ Moreover, NDST1 (bifunctional heparan sulfate N‐deacetylase/N‐sulfotransferase 1) has also been used as a marker for biochemical detection of migrasomes.^[^ ^39^ ^]^ However, whether other pathway might also function for migrasome formation still needs further exploration. PKCA (protein kinase C alpha) is an important kinase C family protein that regulates cell proliferation, invasion, migration, apoptosis, and cell cycle.^[^ ^40^ ^]^ The dysfunctions of PKCA are associated with many pathological processes.^[^ ^41^ ^]^ It has been reported in pancreatic acinar cells that PKCA activates the downstream RAC1 (Ras‐related C3 botulinum toxin substrate 1).^[^ ^42^, ^43^ ^]^ Moreover, RAC1 and CXCL12 (C‐X‐C motif chemokine ligand 12) also regulate the dynamics of cell motility and proliferation.^[^ ^44^, ^45^ ^]^ However, whether PKCA/RAC1/CXCL12 might regulate migrasome formation in trophoblast cells has never been reported.

LncRNAs (long non‐coding RNAs) epigenetically regulate various cellular and biological processes and are closely associated with the occurrence and development of multiple diseases.^[^ ^46^ ^]^ It has been reported that a certain of lncRNAs regulate trophoblast cell functions, such as lncRNA EPB41L4A‐AS1^[^ ^47^ ^]^ and lnc‐SLC4A1‐1.^[^ ^48^ ^]^ Recently, we have identified a group of novel lncRNAs, including lnc‐HZ01, lnc‐HZ03, lnc‐HZ04, lnc‐HZ05, lnc‐HZ06, lnc‐HZ08, lnc‐HZ09, lnc‐HZ10, lnc‐HZ11, lnc‐HZ12, and lnc‐HZ14, all of which regulate the dysfunctions of human trophoblast cells and the occurrence of miscarriage.^[^ ^27^, ^30^, ^49^, ^50^, ^51^, ^52^, ^53^, ^54^, ^55^, ^56^, ^57^, ^58^ ^]^ Among them, lnc‐HZ04 serves as a ceRNA for miR‐hz04, up‐regulates the IP3R1/CaMKII/SGCB pathway, and thus induces trophoblast cell apoptosis. In general, an lncRNA might have multiple functions. Whether and how lnc‐HZ04 might regulate migrasome formation in BPB‐exposed trophoblast cells through the PKCA/RAC1/CXCL12 pathway is still completely unknown.

In this study, we find that higher BPB levels in urine are associated with women unexplained miscarriage (UM) and BPB exposure induces miscarriage by suppressing migration/invasion and migrasome formation through down‐regulating PKCA/RAC1/CXCL12 signaling, as evidenced by human trophoblast cell assays, human villous tissues of an UM case‐control study, and BPB‐exposed mouse models. Lnc‐HZ04 regulates these cellular processes and lnc‐HZ04 levels in serum are associated with miscarriage. Supplement with Pkca or Tspan4 is efficient for treatment against BPB‐induced mouse miscarriage. This study not only discovers novel toxicological effects of BPB exposure on human reproduction but also provides potential targets for treatment against unexplained miscarriage.

## 2 Results

### 2.1 Epidemiological Analysis Showed that BPB Exposure was Associated with Unexplained Miscarriage

**Figure 1:**
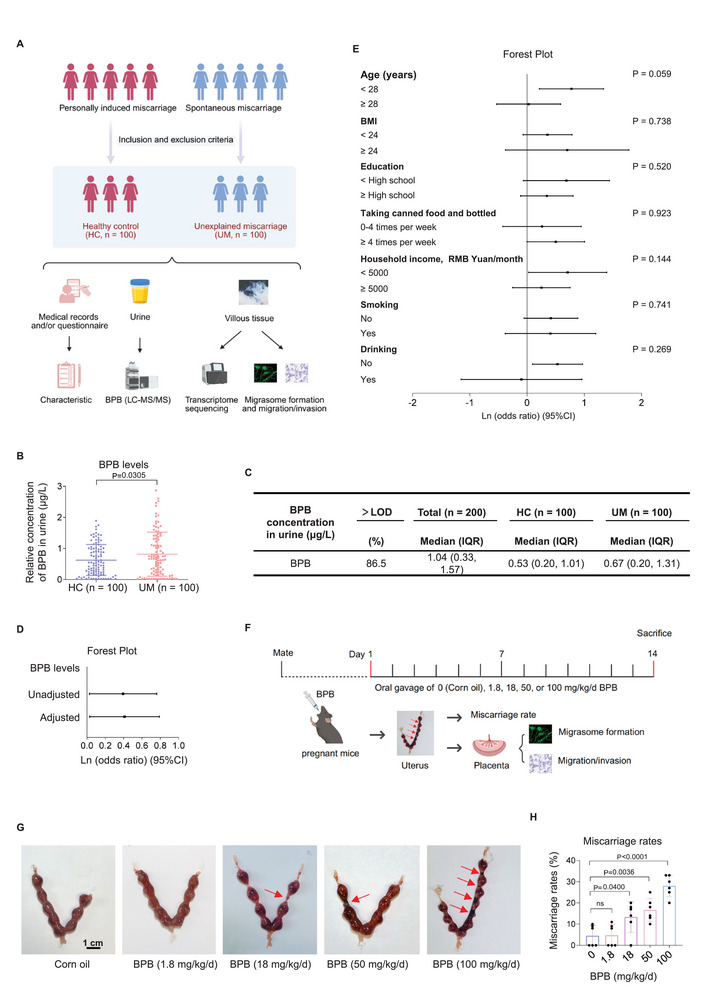
Epidemiological analysis showed that BPB exposure was associated with unexplained miscarriage. A) Schematic diagram for HC and UM case‐control studies. B) BPB concentrations in HC and UM urine samples (*n* = 100). C) Distribution of BPB concentrations (µg L^−1^) in HC and UM urine samples (each *n* = 100). LOD, limit of detection; IQR, interquartile range. D) Percent change (95% CI) levels of BPB in Table S2 (Supporting Information). E) Estimated associations between BPB levels and miscarriage by stratification analysis (all P for interaction >0.05). F) The scheme for the construction of a BPB‐exposed mouse model. G,H) Embryo resorption (H, indicated by red arrows, scale bar, 1 cm) and the average miscarriage rates (I, embryo resorption rates) in BPB‐exposed mice (each *n* = 6). Data were shown as means ± SD (standard deviation). *p* <0.05 meant significant differences compared with the control group.

To explore the potential correlation between BPB exposure and the occurrence of unexplained miscarriage (UM), we conducted a case‐control study containing 100 UM patients and 100 their matched healthy control (HC) group. The clinically known causes of miscarriage have been excluded, such as cervical incompetence, chromosome abnormalities, endocrine or metabolic diseases, virus or bacterial infections, et al., as described previously^[^ ^54^, ^59^ ^]^ (**Figure** **1**A). To reduce the effects of potential confounders, variables were also collected based on prior studies and their potential associations with miscarriage,^[^ ^60^, ^61^ ^]^ such as baseline characteristic, clinical information, and lifestyle (Table S1, Supporting Information). All the information was collected from medical records and/or questionnaire. These characteristics did not show significant differences between these HC and UM groups (Table S1, Supporting Information).

BPB levels were detected in urine samples of HC and UM women. To avoid potential BPB contaminants, glass tubes and instruments were used in urine storage, processing, and analysis. BPB had detection rates >86.5% in all urine samples. The median of BPB levels was 0.53 µg L^−1^ in the HC group and 0.67 µg L^−1^ in the UM group (Figure 1B,C). BPB levels were significantly higher in UM vs HC groups (Figure 1B,C). In an unadjusted model, univariate logistic regression analysis showed that higher levels of BPB (OR = 1.483, 95% CI, 1.027–2.141) were associated with miscarriage (Figure 1D; Table S2, Supporting Information). Based on directed acyclic graph analysis and previous literature,^[^ ^60^, ^61^ ^]^ the variables such as age, BMI, education, household income, smoking, drinking, and taking canned food and bottled drinks were considered as potential confounders (Figure S1A, Supporting Information). To reduce their effects, we further used multivariate logistic regression to analyze the association between BPB levels and miscarriage by adjusting for all these confounders, giving the adjusted OR value of 1.507 with 95% CI of 1.030–2.205 (Figure 1D; Table S2, Supporting Information). To further examine whether the estimated association differed among subpopulations, we found that the stratifying factors did not significantly alter the association between BPB levels and miscarriage (all P for interaction >0.05, Figure 1E), confirming the robustness of these results. Collectively, all the statistical analysis demonstrated that BPB exposure was positively associated with unexplained miscarriage.

### 2.2 Mouse Model Assays Confirmed that BPB Induced Miscarriage

To further explore the causation whether BPB exposure might induce miscarriage, we constructed a BPB‐exposed mouse model in which pregnant mice were exposed to 0, 1.8 mg kg^−1^ day^−1^ (1‐fold REED), 18 mg kg^−1^ day^−1^ (10‐fold REED), 50 mg kg^−1^ day^−1^ (28‐fold REED, 1/5 of LD_50_, medium‐dose group), or 100 mg kg^−1^ day^−1^ (56‐fold REED, 2/5 of LD_50_, high‐dose group) BPB by oral gavage (Figure 1F), as the method described previously.^[^ ^22^, ^62^ ^]^ BPB exposure reduced the body gain of mice on D14 relative to initial body weight on D0 (Figure S1B,C, Supporting Information), increased embryo absorption (Figure 1G), and elevated miscarriage rates (Figure 1H; Figure S1D, Supporting Information), indicating that BPB exposure could induce mouse miscarriage.

### 2.3 mRNA Sequencing Analysis Indicated that BPB Exposure, Cell Migration, and Miscarriage were Closely Associated

**Figure 2:**
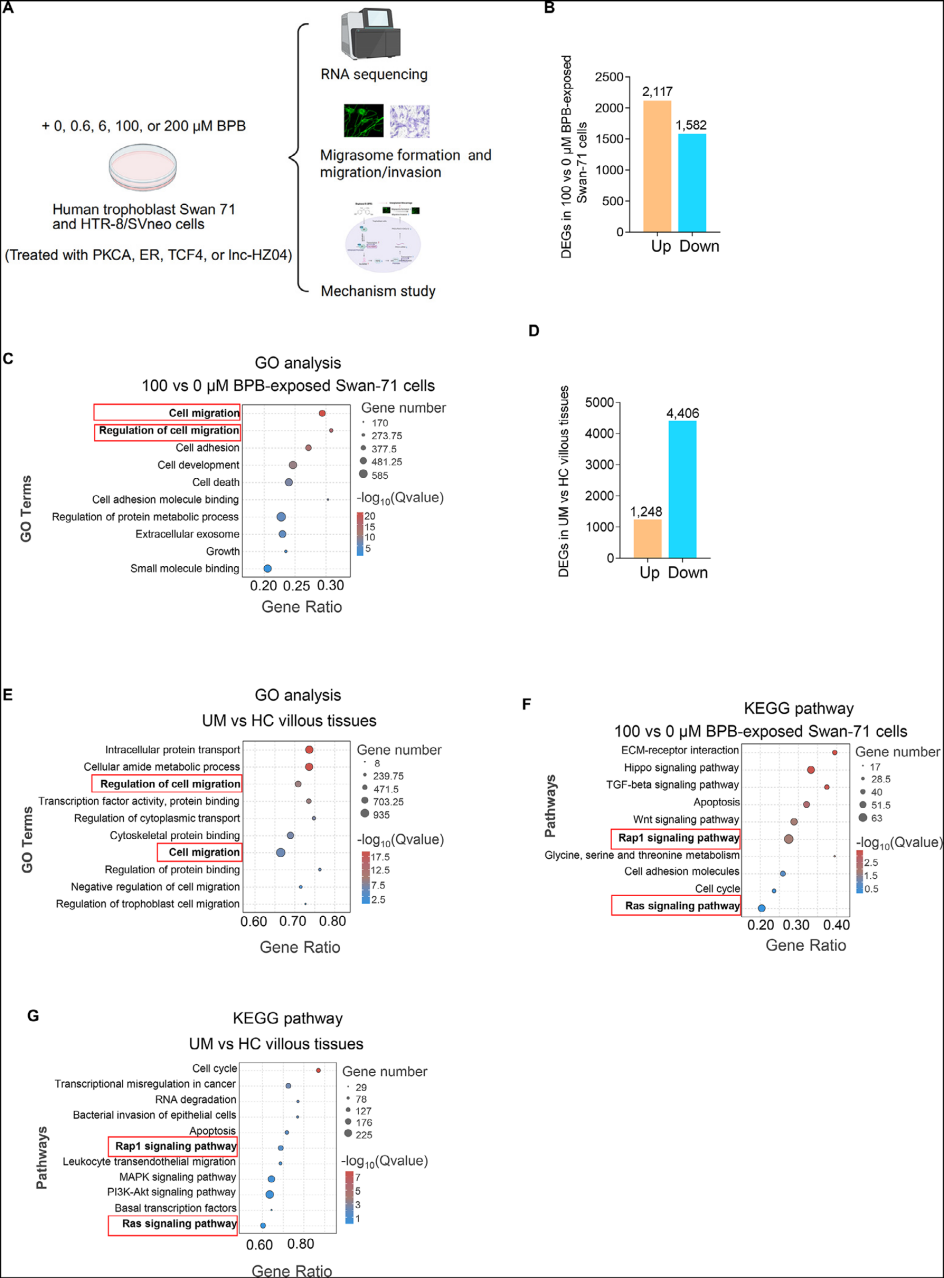
mRNA sequencing analysis showed that BPB exposure, altered cell migration, and miscarriage were closely associated. A) Schematic diagram for cellular studies with BPB‐exposed Swan 71 and HTR‐8/SVneo cells. B) The number of differentially expressed mRNAs in 100 µM BPB‐exposed Swan 71 cells compared with the control cells. C) GO analysis of the differentially expressed mRNAs in BPB‐exposed Swan 71 cells vs control cells. D) The number of differentially expressed mRNAs in UM vs HC villous tissues. E) GO analysis of the differentially expressed mRNAs in UM vs HC villous tissues. F) KEGG pathway analysis of the differentially expressed mRNAs in BPB‐exposed Swan 71 cells vs control cells. G) KEGG pathways analysis of the differentially expressed mRNAs in UM vs HC villous tissues. DEGs: differentially expressed genes, HC, healthy control, UM, unexplained miscarriage.

Trophoblast cells play important roles in a healthy pregnancy. Dysfunctions of human trophoblast cells might induce miscarriage. To correlate whether BPB exposure might induce miscarriage through the dysfunctions of human trophoblast cells, we treated trophoblast Swan 71 cells with 100 vs 0 µm BPB (**Figure** **2**A) and used them, together with UM vs HC villous tissues, for mRNA sequencing. In BPB‐exposed Swan 71 cells, there were 2117 up‐regulated and 1582 down‐regulated mRNAs with differences > 2‐fold and *p* values <0.05 (Figure 2B). GO analysis of these differentially expressed mRNAs (DEMs) showed that BPB exposure significantly altered trophoblast cell migration, growth, cell death, and cell adhesion (Figure 2C). In UM and HC villous tissues, there were 1248 up‐regulated and 4406 down‐regulated mRNAs with differences >2‐fold and *p* values <0.05 (Figure 2D). GO analysis of these DEMs showed that migration, protein transport, cellular amide metabolism, and transcription factor activity were significantly different in UM vs HC villous tissues (Figure 2E). Therefore, both GO analysis suggested that migration/invasion (MI) and migrasome formation (MF) might be simultaneously regulated. Meanwhile, KEGG pathway analysis of DEGs in BPB‐exposed vs unexposed Swan 71 cells (Figure 2F) and in UM vs HC villous tissues (Figure 2G) showed that Rap1 signaling and Ras signaling were significantly enriched. It has been reported that Rap1 signaling activates RAC1^[^ ^63^ ^]^ and RAC1 also belongs to the Ras superfamily.^[^ ^64^ ^]^ Meanwhile, PKCA activates its downstream RAC1^[^ ^42^, ^43^ ^]^; and both RAC1 and CXCL12 regulate cell migration and invasion.^[^ ^44^, ^45^ ^]^ Therefore, KEGG analysis suggested that PKCA/RAC1/CXCL12 signaling, which might function for cell MI and MF, might be simultaneously regulated in BPB‐exposed trophoblast cells and in UM vs HC villous tissues. These results indicate that BPB exposure, the altered MI and MF in human trophoblast cells, and miscarriage might be closely correlated. High possibly, BPB might induce miscarriage by suppressing human trophoblast cell MI and MF through this signaling pathway.

### 2.4 MI, MF, and PKCA/RAC1/CXCL12 Pathway were Suppressed in BPB‐Exposed Trophoblast Cells and in UM vs HC Villous Tissues

To validate the sequencing data, we detected migration/invasion (MI), migrasome formation (MF), and the PKCA/RAC1/CXCL12 pathway in BPB‐exposed trophoblast cells and in UM vs HC villous tissues. We selected 0 (control), 0.6 µm (1‐fold REED), 6 µm (10‐fold REED), 100 µm (167‐fold REED), or 200 µm (333‐fold REED) BPB to construct BPB‐exposed human trophoblast cell models. Transwell assays showed that BPB exposure suppressed MI of Swan 71 and HTR‐8/SVneo cells in a BPB dose‐dependent manner (**Figure** **3**A,B; Figure S2A,B, Supporting Information). Migrasome assays also showed that MF in human trophoblast cells transfected with TSPAN4‐GFP, which is associated with migrasome formation^[^ ^35^ ^]^ and acts as a typical indicator protein, was also reduced after BPB exposure (Figure 3C,D). Moreover, the protein levels of TSPAN4 and NDST1, two typical protein markers associated with migrasome formation, were also reduced with increasing BPB concentrations (Figure 3E,F). The specificity of the TSPAN4 antibody was confirmed using two different si‐TSPAN4 and an overexpression plasmid of TSPAN4 in human Swan 71 and HTR‐8/SVneo cells (Figure S2C–E, Supporting Information). To validate whether this PKCA/RAC1/CXCL12 pathway functioned for MI and MF, we overexpressed or silenced PKCA in Swan 71 or HTR‐8 cells (Figure 3G,H; Figure S2F–I, Supporting Information). We found that overexpression of PKCA up‐regulated, whereas knockdown of PKCA down‐regulated, the mRNA and protein levels of RAC1 and CXCL12 (Figure 3G,H; Figure S2F–I, Supporting Information). Meanwhile, overexpression of PKCA promoted, whereas knockdown of PKCA suppressed, MI and MF (Figure 3I–N; Figure S2J,K, Supporting Information). Therefore, these results confirmed that PKCA/RAC1/CXCL12 pathways promoted MI and MF in human trophoblast cells. In BPB‐exposed human trophoblast cells, the mRNA and protein levels of PKCA, RAC1, and CXCL12 were all reduced with increasing BPB exposure doses (Figure 3O,P; Figure S2L–N, Supporting Information). Therefore, BPB exposure suppressed MI and MF by down‐regulating the PKCA/RAC1/CXCL12 pathway.

Figure 3MI and MF were suppressed in BPB‐exposed trophoblast cells and in UM vs HC villous tissues. A,B) Transwell assay analysis of MI of BPB‐exposed Swan 71 cells (scale bar, 200 µm), and the migrated or invaded cells per view were quantified. C,D) Confocal fluorescent image of MF of 100 µm BPB‐exposed Swan 71 cells overexpressing TSPAN4‐GFP (C, Scale bar, 10 µm) and their quantification (D, *n* = 100 cells). E,F) Western blot analysis of the protein levels of TSPAN4 and NDST1 (MF‐associated biomarkers) and their relative quantification in BPB‐exposed Swan 71 cells. G,H) Western blot analysis of the protein levels of PKCA, RAC1, and CXCL12 in Swan 71 cells with PKCA overexpression or knockdown, with GAPDH as internal standard, and their relative quantification. I,J) Transwell assay analysis (I) of MI of Swan 71 cells with PKCA overexpression or knockdown (scale bar, 200 µm) and their quantification (J). K,L) Confocal fluorescent image (K) of MF of Swan 71 cells overexpressing TSPAN4‐GFP and silencing PKCA (Scale bar, 10 µm) and the quantification (L) of migrasomes per cell (n = 100 cells). M,N) Western blot analysis (M) of the protein levels of TSPAN4 and NDST1 (MF‐associated biomarkers) in Swan 71 cells with PKCA overexpression or knockdown, with GAPDH as internal standard, and their relative quantification (N). O,P) Western blot analysis (O) of the protein levels of PKCA, RAC1, and CXCL12 in BPB‐exposed Swan 71 cells, with GAPDH as internal standard, and their relative quantification (P). Q,R) Western blot analysis (Q) of the protein levels of PKCA, RAC1, CXCL12, TSPAN4, and NDST1 in HC and UM villous tissues, with GAPDH as internal standard, and their relative quantification (R, each *n* = 12). S,T) IHC analysis of TSPAN4 (S) and NDST1 (T) protein levels in HC and UM villous tissues (scale bar, 50 µm) and its relative quantification (*n* = 12). U) WGA staining of HC and UM villous tissues (scale bar, 50 µm). In WB assays, equal amounts of proteins within group but different amounts of proteins among groups were used for better comparison. Data were shown as means ± SD (standard deviation). *p* <0.05 meant significant differences compared with the control.

**Figure:**
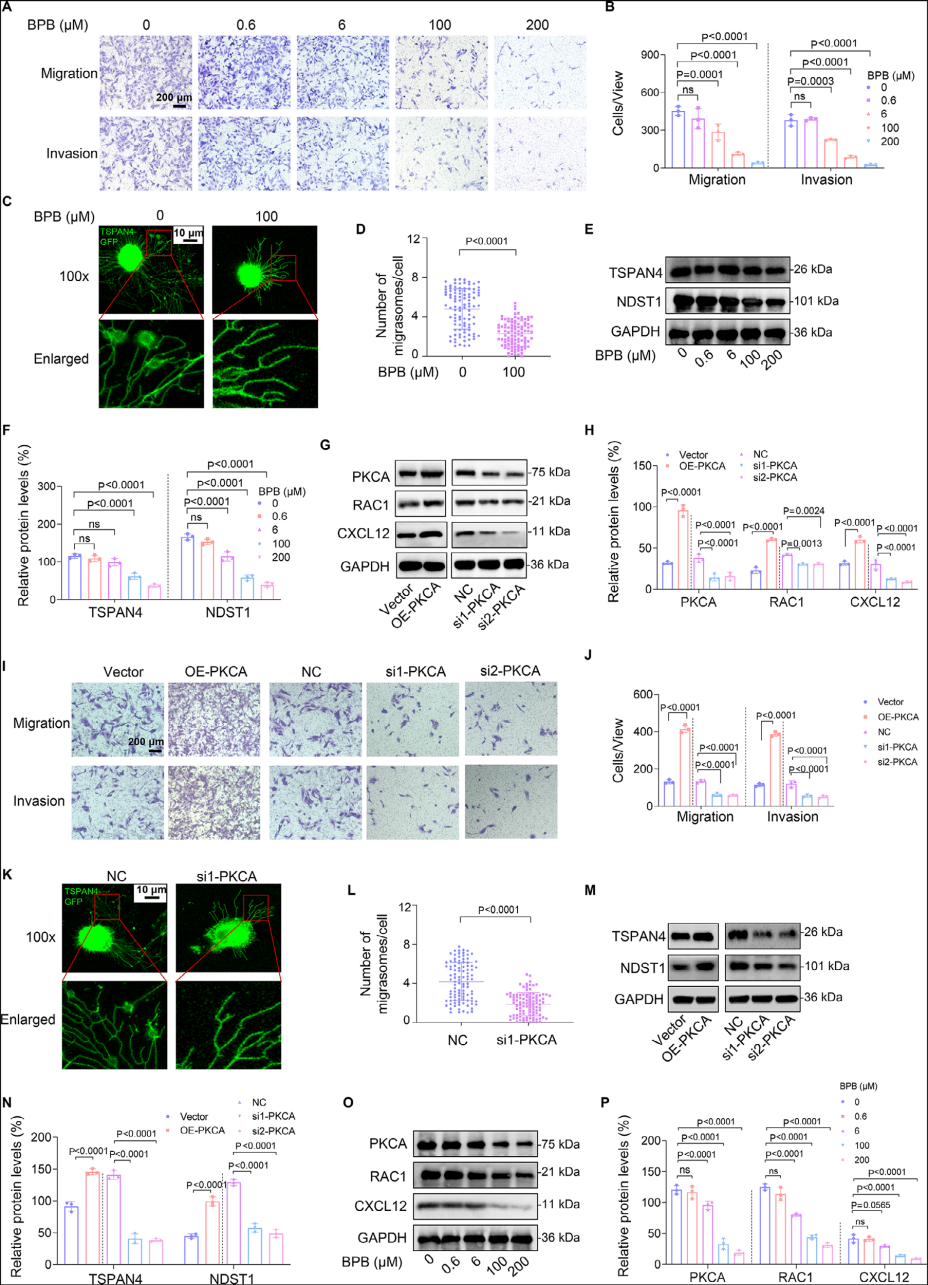

**Figure:**
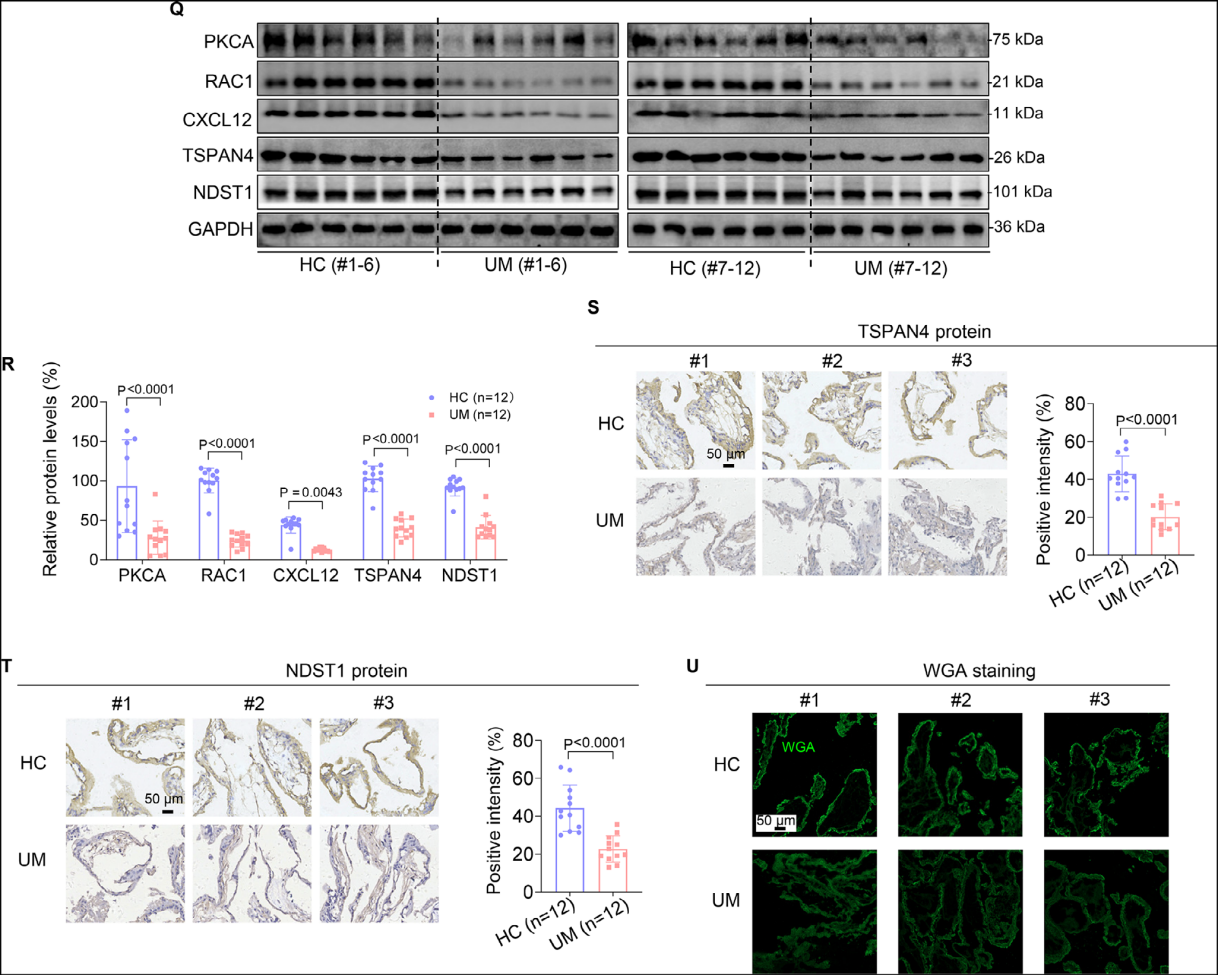

In villous tissues, the mRNA and protein levels of the members in the PKCA/RAC1/CXCL12 pathway were lower in UM vs the HC group (Figure 3Q,R; Figure S2O,P, Supporting Information). Meanwhile, MF was also suppressed as indicated by lower expression levels of TSPAN4 and NDST1 (MF‐associated biomarkers) and lower staining levels of wheat germ agglutinin (WGA, indicator of retraction fibers and migrasomes)^[^ ^65^, ^66^ ^]^ in UM vs HC groups (Figure 3S–U). These results confirmed that MI and MF were suppressed in UM vs HC villous tissues. Moreover, multivariate logistic regression analysis by adjusting for all these confounders (Figure S1A, Supporting Information) also showed that lower mRNA levels of PKCA were positively associated with UM (the adjusted OR = 0.286, 95% CI, 0.141–0.580, Figure S2Q and Table S2, Supporting Information). Therefore, these results confirmed that the lower expression levels of PKCA and suppressed MI and MF were associated with unexplained miscarriage.

Collectively, these results indicated that BPB exposure down‐regulated the PKCA/RAC1/CXCL12 pathway and thus suppressed MI and MF in human trophoblast cells, which might induce miscarriage.

### 2.5 BPB Exposure Induced Miscarriage by Suppressing MI and MF through Down‐Regulating PKCA/RAC1/CXCL12 Pathway in Mouse Model

**Figure 4:**
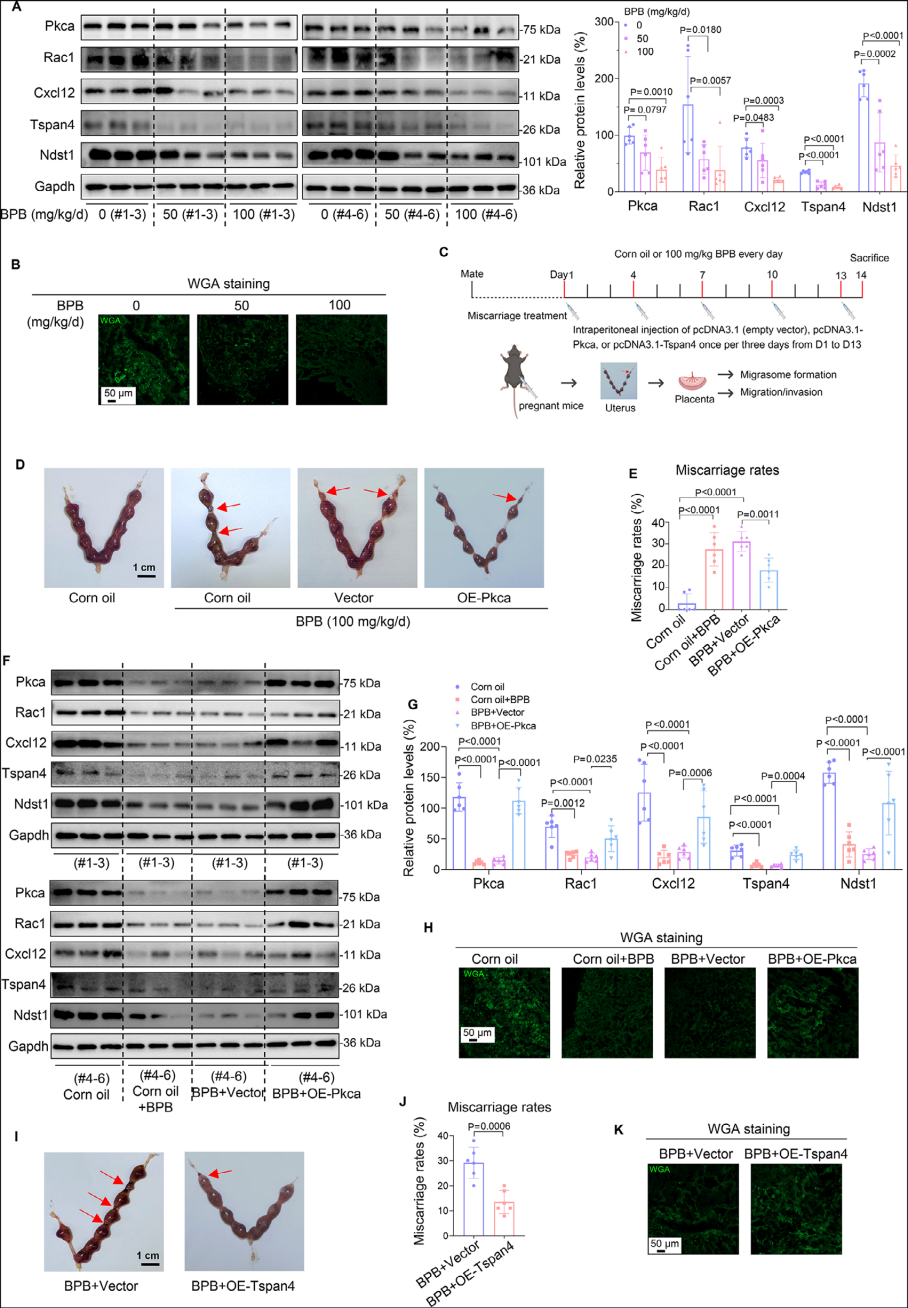
Mouse model assays further confirmed that BPB exposure induced miscarriage by directly suppressing MI and MF. A) Western blot analysis of the protein levels of murine Pkca, Rac1, Cxcl12, Tspan4, and Ndst1 in BPB‐exposed mouse placental tissues, with Gapdh as internal standard, and their relative quantification (each *n* = 6). B) WGA staining of BPB‐exposed mouse placenta tissues (scale bar, 50 µm). C) The scheme for the construction of a mouse miscarriage intervention model. D,E) Embryo resorption (D, indicated by red arrows, scale bar, 1 cm) and the average miscarriage rates (E) in 100 mg kg^−1^ d BPB‐exposed mice with Pkca supplement (each *n* = 6). F,G) Western blot (F) analysis of the protein levels of Pkca, Rac1, Cxcl12, Tspan4, and Ndst1 in placental tissues in 100 mg kg^−1^ d^−1^ BPB‐exposed mice with supplement with Pkca, with Gapdh as internal standard, and their relative quantification (G, each *n* = 6). H) WGA staining of the placenta tissues of BPB‐exposed mice with supplement with Pkca (scale bar, 50 µm). I,J) Embryo resorption (I, indicated by red arrows, scale bar, 1 cm) and the average miscarriage rates (J) in 100 mg kg^−1^ d^−1^ BPB‐exposed mice with Tspan4 supplement (each *n* = 6). K) WGA staining of the placenta tissues of BPB‐exposed mice with supplement with Tspan4 (scale bar, 50 µm). Data were shown as means ± SD (standard deviation). *p* <0.05 meant significant differences compared with the control.

To directly explore whether BPB exposure might induce miscarriage by suppressing MI and MF, we went back to this BPB‐exposed mouse model. The mRNA and amino acid sequences of PKCA, RAC1, CXCL12, TSPAN4, and NDST1 were all conserved in rhesus, mouse, dog, and elephant (Figure S3A and Table S3, Supporting Information). Their expression levels, as well as WGA staining levels, were all decreased in placental tissues of BPB‐exposed mice in a BPB concentration‐dependent manner (**Figure** **4**A,B; Figure S3B–D, Supporting Information). These results showed that BPB exposure down‐regulated the murine Pkca/Rac1/Cxcl12 pathway, suppressed placental MI and MF, and further caused miscarriage.

To further investigate the causation whether the suppressed MI and MF might cause miscarriage, we constructed a mouse miscarriage intervention model, in which BPB‐exposed pregnant mice were intraperitoneally injected with murine Pkca overexpression plasmid, with empty vector as control (Figure 4C). Its expression efficiency was validated using mouse trophoblast cells (Figure S3E, Supporting Information). As control, intraperitoneal injection of empty vector plasmid did not obviously affect mouse hair/behavioral phenotypes (Figure S3F, Supporting Information) and the expression levels of pro‐inflammatory cytokines (such as TNF‐α, IL‐6, IL‐1β) in serum (Figure S3G–I, Supporting Information), showing that this intraperitoneal plasmid delivery might not cause detectable systemic toxicity. To evaluate the delivery specificity, we found that Pkca protein levels were significantly higher in the tissues of uterus, placenta, fetus, small intestine, and liver but were little changed in the tissues of brain, heart, or lung in pregnant mice treated with Pkca overexpression plasmid (Figure S3J,K, Supporting Information), showing that this injection might promote Pkca expression in intraperitoneal organs (including placenta) but less affect its expression in other organs. In placental tissues, we found that Pkca protein was highly expressed and was also co‐localized with Krt7 protein, a typical indicator of mouse trophoblast cells^[^ ^67^ ^]^ (Figure S3L, Supporting Information), indicating that Pkca could be overexpressed in mouse placental trophoblast cells.

BPB exposure reduced the body gain on D14 relative to that on D1 (Figure S3M,N, Supporting Information), increased embryo adsorption, and elevated miscarriage rates (Figure 4D,E; Figure S3O, Supporting Information). However, all these changes were reversed after supplement with murine Pkca. Analysis of the placental tissues showed that BPB exposure reduced the protein levels of murine Pkca, Rac1, Cxcl12, Tspan4, and Ndst1, as well as the levels of WGA staining (Figure 4F–H; Figure S3P–R, Supporting Information). However, all these reductions were reversed after supplement with Pkca. Collectively, these results indicated that the recovery of MI and MF by supplement with Pkca could efficiently alleviate miscarriage in the BPB‐exposed mouse miscarriage model.

Subsequently, we explored whether supplement with murine Tspan4 could also alleviate miscarriage in these BPB‐exposed mice. For this aim, we also constructed a miscarriage intervention model in which BPB‐exposed pregnant mice were intraperitoneally injected with a plasmid overexpressing murine Tspan4, with an empty vector as a control, once per three days (Figure 4C). Experimentally, we confirmed the specificity of the TSPAN4 antibody and the murine Tspan4 expression efficiency in mouse trophoblast cells with overexpression or knockdown of murine Tspan4 (Figure S3S–U, Supporting Information). Relative to the BPB‐exposed mouse control group, the body gain on D14 relative to that on D1 was increased (Figure S3V,W, Supporting Information) and the miscarriage rates were reduced in the Tspan4‐supplemented mouse group (Figure 4I,J; Figure S3X, Supporting Information). Analysis of the placental tissues showed that the protein levels of murine Tspan4 and Ndst1 (MF‐associated biomarkers) and the WGA staining levels (indicator of retraction fibers and migrasomes) were all restored in the Tspan4‐supplemented group relative to those in the control group (Figure 4K; Figure S3Y–AD, Supporting Information). Collectively, these results indicated that the recovery of MF by supplement with Tspan4 could also efficiently alleviate miscarriage in the BPB‐exposed mouse miscarriage model.

Collectively, these results supported that BPB exposure induced miscarriage by suppressing MI and MF through down‐regulating the PKCA/RAC1/CXCL12 pathway. Supplement with murine Pkca or Tspan4 was efficient for treatment against BPB‐induced mouse miscarriage.

### 2.6 Lnc‐HZ04 Suppressed MI and MF by Down‐Regulating PKCA/RAC1/CXCL12 Pathway

LncRNAs play important roles in the regulation of various cellular processes. We expected to explore whether MI and MF might be regulated by lncRNAs. In sequence data of BPB‐exposed Swan 71 cells, there were 1422 up‐regulated and 1299 down‐regulated lncRNAs with differences >2‐fold and *p* values <0.05 (Figure S4A, Supporting Information). In sequence data of UM and HC villous tissues, there were 411 up‐regulated and 811 down‐regulated lncRNAs with differences >2‐fold and *p* values <0.05 (Figure S4B, Supporting Information). In the intersection of these up‐regulated lncRNAs (**Figure** **5**A), we identified lnc‐HZ04 that was one of the most highly expressed lncRNAs in BPB‐exposed human trophoblast cells and in UM vs HC villous tissues, which was further confirmed by RT‐qPCR (Figure 5B,C; Figure S4C, Supporting Information). To explore its association with miscarriage, multivariate logistic regression analysis by adjusting for all these confounders (Figure S1A, Supporting Information) showed higher levels of lnc‐HZ04 in villous tissues were positively associated with miscarriage (Figure 5D; Table S2, Supporting Information). To explore its regulatory roles, we found that overexpression of lnc‐HZ04 down‐regulated the expression levels of the members in the PKCA/RAC1/CXCL12 pathway, and knockdown of lnc‐HZ04 up‐regulated their expression levels (Figure 5E,F; Figure S4D–G, Supporting Information). Moreover, overexpression of lnc‐HZ04 suppressed MI and MF, and knockdown of lnc‐HZ04 promoted MI and MF (Figure 5G–I; Figure S4H,I, Supporting Information). Taken together, these results demonstrated that lnc‐HZ04 suppressed MI and MF by down‐regulating the PKCA/RAC1/CXCL12 pathway in human trophoblast cells.

Figure 5Lnc‐HZ04, up‐regulated in BPB‐exposed trophoblast cells and in UM vs HC villous tissues, suppressed MI and MF. A) The significantly differentially expressed lncRNAs in the intersection of transcriptome sequencing data of 100 µm BPB‐exposed vs control cells and UM vs HC villous tissues. B,C) RT‐qPCR analysis of lnc‐HZ04 levels in BPB‐exposed Swan 71 cells (B, *n* = 3) or in UM vs HC villous tissues (C, *n* = 50). D) Percent change (95% CI) levels of lnc‐HZ04. E,F) The protein levels (E) of PKCA, RAC1, and CXCL12 in Swan 71 cells with lnc‐HZ04 overexpression or knockdown, with GAPDH as internal standard, and their relative quantification (F). G) Transwell assay analysis of MI of Swan 71 cells with lnc‐HZ04 overexpression or knockdown (scale bar, 200 µm) and their quantification. H) Confocal fluorescent image of MF of Swan 71 cells overexpressing TSPAN4‐GFP and lnc‐HZ04 (Scale bar, 10 µm) and their quantification (*n* = 100 cells). I) Western blot analysis of the protein levels of TSPAN4 and NDST1 (MF‐associated biomarkers) in Swan 71 cells with lnc‐HZ04 overexpression or knockdown and their relative quantification. J,K) Western blot analysis of the protein levels of PKCA, RAC1, CXCL12, TSPAN4, and NDST1 in 100 µm BPB‐exposed Swan 71 cells with lnc‐HZ04 overexpression or knockdown, with GAPDH as internal standard, and their relative quantification. L) Transwell assay analysis of MI of 100 µm BPB‐exposed Swan 71 cells with lnc‐HZ04 overexpression or knockdown (scale bar, 200 µm) and their quantification. M–O) The correlation analysis between the levels of lnc‐HZ04 and the protein levels of PKCA, TSPAN4, or NDST1 in HC and UM villous tissues (each *n* = 12). In WB assays, equal amounts of proteins within group but different amounts of proteins among groups were used for better comparison. Data were shown as means ± SD (standard deviation). *p* <0.05 meant significant differences compared with the control.

**Figure:**
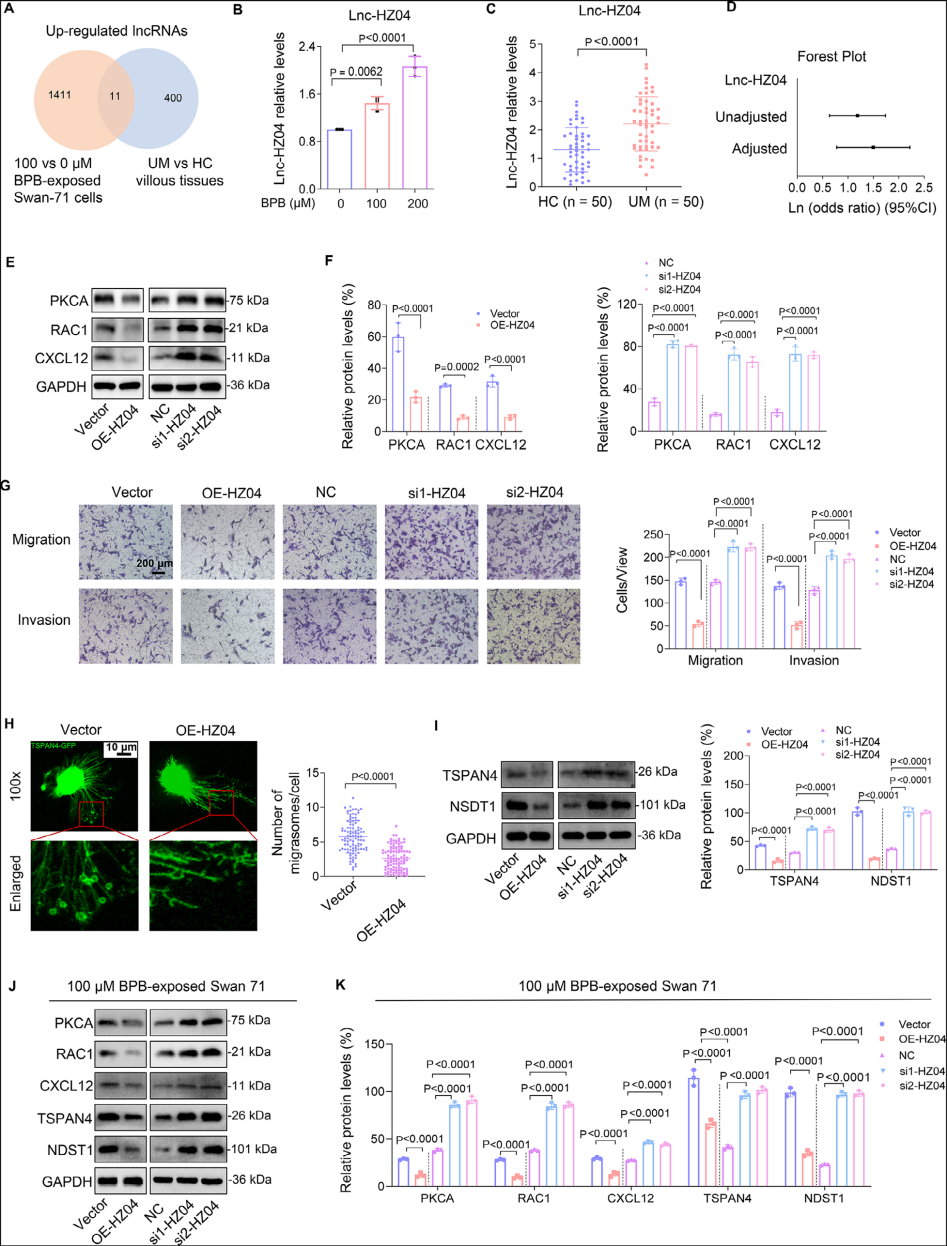

**Figure:**
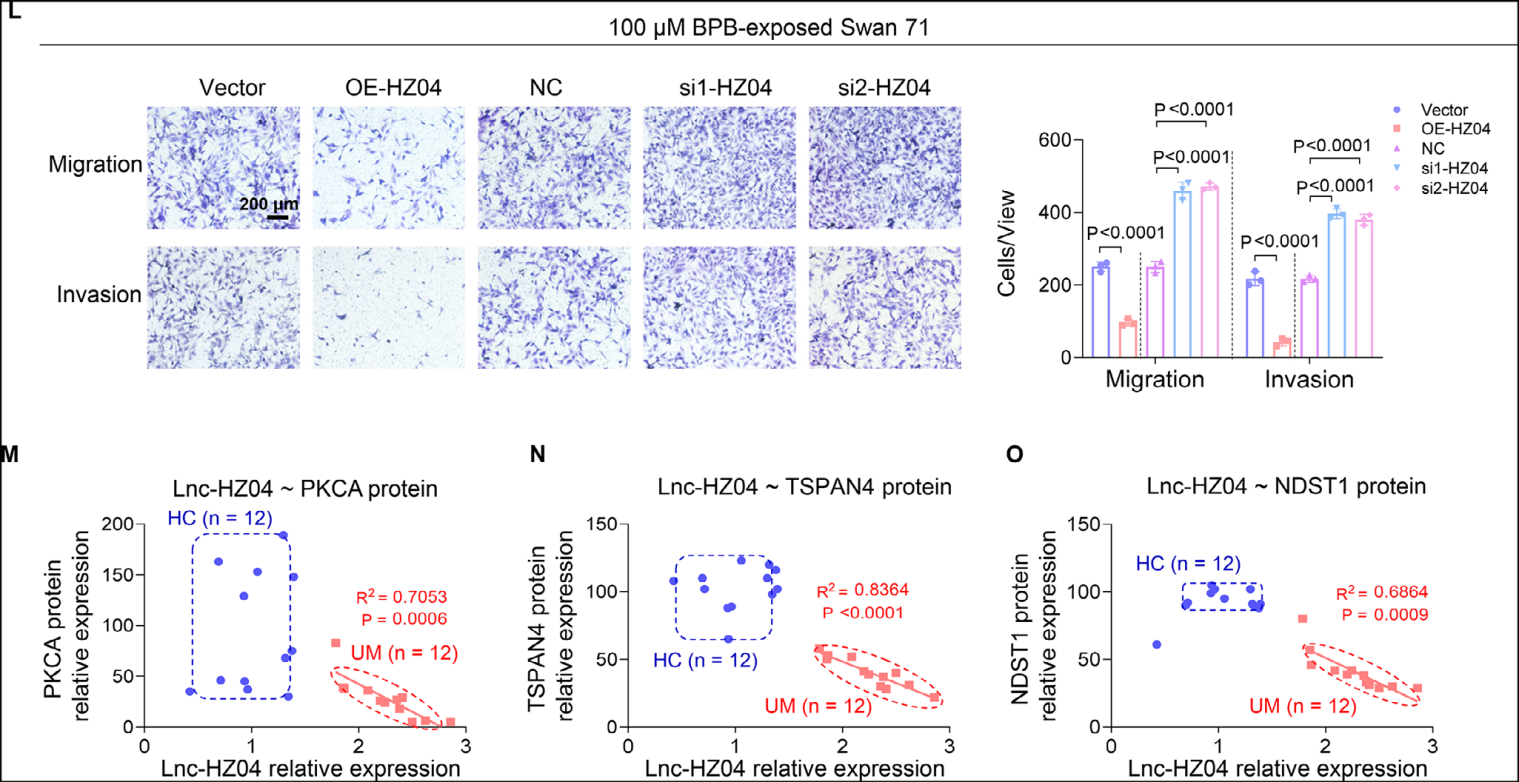

In BPB‐exposed human trophoblast cells, lnc‐HZ04 showed consistent results. Overexpression of lnc‐HZ04 down‐regulated this PKCA/RAC1/CXCL12 pathway and suppressed MI and MF; whereas knockdown of lnc‐HZ04 up‐regulated this pathway and promoted MI and MF (Figure 5J–L; Figure S5A–E, Supporting Information). Therefore, BPB exposure up‐regulated lnc‐HZ04 expression levels, which suppressed MI and MF by down‐regulating the PKCA/RAC1/CXCL12 pathway in BPB‐exposed human trophoblast cells.

In villous tissues, lnc‐HZ04 was highly expressed in UM vs HC villous tissues (Figure 5C). After normalization of their relative expression levels, the levels of lnc‐HZ04 were negatively and linearly correlated with the protein levels of PKCA, RAC1, CXCL12, TSPAN4, and NDST1 in the UM group (Figure 5M–O; Figure S5F,G, Supporting Information). The data points in the HC group were separated from those in the UM group. Therefore, combined with the cellular results, lnc‐HZ04 might suppress MI and MF in UM villous tissues. However, in the mouse system, there was no lnc‐HZ04 analogue by sequencing alignment.

Collectively, lnc‐HZ04 was up‐regulated in BPB‐exposed trophoblast cells and in UM vs HC villous tissues. Lnc‐HZ04 suppressed migration/invasion and migrasome formation by down‐regulating the PKCA/RAC1/CXCL12 pathway.

### 2.7 Lnc‐HZ04 Suppressed TCF4‐Mediated PKCA Transcription

**Figure 6:**
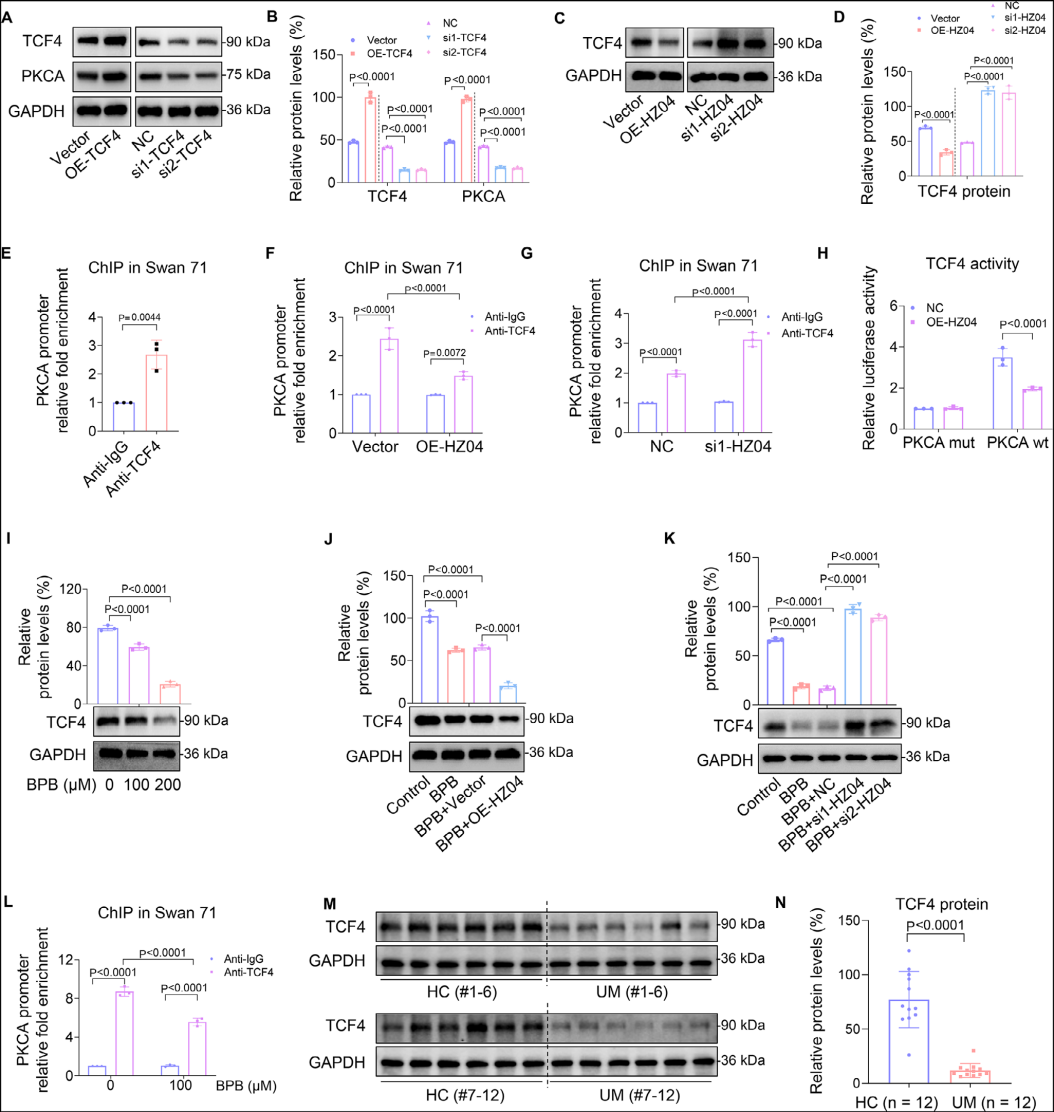
Lnc‐HZ04 suppressed TCF4‐mediated PKCA transcription. A,B) Western blot analysis of the protein levels of TCF4 and PKCA levels in Swan 71 cells with TCF4 overexpression or knockdown and their relative quantification. C,D) Western blot analysis of the protein levels of TCF4 levels in Swan 71 cells with lnc‐HZ04 overexpression or knockdown and their relative quantification. E) TCF4 ChIP assay analysis of the levels of PKCA promoter region enriched by TCF4 in Swan 71 cells. F,G) TCF4 ChIP assay analysis of the levels of PKCA promoter region enriched by TCF4 in Swan 71 cells with lnc‐HZ04 overexpression (F) or knockdown (G). H) Dual‐luciferase reporter assay analysis of the transcription activity of TCF4 using wild‐type (wt) or mutant (mut) promoter sequence of PKCA (sequences in Table S13, Supporting Information) in Swan 71 cells with lnc‐HZ04 overexpression. I) Western blot analysis of the protein levels of TCF4 in BPB‐exposed Swan 71 cells and its relative quantification. J) Western blot analysis of the protein levels of TCF4 in unexposed or 100 µm BPB‐exposed Swan 71 cells with overexpression of lnc‐HZ04 and its relative quantification. K) Western blot analysis of the protein levels of TCF4 in unexposed or 100 µm BPB‐exposed Swan 71 cells with knockdown of lnc‐HZ04 and its relative quantification. L) TCF4 ChIP assay analysis of the levels of PKCA promoter region enriched by TCF4 in 100 µm BPB‐exposed Swan 71 cells. M,N) Western blot (M) analysis of the protein levels of TCF4 in HC and UM villous tissues and its relative quantification (N, each *n* = 12). In WB assays, equal amounts of proteins within group but different amounts of proteins among groups were used for better comparison. Data were shown as means ± SD (standard deviation). *p* <0.05 meant significant differences compared with the control.

Subsequently, we explored how lnc‐HZ04 down‐regulated PKCA expression levels. As analyzed by PROMO software, TCF4 might be a transcription factor of PKCA. Overexpression of TCF4 promoted, whereas knockdown of TCF4 suppressed, the mRNA and protein levels of PKCA in trophoblast cells (**Figure** **6**A,B; Figure S6A–D, Supporting Information). We also found that overexpression of lnc‐HZ04 reduced TCF4 mRNA and protein levels, while knockdown of lnc‐HZ04 increased TCF4 mRNA and protein levels (Figure 6C,D; Figure S6E–G, Supporting Information). TCF4 ChIP assays showed that the promoter region of PKCA could be enriched by TCF4 (Figure 6E; Figure S6H, Supporting Information). Furthermore, overexpression of lnc‐HZ04 weakened this enrichment, whereas knockdown of lnc‐HZ04 further enhanced this enrichment (Figure 6F,G; Figure S6I,J, Supporting Information). Dual‐luciferase assays showed that TCF4 showed transcription activity using wild‐type but not mutant sequence in the PKCA promoter region, and this transcription activity was suppressed with lnc‐HZ04 overexpression (Figure 6H; Figure S6K, Supporting Information). Taken together, these results suggested that TCF4 acted as a transcription factor of PKCA and promoted its transcription, and lnc‐HZ04 suppressed TCF4‐mediated PKCA transcription in human trophoblast cells.

In BPB‐exposed trophoblast cells, the mRNA and protein expression levels of TCF4 were reduced, which were further decreased with lnc‐HZ04 overexpression but were recovered with lnc‐HZ04 knockdown (Figure 6I–K; Figure S6L–T, Supporting Information). TCF4 ChIP assays showed that the levels of the promoter region of PKCA enriched by TCF4 was reduced in BPB‐exposed trophoblast cells (Figure 6L; Figure S6U, Supporting Information). In tissues, the mRNA and protein levels of TCF4 were all lower in UM vs HC villous tissues (Figure 6M,N; Figure S6V, Supporting Information). Correlation analysis showed that TCF4 protein levels were positively correlated with those of PKCA but were negatively correlated with those of lnc‐HZ04 in UM villous tissues (Figure S6W,X, Supporting Information). Therefore, these results suggested that BPB exposure up‐regulated lnc‐HZ04, down‐regulated TCF4 expression levels, and thus suppressed TCF4‐mediated PKCA transcription.

### 2.8 Knockdown of lnc‐HZ04 or Supplement with PKCA Recovered MI and MF in BPB‐Exposed Trophoblast Cells

Based on the regulatory mechanisms, we further explored whether lnc‐HZ04 and PKCA might rescue MI and MF in BPB‐exposed trophoblast cells. First, BPB exposure suppressed MI and MF, a suppression that was abolished by co‐knockdown of lnc‐HZ04 or co‐overexpression of PKCA in BPB‐exposed trophoblast cells (**Figure** **7**A–D; Figure S7A,B, Supporting Information). However, co‐overexpression of lnc‐HZ04 or co‐knockdown of PKCA further suppressed MI in BPB‐exposed trophoblast cells (Figure S7C–F, Supporting Information). Moreover, BPB exposure down‐regulated the expression levels of the members in this PKCA/RAC1/CXCL12 pathway, and the down‐regulation were all reversed by co‐knockdown of lnc‐HZ04 or co‐overexpression of PKCA in BPB‐exposed trophoblast cells (Figure 7E–H; Figure S8A–H, Supporting Information). However, co‐overexpression of lnc‐HZ04 or co‐knockdown of PKCA further down‐regulated this pathway in BPB‐exposed trophoblast cells (Figure 7I–L; Figure S8I–P, Supporting Information). These results suggested that knockdown of lnc‐HZ04 or supplement with PKCA could efficiently alleviate BPB‐suppressed MI and MF in trophoblast cells, indicating that BPB exposure suppressed MI and MF by up‐regulating lnc‐HZ04 and down‐regulating PKCA/RAC1/CXCL12 pathway.

Figure 7Knockdown of lnc‐HZ04 or supplement with PKCA recovered MI and MF in BPB‐exposed trophoblast cells. A) Transwell assay analysis of MI of 100 µm BPB‐exposed Swan 71 cells with lnc‐HZ04 knockdown (scale bar, 200 µm) and their quantification. B) Confocal fluorescent image of MF of 100 µm BPB‐exposed and/or lnc‐HZ04‐silenced Swan 71 cells overexpressing TSPAN4‐GFP (Scale bar, 10 µm) and their quantification (*n* = 100 cells). C) Transwell assay analysis of MI of 100 µm BPB‐exposed Swan 71 cells with overexpression of PKCA (scale bar, 200 µm) and their quantification. D) Confocal fluorescent image of MF of 100 µm BPB‐exposed and/or PKCA‐overexpressed Swan 71 cells overexpressing TSPAN4‐GFP (Scale bar, 10 µm) and their quantification (*n* = 100 cells). E,F) Western blot analysis (E) of the protein levels of PKCA, RAC1, CXCL12, TSPAN4, and NDST1 in 100 µm BPB‐exposed Swan 71 cells with knockdown of lnc‐HZ04 and their relative quantification (F). G,H) Western blot analysis (G) of the protein levels of PKCA, RAC1, CXCL12, TSPAN4, and NDST1 in 100 µm BPB‐exposed Swan 71 cells with overexpression of PKCA and their relative quantification (H). I,J) Western blot analysis (I) of the protein levels of PKCA, RAC1, CXCL12, TSPAN4, and NDST1 in 100 µm BPB‐exposed Swan 71 cells with overexpression of lnc‐HZ04 and their relative quantification (J). K,L) Western blot analysis (K) of the protein levels of PKCA, RAC1, CXCL12, TSPAN4, and NDST1 in 100 µm BPB‐exposed Swan 71 cells with knockdown of PKCA and their relative quantification (L). M,N) Western blot analysis (M) of PKCA protein levels in Swan 71 cells with overexpression of lnc‐HZ04 and/or PKCA and its relative quantification (N). O) Transwell assay analysis of MI of Swan 71 cells with overexpression of lnc‐HZ04 and/or PKCA (scale bar, 200 µm) and their quantification. P) Confocal fluorescent image of MF of Swan 71 cells overexpressing TSPAN4‐GFP with co‐overexpression of lnc‐HZ04 and/or PKCA (Scale bar, 10 µm) and their quantification (*n* = 100 cells). Data were shown as means ± SD (standard deviation). *p* <0.05 meant significant differences compared with the control.

**Figure:**
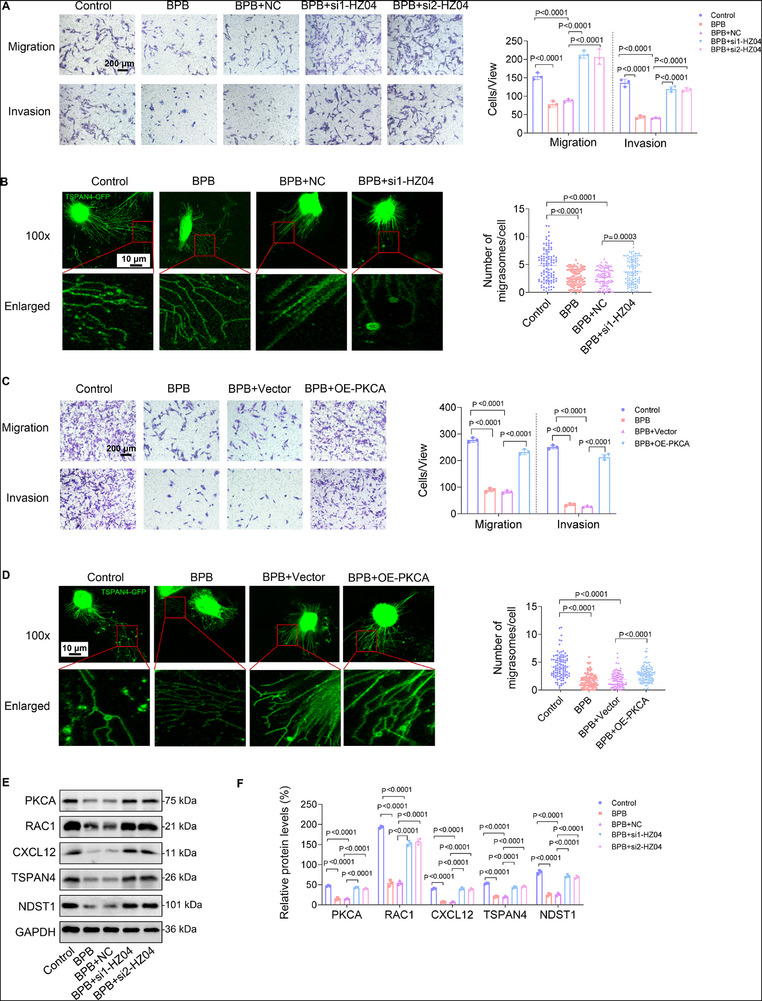

**Figure:**
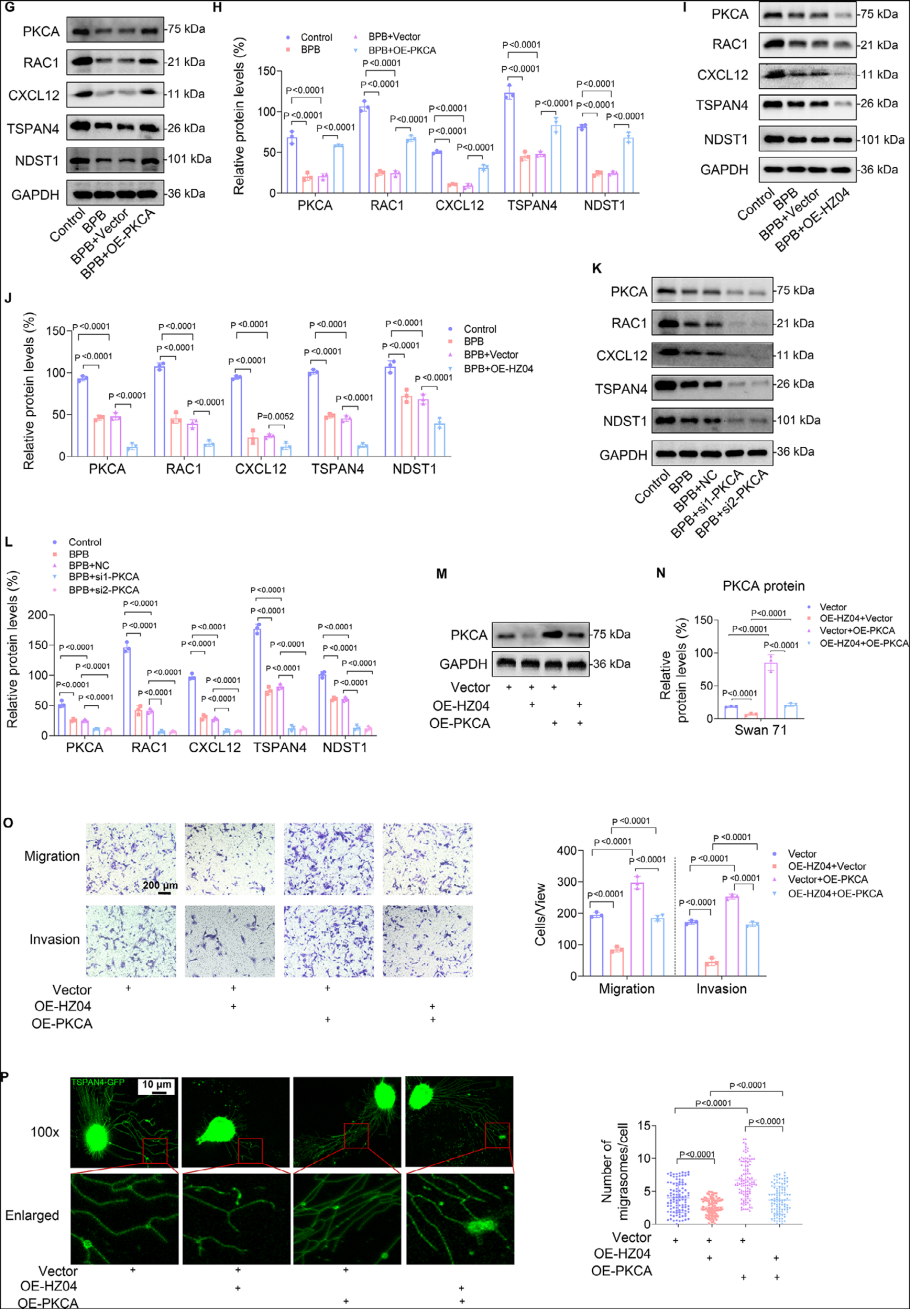

To further test the roles of PKCA in the functions of lnc‐HZ04‐overexpressed trophoblast cells, another set of co‐transfections assays were performed. First, lnc‐HZ04 overexpression down‐regulated the mRNA and protein levels of PKCA, a down‐regulation that could be abolished by co‐overexpressing PKCA in both Swan 71 and HTR‐8/SVneo cells (Figure 7M,N; Figure S9A,B, Supporting Information). The ability of lnc‐HZ04 to suppress MI and MF was also abolished by co‐overexpressing PKCA in human trophoblast cells (Figure 7O,P; Figure S9C,D, Supporting Information). Taken together, supplement with PKCA could efficiently recover MI and MF in lnc‐HZ04‐overexpressed trophoblast cells.

### 2.9 BPB Exposure Promoted ER‐Mediated lnc‐HZ04 Transcription

**Figure 8:**
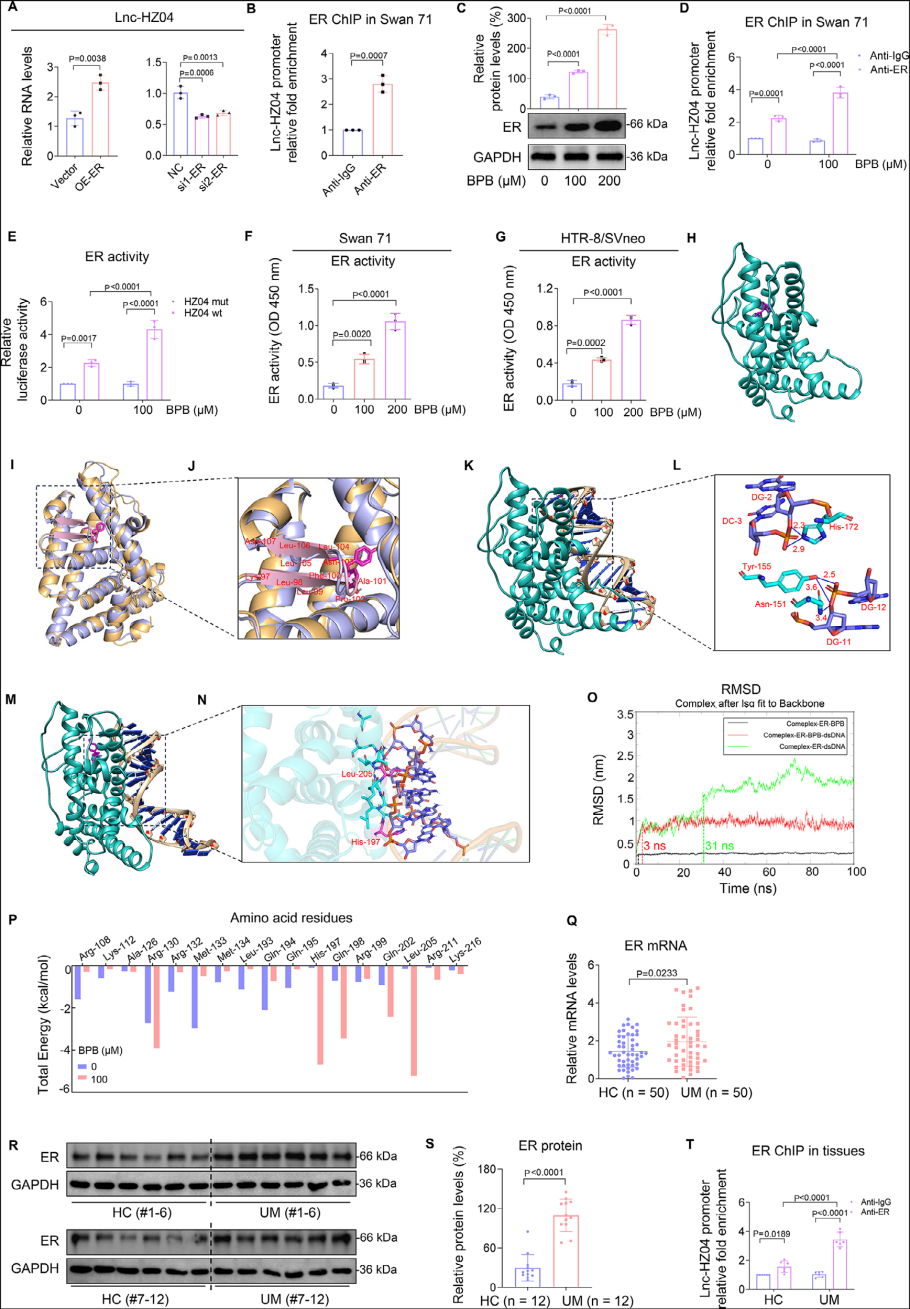
BPB exposure promoted ER‐mediated lnc‐HZ04 transcription. A) RT‐qPCR analysis of lnc‐HZ04 levels in Swan 71 cells with ER overexpression or knockdown. B) ER ChIP assay analysis of lnc‐HZ04 promoter region enriched by ER in Swan 71 cells. C) Western blot analysis of ER protein levels in BPB‐exposed Swan 71 cells and its relative quantification. D) ER ChIP assay analysis of the levels of lnc‐HZ04 promoter region enriched by ER in 100 µm BPB‐exposed Swan 71 cells. E) Dual‐luciferase reporter assay analysis of the transcription activity of ER using wild‐type (wt) or mutant (mut) promoter sequence of lnc‐HZ04 (sequences in Table S13, Supporting Information) in 100 µm BPB‐exposed Swan 71 cells. F,G) The levels of ER transcription activity in BPB‐exposed Swan 71 cells (F) and HTR‐8/SVneo (G). H) Molecular docking of BPB with ER using PLIP tool (Protein Ligand Interaction Profiler) and PyMOL v2.5.4. I,J) The conformational changes of the ER‐BPB complex relative to ER conformation and their enlarged region. K,L) Molecular docking of ER with dsDNA (promoter region) of lnc‐HZ04 using PyMOL v2.5.4 and their interactions such as H‐bond interactions (black dashed lines) with H‐bond lengths (Å) indicated by the numbers on the dashed lines. M,N) Molecular docking of BPB‐ER‐dsDNA complex structure and their enlarged interaction regions. His‐197 and Leu‐205 exhibited the greatest energy decreases in the presence of BPB. O) RMSD (Root‐mean‐square analysis) plots of the equilibrium status of ER‐BPB, ER‐dsDNA, and BPB‐ER‐dsDNA against stimulation time. P) The differences in binding energy between partial ER amino acid residues and dsDNA in the absence or presence of BPB. Q) RT‐qPCR analysis of ER mRNA levels in HC and UM villous tissues (each *n* = 50). R,S) Western blot analysis (R) of ER protein levels in HC and UM villous tissues and its relative quantification (S, each *n* = 12). T) ER ChIP assay analysis of the levels of lnc‐HZ04 promoter region enriched by ER in HC and UM villous tissues (each *n* = 6). Data were shown as means ± SD (standard deviation). *p* <0.05 meant significant differences compared with the control.

Subsequently, we explored how lnc‐HZ04 was up‐regulated in trophoblast cells. Predicted by PROMO software, ER might be a transcription factor of lnc‐HZ04. Experimentally, overexpression of ER up‐regulated lnc‐HZ04 expression levels, whereas knockdown of ER down‐regulated lnc‐HZ04 levels in both trophoblast cells (**Figure** **8**A; Figure S10A–E, Supporting Information). ChIP assays showed that ER could bind with the promoter region of lnc‐HZ04 (Figure 8B; Figure S10F, Supporting Information). All these results confirmed that ER was a transcription factor of lnc‐HZ04 and promoted lnc‐HZ04 transcription in human trophoblast cells.

It has been reported that BPB showed agonist activity of estrogen receptor (ER) in human breast cancer cells.^[^ ^68^ ^]^ However, its roles in human trophoblast cells were still unclear. First, BPB exposure up‐regulated lnc‐HZ04 expression levels in BPB‐exposed trophoblast cells (Figure 5B). Meanwhile, BPB also up‐regulated the protein levels of ER (Figure 8C; Figure S10G, Supporting Information), confirming that BPB up‐regulated the expression levels of ER in human trophoblast cells. Furthermore, ER ChIP assays showed that more promoter region of lnc‐HZ04 was enriched by ER in trophoblast cells after BPB exposure (Figure 8D; Figure S10H, Supporting Information). Dual‐luciferase reporter assays further showed that ER showed higher transcription activity using the wild‐type promoter region of lnc‐HZ04 after BPB exposure (Figure 8E; Figure S10I, Supporting Information). These results supported that BPB exposure promoted ER‐mediated lnc‐HZ04 transcription, explaining higher expression levels of lnc‐HZ04 in BPB‐exposed trophoblast cells.

To further dig the roles of BPB in ER‐mediated lnc‐HZ04 transcription, we investigated their potential structures based on molecular docking.^[^ ^69^, ^70^, ^71^ ^]^ First, we found that BPB could directly bind to ER and promote ER transcription activity using a trophoblast cell‐based ER transcription factor activation assay (Figure 8F,G. Molecular docking simulation gave the optimized free binding energy was −8.5 kcal mol^−1^ for BPB binding with ER, indicating that BPB could well bind with ER (Figure 8H; Figure S10J, Supporting Information).^[^ ^69^, ^70^, ^71^ ^]^ ER interacted with BPB through hydrophobic interactions, H‐bond interactions, and Pi‐Stacking (Figure S10K and Tables S4–S6, Supporting Information). Moreover, the bindings of BPB to ER also altered ER conformation, for example the random coil (or Ω loop) was transformed to regular β‐strand (residues 97–101 and 104–107) or β‐turn (residues 102–103) (Figure 8I,J). ER, as an lnc‐HZ04 transcription factor, could bind to the dsDNA promoter region of lnc‐HZ04 (sequences in Table S7, Supporting Information). Molecular docking showed that the docking score was −239.72 and confidence score was 0.8575, supporting their interactions (Figure 8K,L), which were further evidenced due to the presence of H‐bonds between His‐172 and DG‐2, His‐172 and DC‐3, Asn‐151 and DG‐12, Tyr‐155 and DG‐11, Tyr‐155 and DG‐11 (Figure 8K,L; Table S8, Supporting Information). BPB could also bind with ER‐dsDNA complex, giving the best binding energy of −8.5 kcal mol^−1^ (Figure 8M,N). RMSD (Root‐mean‐square analysis) curves showed that the ER‐BPB complex reached system equilibrium within 1 ns, the ER‐dsDNA complex reached equilibrium within 31 ns, and the ER‐BPB‐dsDNA complex reached equilibrium within 3 ns (Figure 8O), indicating that the presence of BPB increased the stability of the ER‐dsDNA complex. In details, the free binding energy of each amino acid residue of ER with dsDNA was altered in the presence of BPB (Figure 8P; Table S9, Supporting Information), reducing the total free binding energy from −38.63 kcal mol^−1^ for ER‐dsDNA complex to −64.35 kcal mol^−1^ for BPB‐ER‐dsDNA complex. The interactions of His‐197 and Leu‐205 in ER with dsDNA were significantly enhanced in the presence of BPB (Figure 8P). These results supported that BPB enhanced the interactions of ER with the lnc‐HZ04 promoter region. Collectively, these results demonstrated that BPB interacted with ER, which enhanced the binding of ER with the lnc‐HZ04 promoter region, explaining that BPB promoted ER‐mediated lnc‐HZ04 transcription in BPB‐exposed trophoblast cells.

In tissues, the mRNA and protein levels of ER were higher in UM vs HC tissues (Figure 8Q–S). ER ChIP assays showed that the levels of the promoter region of lnc‐HZ04 enriched by ER were higher in UM vs HC villous tissues (Figure 8T). These results showed that ER‐mediated lnc‐HZ04 transcription was enhanced in UM vs HC villous tissues.

### 2.10 Lnc‐HZ04 Levels in Serum were Associated with Miscarriage

**Figure 9:**
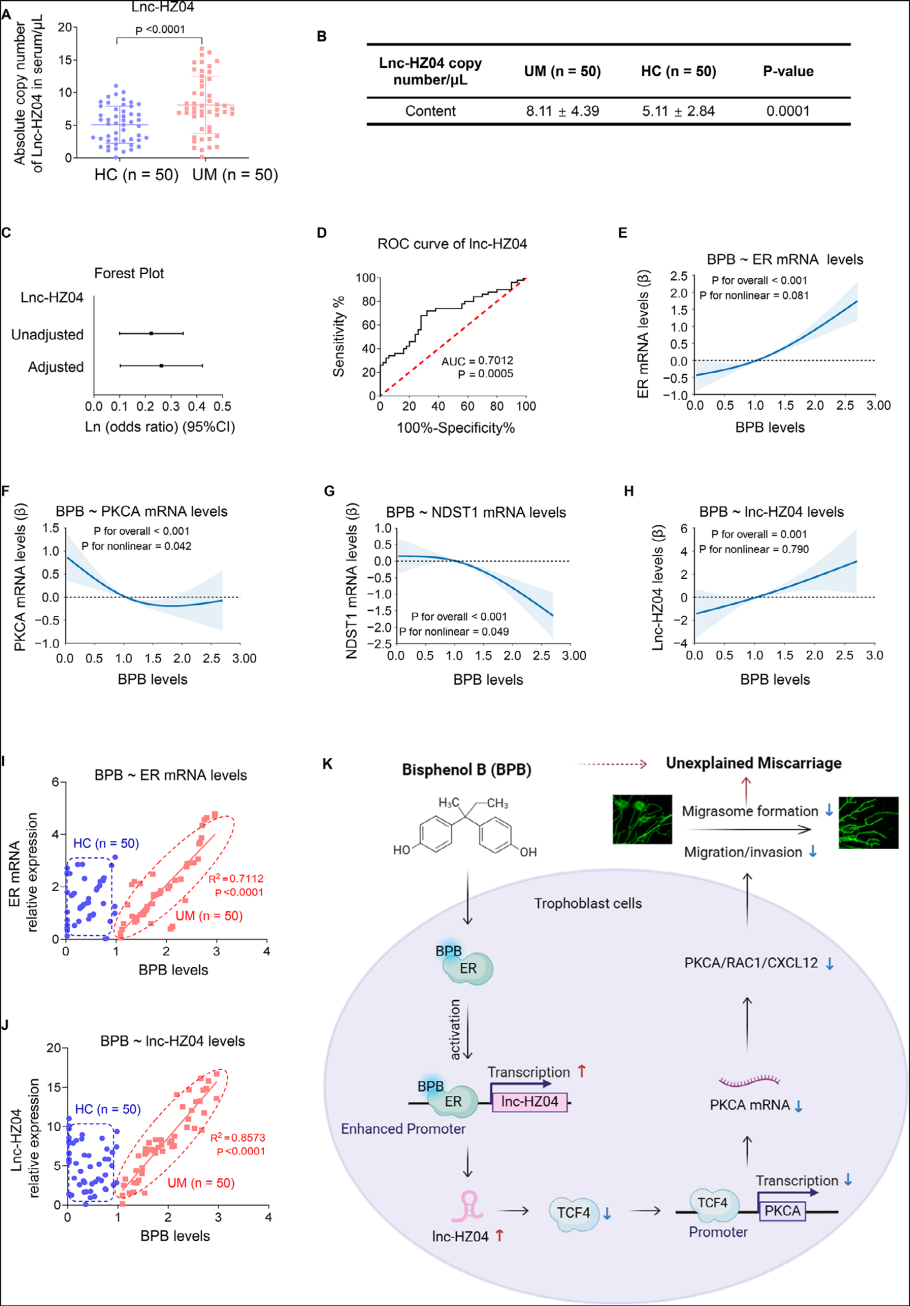
Serum lnc‐HZ04 levels and urinary BPB levels were associated with MI and MF. A) The absolute copy number of lnc‐HZ04 in HC and UM serum samples (each *n* = 50). B) Lnc‐HZ04 levels in HC and UM serum samples (each *n* = 50). C) Percent change (95% CI) levels of lnc‐HZ04 in serum. D) ROC curve analysis of lnc‐HZ04 in serum (*n* = 50). E–H) Restricted cubic spline (RCS) model analysis of the association between the levels of BPB in urine samples and the levels of ER mRNA (E), PKCA mRNA (F), or NDST1 mRNA (G) in villous tissues or lnc‐HZ04 levels (H) in serum in both HC and UM groups (each *n* = 50). The BPB levels were treated as the independent variable and the β‐transformed RNA levels as the dependent variable. The knots were calculated using the 5, 50, or 95th of their RNA levels, with the median values as the reference (horizontal line). I,J) Pearson correlation analysis of BPB levels in urine samples and ER mRNA (I) in villous tissues or lnc‐HZ04 levels (J) in serum in both HC and UM groups (each *n* = 50). K) The proposed mechanism. Data were shown as means ± SD (standard deviation). *p* <0.05 meant significant differences compared with the control.

Since lnc‐HZ04 was highly expressed in UM vs HC villous tissues, subsequently, we detected whether lnc‐HZ04 might also be higher in UM vs HC serum and whether lnc‐HZ04 in serum might be associated with miscarriage. For this aim, we detected the absolute copy number of lnc‐HZ04 in serum. The copies of lnc‐HZ04 were significantly higher in UM (8.11 ± 4.39 copies µL^−1^) relative to HC (5.11 ± 2.84 copies µL^−1^) serum samples (**Figure** **9**A,B). Multivariate logistic regression analysis by adjusting for all the confounders (Figure S1A, Supporting Information) showed that higher levels of lnc‐HZ04 in serum were associated with unexplained miscarriage (Figure 9C; Table S2, Supporting Information). Receiver Operating Characteristic (ROC) curve analysis also showed that the levels of lnc‐HZ04 in serum might have predicative capability for miscarriage (Figure 9D). Taken together, the data indicated that lnc‐HZ04 in serum might be associated with miscarriage.

### 2.11 Urinary BPB Levels were Associated with MI and MF

The associations between the levels BPB in urine samples and the RNA levels of ER, lnc‐HZ04, PKCA, or NDST1 in villous tissues in both HC and UM groups were analyzed. Restricted cubic spline (RCS) model analysis showed that they were all associated (all P <0.05, Figure 9E–H). To further distinguish non‐linear or linear association, restricted cubic spline (RCS) model analysis showed that the levels of urinary BPB were non‐linearly associated with the mRNA levels of PKCA, and NDST1 in UM villous tissues (P for non‐linear <0.05, Figure 9G,H) and were linearly associated with the levels of ER mRNA (Figure 9E) and lnc‐HZ04 (Figure 9F) in UM villous tissues (P for non‐linear > 0.05). Pearson correlation analysis also showed that BPB exposure was positively associated with ER mRNA levels (Figure 9I) lnc‐HZ04 expression levels in UM villous tissues (Figure 9J). Taken together, all the results indicated that BPB levels in urine were associated with MI, MF, and miscarriage.

## 3 Discussion

BPB, a substitute of BPA, belongs to endocrine disrupting chemicals. It has been reported that BPB exposure alters reproductive functions of rats and fish^[^ ^72^ ^]^ and induces oocytes, spermatogonia, spermatocytes, and spermatids dysfunctions.^[^ ^73^, ^74^ ^]^ However, whether BPB might be associated with miscarriage is still largely unknown. In this study, BPB was detected out from urine samples in both UM and HC groups. BPB concentrations are significantly higher in UM vs HC urine samples and are associated with the occurrence of unexplained miscarriage. Mouse model assays directly give evidence that BPB exposure induces mouse miscarriage. Subsequently, cellular assays, tissue assays, and animal model assays further show that BPB exposure suppresses MI and MF, discovering BPB novel cytotoxicity effects and the novel pathogenesis of unexplained miscarriage.

Migrasome is a newly identified cellular organelle and governs critical cellular processes such as mitochondrial quality control,^[^ ^35^ ^]^ cell–cell communication,^[^ ^36^ ^]^ and lateral transfer of mRNAs and proteins.^[^ ^37^ ^]^ However, whether migrasome formation is associated with miscarriage is largely unknown. Moreover, whether BPB exposure might affect migrasome formation is completely unknown. In this study, it is the first time that we correlate BPB exposure, migrasome formation, and miscarriage. We find that BPB exposure suppresses MI and MF, which is closely associated with miscarriage. Moreover, we also find that lnc‐HZ04 regulates migrasome formation by down‐regulating PKCA/RAC1/CXCL12 pathway. As far as we known, it is the first example that an lncRNA regulates migrasome formation.

The regulatory mechanisms of BPB‐suppressed MI and MF are proposed (Figure 9K). BPB exposure up‐regulates ER expression levels, which further promotes ER‐mediated lnc‐HZ04 transcription and thus up‐regulates lnc‐HZ04 expression levels. Subsequently, lnc‐HZ04 down‐regulates TCF4 expression levels and suppresses TCF4‐mediated PKCA transcription. Therefore, lnc‐HZ04 down‐regulates PKCA expression levels and suppresses the PKCA/RAC1/CXCL12 pathway, which further suppresses MI and MF in human trophoblast cells, possibly inducing miscarriage. Knockdown of lnc‐HZ04 or supplement with PKCA could efficiently alleviate BPB‐suppressed MI and MF in trophoblast cells. In the mouse model, supplement with murine Pkca efficiently recovers the murine Pkca/Rac1/Cxcl12 pathway, recovers MI and MF in murine placental tissues, and alleviates mouse miscarriage, providing a potential target for therapy of BPB exposure‐induced miscarriage. The PKCA/RAC1/CXCL12 pathway is suppressed in BPB‐exposed human trophoblast cells, in UM vs HC villous tissues, and in the placental tissues of BPB‐exposed mice. Lnc‐HZ04 is highly expressed in BPB‐exposed human trophoblast cells and in UM vs HC villous tissues. Lnc‐HZ04 levels in serums are associated with miscarriage. Lnc‐HZ04 homologue is not identified in the mouse system, indicating the complicated and specific epigenetic regulatory mechanisms in humans.

It has been reported that several lncRNAs regulate trophoblast cell functions and the occurrence of miscarriage, such as lncRNA TUG1,^[^ ^75^ ^]^ PVT1,^[^ ^76^ ^]^ and LINC00240.^[^ ^77^ ^]^ In our recent studies, we have identified a group of novel lncRNAs, such as lnc‐HZ01, lnc‐HZ03, lnc‐HZ04, lnc‐HZ05, lnc‐HZ06, lnc‐HZ08, lnc‐HZ09, lnc‐HZ10, lnc‐HZ11, lnc‐HZ12, and lnc‐HZ14, all of which regulated the dysfunctions of human trophoblast cells and the occurrence of miscarriage.^[^ ^27^, ^30^, ^49^, ^50^, ^51^, ^52^, ^53^, ^54^, ^55^, ^56^, ^57^, ^58^ ^]^ Among them, lnc‐HZ04, which is highly expressed in BPDE‐exposed trophoblast cells and in UM vs HC villous tissues, promotes trophoblast cell apoptosis and miscarriage. In this study, we find that BPB exposure also up‐regulates lnc‐HZ04 expression levels, which suppresses MI and MF in BPB‐exposed trophoblast cells and induces miscarriage. These results suggest that different environmental toxicants (such as BPDE and BPB) might up‐regulate the expression levels of an identical lncRNA (such as lnc‐HZ04), which subsequently causes different dysfunctions of human trophoblast cells and then induces miscarriage. These results also demonstrate that epigenetic molecules might act as important hub molecules to connect between environmental toxicant exposure and trophoblast cell dysfunctions.

Although increasing studies have shown that exposure to environmental toxicants alters the expression profiles of mRNAs and non‐coding RNAs,^[^ ^78^ ^]^ their underlying mechanisms are rarely explored. In this work, we find that exposure to BPB up‐regulates the expression levels of ER in human trophoblast cells. ER could act as a transcription factor to promote lnc‐HZ04 transcription. Notably, BPB could also bind with ER and alters ER conformation, which enhances the binding of ER with the promoter region of lnc‐HZ04, promoting lnc‐HZ04 transcription. On a larger scale, BPB exposure might regulate ER‐mediated transcription of a vast number of RNAs (including non‐coding RNAs) to alter the expression profiles of mRNAs and non‐coding RNAs in BPB‐exposed human cells.

Although some studies have shown extensive regulatory mechanisms of miscarriage,^[^ ^79^, ^80^ ^]^ miscarriage therapy strategy has been less reported. Protein kinase C alpha (PKCA) is involved in the regulation of various cellular biological processes, such as proliferation, invasion/migration, apoptosis, and cell cycle.^[^ ^40^ ^]^ However, whether PKCA might be used for miscarriage treatment is completely unexplored. In this study, we construct a miscarriage intervention model and find that supplement with murine Pkca could efficiently recover the PKCA/RAC1/CXCL12 pathway, restore invasion/migration and migrasome formation, and alleviate miscarriage. Moreover, supplement with murine Tspan4 could also restore migrasome formation and alleviate miscarriage. These results not only validate the pathogenesis of BPB‐induced miscarriage but also provide potential targets for treatment against unexplained miscarriage.

The results in this case‐control study were consistent with those in mouse model assays and cellular assays. Nonetheless, the case‐control study has several limitations. First, although we consider some confounders in this study, other confounders might be ignored. Second, although we classify some confounders based on the literature, the possibility of misclassifications might not be completely avoided. Third, some information is collected by self‐report, some factors (e.g., recall bias) might affect the data reliability.

In this work, because the lnc‐HZ04 homologue is not identified in the mouse system,^[^ ^30^ ^]^ only the Pkca/Rac1/Cxcl12 pathway is explored in the mouse model. Miscarriage treatment with lnc‐HZ04 might be explored using other animal model, such as swine or the rhesus model. In addition to the PKCA/RAC1/CXCL12 pathway, we have also analyzed another two migration/invasion‐related pathways (such as ERK, Wnt/β‐catenin), which are significantly altered in the sequencing data (Figure 2B). Further assays also confirmed that BPB exposure down‐regulates ERK1 and β‐Catenin protein levels (Figure S11A,B, Supporting Information). Therefore, BPB exposure might suppress invasion/migration possibly through other signaling pathways, which should be further explored. Although BPB levels are higher in the UM vs HC group and BPB exposure is associated with miscarriage, it cannot exclude that other toxicants (such as other bisphenol analogues, BPA, BPS, BPF, etc.) might also be associated with miscarriage or directly induce miscarriage. Although we find that BPB levels were higher in UM vs HC urine samples (*n* = 100), we used a single‐spot of urine samples, which might have potential misclassification bias if the BPB levels vary greatly over time due to changed external exposures, daily activities, or physiological status. In this study, we used the REED or higher doses of BPB in animal and cell assays. In fact, there is the possibility that women might ingest excessive BPB in areas with high BPB pollution, with BPB occupational exposure or occupational poisoning, or with possible long‐term cumulative exposure. To a certain extent, the short‐term and high‐dose BPB‐exposed animal and cell models might reflect or predict the long‐term and low‐dose effects of BPB exposure on women miscarriage. Tetraspanin 4 (TSPAN4) belongs to the tetraspanin family and is required for the formation of migrasomes.^[^ ^81^ ^]^ In addition to these, TSPAN4 might also assemble around the damage site of the membrane to facilitate membrane repair.^[^ ^82^ ^]^ Since migrasome is an emerging research hotspot, more novel, specific, and direct approaches are still required to directly visualize and examine migrasome functions in vivo. The lack of direct rescue experimental validation using migrasomes provides a possibility that supplement with murine Tspan4 alleviates mouse miscarriage through other functions of Tspan4 rather than migrasomes. To use the swine or rhesus model and further explore the environment and female pregnant healthy are very promising. Finally, this study also warns pregnant women to avoid exposure to BPB or its analogues as much as possible.

In summary, we find that urinary BPB levels are higher in UM vs the HC group and are associated with unexplained miscarriage. In the mouse model, BPB exposure indeed induces mouse miscarriage. In mechanism, BPB exposure up‐regulates lnc‐HZ04 levels, suppresses PKCA/RAC1/CXCL12 pathway, and further suppresses migration/invasion and migrasome formation in human trophoblast cells, possibly inducing miscarriage. Knockdown of lnc‐HZ04 or supplement with PKCA could efficiently alleviate BPB‐suppressed MI and MF in trophoblast cells. In the mouse model, supplement with murine Pkca efficiently recovers the murine Pkca/Rac1/Cxcl12 pathway, restores MI and MF in murine placental tissues, and alleviates mouse miscarriage. Supplement with murine Tspan4 could also restores MF and alleviates mouse miscarriage. These results not only discover novel toxicological effects of BPB exposure on human reproductive health but also provides potential targets for therapy of unexplained miscarriage.

## 4 Experimental Section

### Chemicals and Reagents

BPB (purity, 98%) was from Solarbio (B6500, Beijing, China). Anhydrous DMSO was from Sigma–Aldrich (St. Louis, MO, USA). BPB was dissolved in DMSO at 200 mm and stored at −80 °C. All the vehicle control and the treated cultures contained the identical amount of DMSO (0.03%, v/v).

### Tissues Collection and Statement

The100 patients with unexplained miscarriage (UM group) and 100 women who sought induced miscarriage to terminate their unwanted pregnancies were newly recruited, which were considered as the healthy control (HC) group, in the age between 25 and 30, and treated at the Eighth Affiliated Hospital, Sun Yat‐sen University from May 2024 to December 2024, as the methods described previously.^[^ ^54^, ^59^ ^]^ Any women with clinically known causes of miscarriage were excluded based on the inclusion and exclusion criteria, such as cervical incompetence, chromosome abnormalities, endocrine or metabolic diseases, virus or bacterial infections, as the method described previously.^[^ ^54^, ^59^ ^]^ HC and UM women did not receive any treatment. The characteristics of these HC and UM women were listed in Table S1 (Supporting Information), including baseline characteristics (age, body mass index, education, residence, household income), clinical information (gravidity, gestational weeks, RBC, WBC, Hb), and lifestyle (smoking, drinking, taking canned food and bottled drinks). Some characteristics, including age, BMI, gravidity, gestational weeks, RBC, WBC, Hb, smoking, and drinking, were collected from medical records, and information was recorded on the first day of their visit. Chinese cut‐off points were used to define underweight (<18.5 kg m^−2^), normal (18.5–23.9 kg m^−2^), overweight (24–27.9 kg m^−2^), and obese (≥ 28 kg m^−2^). Other characteristics, including age, BMI, gravidity, gestational weeks, smoking, drinking, education, residence, household income, taking canned food and bottled drinks, were collected by well‐trained interviewers using standard questionnaires (see Supplementary Questionnaire) on the day of the miscarriage operation. The intersected information were consistent. A piece of villous tissue with dimensions of approximate 2 × 0.5 × 0.5 cm^3^ was manually dissected from the fetal side of the placenta and was cleared of maternal decidua from the two groups of women at 6–8 weeks of gestation (each *n* = 50). These samples were serially washed and immediately frozen in liquid nitrogen and stored at −80 °C until further use. For RNA or protein extraction, ≈30 mg villous tissues were homogenized in 600 µL Trizol reagent (Invitrogen) for RNA extraction or in 600 µL RIPA lysis buffer (Thermo Fisher Scientific) containing protease inhibitor cocktail for protein extraction using Silica beads (107735, Merck, Darmstadt, Germany) via shaking for a 1‐min burst using a TissueLyser LT instrument (Qiagen). A single‐spot of midstream urine sample^[^ ^83^, ^84^, ^85^, ^86^, ^87^, ^88^, ^89^, ^90^ ^]^ was collected from these women using polypropylene containers^[^ ^23^, ^60^ ^]^ on the same day before the miscarriage operation (each *n* = 100). The protocols were approved by the Ethics Committee of the Eighth Affiliated Hospital, Sun Yat‐sen University. Written informed consent was obtained from all the patients before performing the study.

### LC‐MS/MS Analysis of BPB in Urine Samples

Clean midstream urine samples were collected from HC and UM women (each *n* = 100) using polypropylene containers.^[^ ^23^, ^60^ ^]^ The urine storage, processing, and analysis were performed using glass instruments^[^ ^91^ ^]^ or tubes to avoid potential BPB contamination. All the urine samples were stored at −20 °C before analysis. In brief, a 500‐µL urine sample in a glass bottle was mixed with 10 µL of β‐glucuronidase/sulfatase solution (>110 000 units), 50 µL isotope internal standards, and 200 µL ammonium acetate buffer (pH = 5.0, 1 mol L^−1^). After incubation at 37 °C for 24 h, the mixture was extracted thrice with 3 mL methyl‐tert‐butyl ether/ethyl acetate. After that, the extract was evaporated with nitrogen and re‐dissolved in150 µL water/acetonitrile. BPB was determined as similar methods described previously.^[^ ^61^ ^]^ The bisphenols in the samples were separated by a UHPLC system Ultimate 3000 equipped with a 1.7 µm, 100 × 2.1 mm Acquity UPLC BEH C18 analytical column (Waters, Milford, MA, USA) with water/acetonitrile and were analyzed by an AB Sciex 6500 triple quadrupole mass spectrometry (MS/MS, Toronto, Canada) in negative mode. The intra‐ and inter‐batch precision were below 6.3% and 1.7%, respectively. The quality control samples using pooled urine samples spiked with the target chemicals and a blank sample were analyzed along with each batch. The limit of detection (LOD) of BPB in urine samples was 0.04 µg L^−1^. BPB concentrations in reagent blanks were all below the LODs. BPB had detection rates >86.5% in all urine samples. The levels of BPB lower than LOQ were assigned as LOD/√2 (0.028 µg L^−1^) for graphical representations and data analysis. The spiked recoveries ranged from 85.3% to 97.6%, and the relative standard deviations were below 10.0%.

### Cell Culture

Human trophoblast Swan 71 cells were immortalized by human telomerase, constructed by Gil Mor's group at Yale University. HTR‐8/SVneo cells, human first‐trimester trophoblast cells that had been immortalized by SVneo virus, were purchased from Hunan Fenghui Biotechnology Co., Ltd (CL0164, Changsha, Hunan, China). Mouse placental chorionic trophoblast cells were purchased from Procell Life Science & Technology Co., Ltd (CP‐M144, Hubei, China). Swan 71 cells were cultured in DMEM medium (Gibco, C11995500BT). HTR‐8/SVneo cells were cultured in RPMI 1640 medium (Gibco, C11875500BT). Mouse trophoblast cells were cultured in DMEM/F12 medium (Gibco, C11330500BT). All media were supplemented with 10% fetal bovine serum (FBS, Gibco, 1347575). Cells were cultured at 37 °C in a 5% CO_2_ incubator.

To explore the effects of real environmental exposure dose (REED) of BPB on miscarriage, the actual daily intake dose of total bisphenols in the human body was calculated. One study has shown that total bisphenol analogue levels were 144 ng mL^−1^ in the serum of pregnant women in Beijing, China.^[^ ^22^ ^]^ This dose could be considered as a representative of the human real intake dose under the inadvertent environment. According to BPB molecular weight of 242 g mol^−1^, this REED of BPB corresponded to 0.6 µm BPB in cells. It had been reported that exposure of mouse oocyte to BPB (100, 150, or 200 µm) could compromise meiotic maturation and impair oocyte quality.^[^ ^20^ ^]^ The half‐maximal inhibitory concentration (IC50) of BPB for blocking the androgen receptor was 100 µm.^[^ ^92^ ^]^ Based on the doses used in literature and pre‐experiments, in this study, it was selected that 0 (control), 0.6 µm (1‐fold REED), 6 µm (10‐fold REED), 100 µm (167‐fold REED), or 200 µm (333‐fold REED) BPB to construct the BPB‐exposed human trophoblast cell models.

### Cell Transfection

Plasmids for overexpression of lnc‐HZ04 (pcDNA3.1‐HZ04), PKCA mRNA (pcDNA3.1‐PKCA), murine Pkca mRNA (pcDNA3.1‐Pkca), murine Tspan4 mRNA (pcDNA3.1‐Tspan4), TCF4 mRNA (pcDNA3.1‐TCF4), or ER mRNA (pcDNA3.1‐ER) were synthesized by Addgene (Watertown, MA, USA, Table S10, Supporting Information). The corresponding RNA sequences were obtained from the NCBI database: Gene Bank 2013. Empty pcDNA3.1 was used as a negative control (Vector). Si‐HZ04, si‐PKCA, si‐TCF4, si‐ER, and si‐NC (negative control) were designed and synthesized by Thermo Fisher (Catalog number: 4392420, Table S11, Supporting Information). Swan 71 and HTR‐8/SVneo cells (1 × 10^6^ cells well^−1^) were seeded in 6‐well plates and cultured to 80% confluence. Transfection was performed in Lipofectamine 3000 (Invitrogen, Carlsbad, CA, USA) medium for 24 h according to the manufacturer's protocols. The transfection efficiencies were validated by RT‐qPCR.

### Cell Migration/Invasion

Trophoblastic cells (1 × 10^6^ cells well^−1^) with the identical passage in six‐well plates were transfected with 50 nm si‐NC, si‐HZ04, si‐PKCA, si‐TCF4, or si‐ER in Lipofectamine 3000 media for 24 h. Cells were also transfected with 2.0 µg well^−1^ pcDNA3.1, pcDNA3.1‐HZ04, pcDNA3.1‐PKCA, pcDNA3.1‐TCF4 or pcDNA3.1‐ER in Lipofectamine 3000 for 24 h. The surviving cells were re‐suspended into DMEM medium or RPMI 1640 medium after trypsin digestion. For migration assays, 1 × 10^4^ cells well^−1^ were seeded in 24‐well transwell chambers (3422, Corning, Lowell, MA, USA) and cultured for 24 h. Cells were quantified by the TC20 Automated Cell Counter (Bio‐Rad Laboratories, Hercules, CA, USA). For invasion assays, 80 µL aliquots of matrigel (BD Biosciences, Franklin Lakes, NJ, USA) diluted in DMEM medium or RPMI 1640 medium (dilution ratio 1:30) were coated on 24‐well transwell chambers and were solidified at 37 °C for 1 h. Cells (1 × 10^4^ cells well^−1^) were plated on top of the matrigel matrix and cultured for 24 h. The bottom chamber contained medium with 10% FBS as a chemoattractant of human trophoblast cells. After 24 h, the whole chambers were fixed with 4% paraformaldehyde for 20 min, stained with crystal violet for 15 min, and then washed thrice with PBS. For visualization, the cells on the bottom surface of the membrane were photographed by Axio observer3 (Zeiss, Germany) at 200 × magnification and counted in five random fields.

### Quantitative Real‐Time Polymerase Chain Reaction (RT‐qPCR)

Total RNAs were extracted from Swan 71 cells, HTR‐8/SVneo cells, or human villous tissues using TRIzol reagent (Invitrogen, 15596026). RNA quality and quantity were assessed using a NanoDrop 2000 UV spectrophotometer (Thermo Fisher Scientific) and RNA integrity number (RIN) were assessed on an Agilent 2100 Bioanalyzer (Agilent Technologies, Palo Alto, CA, USA). The concentration of RNA was between 500–1500 ng µL^−1^ and the RIN ≥ 9. The RNA purity was high as the A260/280 values were in the range of 1.8–2.2. Briefly, the isolated RNAs (800 ng) were converted into cDNAs using the first‐strand cDNA synthesis kit (Invitrogen) using the first strand cDNA synthesis kit (Invitrogen). Quantitative real‐time PCR was performed using an iQ5 real‐time detection system (Bio‐Rad Laboratories) and SYBRVR Premix Ex TaqTM II (Bio‐Rad Laboratories). The expression levels of RNAs were analysed using the 2^−ΔΔCt^ method.^[^ ^93^, ^94^ ^]^ GAPDH mRNA was used as the normalization internal control for lncRNAs and mRNAs. The primer sequences are listed in Table S12 (Supporting Information).

### Western Blot (WB) Analysis

Cells or tissues were lysed by RIPA lysis buffer (Thermo Fisher Scientific) and quantified using Pierce BCA Protein Assay Kit (Pierce, Rockford, IL, USA), then boiled for 10 min at 95 °C. Proteins (10–30 µg well^−1^, equal amounts within group but different amounts among groups for better comparison) were separated on 10% sodium dodecyl sulfate‐polyacrylamide gel electrophoresis (SDS‐PAGE), followed by electrophoretic transfer onto PVDF membranes. Membranes were blocked with 5% non‐fat milk in TBST buffer, and incubated with primary antibody overnight at 4 °C. Membranes were incubated with HRP‐conjugated secondary antibody (Abcam, Cambridge, UK) for 1 h at room temperature, and protein bands were detected with an enhanced chemiluminescence (Thermo Fisher Scientific). The following primary antibodies were used for Western blot analysis at the indicated dilution: anti‐PKCA (dilution 1:1000, ab32376, Abcam), anti‐RAC1 (dilution 1:2000, ab180683, Abcam), anti‐CXCL12 (dilution 1:5000, ab155090, Abcam), anti‐TCF4 (dilution 1:10000, ab217668, Abcam), anti‐ER (dilution 1:5000, ab32063, Abcam), anti‐TSPAN4 (dilution 1:1000, PA5‐69344, Invitrogen), anti‐NDST1 (dilution 1:500, sc‐100790, Santa Cruz Biotechnology) and anti‐GAPDH (dilution 1:10000, ab8245, Abcam). Protein band intensity was quantified with ImageJ. All experiments were replicated thrice. For better comparison of the protein bands in control and experiment groups, different amount of cell lysates was loaded in different experiments. The intensity of each band was quantified by Image J. The value of each band density in experimental and control groups was normalized to that of its corresponding GAPDH band (loading control, ratio to GAPDH%).

### Confocal Imaging and Image Analysis

For live‐cell imaging, 35‐mm glass‐bottom dishes (MatTek Corp.) were coated with 10 µg mL^−1^ fibronectin (PHE0023, Gibco) in PBS for ≥3 h at 37 °C and then washed with PBS twice. Swan 71 cells were cultured in fibronectin‐precoated confocal dishes for 12 h and then transfected with TSPAN4‐GFP‐expressing plasmid for 12 h. Confocal snapshot images were acquired using a Zeiss laser scanning confocal micro‐scope (LSM880) at 1024 × 1024 pixels. Images were processed with ImageJ, and statistical analysis was conducted using Graphpad Prism 8.

### ChIP (Chromatin Immunoprecipitation) Assay

Swan 71 or HTR‐8/SVneo cells (1 × 10^7^) were cross‐linked using 1% formaldehyde for 10 min at room temperature and then treated with 250 mm glycine for 5 min to quench reactions. Cells were lysed in cell lysis/wash buffer (150 mm NaCl, 5 mm EDTA [pH=7.5], 50 mm Tris‐HCl [pH 7.5], 0.5% NP‐40) containing protease inhibitor for 10 min on ice and then sonicated to generate DNA fragmentation with length of 200–500 bp in shearing buffer (1% SDS, 10 mM EDTA [pH=8.0], 50 mm Tris‐HCl [pH=8.0]) containing protease inhibitor using a Diagenode BioruptorPlus sonicator (30 s on and 30 s off for 12 cycles). Then, DNA fragmentations were incubated with anti‐TCF4 (dilution 1:100, ab217668, Abcam) or IgG control (Invitrogen, 02‐6102) overnight at 4 °C and then incubated with Dynabeads Protein G beads at 4 °C for 4 h on a rotating wheel. Subsequently, beads were washed for six times with cell lysis/wash buffer at 4 °C and then washed twice with cold TE buffer (Invitrogen, 12090015). Then, the crosslinking was terminated by treating cells in elution buffer (100 mm NaHCO_3_ and 1% SDS) on a shaker at room temperature for 15 min. Then, the crosslink of antibody‐bound chromatin complexes were dissociated at 65 °C with 5 m NaCl overnight to release free DNA. Subsequently, each sample was treated with 50 ng µL^−1^ RNase A at 37 °C for 30 min and then with 10 mm Proteinase K at 45 °C for 1 h. Subsequently, the immunoprecipitated DNA was extracted with the Min‐Elute PCR purification kit (Qiagen, 28004) and was analyzed by qPCR using the specific primers (Table S12, Supporting Information). First, ESR1 binding sites in the lnc‐HZ04 promoter region (5′‐GGCATGAGCCACCCTGCCCA‐3′) was predicted using the Animal Transcription Factor Database version 4.0 (https://guolab.wchscu.cn/AnimalTFDB4/#/). This promoter region has high homology to the canonical estrogen responsive element. Subsequently, this binding site was further validated in ChIP assays using specifically designed primers targeting the predicted regions.

### Luciferase Reporter Assay

Luciferase reporter assays were performed as described previously.^[^ ^30^, ^56^ ^]^ Briefly, wild‐type (wt, 5′‐CCCACCTGCG‐3′) or mutant (mut, 5′‐GGGTGGACGC‐3′) sequence in PKCA promoter region or wt (5′‐GGCATGAGCCACCCTGCCCA‐3′) or mut (5′‐CCGTACTCGGTGGGACGGGT‐3′) sequence in lnc‐HZ04 promoter region was fused into the luciferase pGL3‐basic reporter vector (Promega, Madison, USA) to construct pmirGLO‐PKCA‐wt/‐mut or pmirGLO‐HZ04‐wt/‐mut (sequences in Table S13, Supporting Information). Swan 71 or HTR‐8/SVneo cells were seeded into 24‐well plates and were co‐transfected with 100 ng pmirGLO‐PKCA‐wt/‐mut or pmirGLO‐HZ04‐wt/‐mut and 100 nm si‐HZ04 in Lipofectamine 3000 Transfection Reagent (Invitrogen) or exposed to 100 µm BPB for 48 h according to the manufacturer's instructions. Cells were lysed using passive lysis buffer (Promega Corporation) and the firefly luciferase activity in each well was measured using Dual‐Luciferase Reporter Assay System (Promega) according to the manufacturer's protocols.

### ER Transcription Factor Activation Assay

Swan 71 or HTR‐8/SVneo cells (3 × 10^5^/well) were incubated in DMEM medium without phenol red and containing 0, 100, or 200 µm BPB for 24 h. Then, the nucleus was extracted using the NE‐PER nuclear and cytoplasmic extraction reagents kit (78833, Thermo Scientific) according to the manufacturer's instructions. ER transcription factor activation assay was determined using ER receptor transcription factor assay kit (ab207203, Abcam, Cambridge, UK) according to the manufacturer's instruction. The nuclear samples were sonicated for four times (sonication for 5 s and break for 10 s for each cycle) on ice. The protein concentration was measured with NanoDrop. Then, the nuclear extracts (10 µg) were added to each well immobilized with ER binding site dsDNA (5′‐GGTCACAGTGACC‐3′). After wash with PBS, rabbit anti‐ERα (1:2000) and HRP‐conjugated secondary antibody (1:2000) were added to these wells. The ER transcription activity was calculated by recording the absorbance of samples at 450 nm using the Infinite M2000 Pro plate reader (Tecan, Crailsheim, Germany). DMSO‐treated cells were used as the control group.

### Immunohistochemistry Assays (IHC)

Human Villous tissue and murine placental tissues samples were fixed with 4% paraformaldehyde and embedded in paraffin. Sections (4 µm) were de‐waxed, rehydrated, and incubated in 0.01 m citrate buffer for 20 min at 95 °C for antigen retrieval. Endogenous peroxidase activity was blocked with 3% hydrogen peroxide (Cat. No. HX0636, Millipore, Billerica, MA, USA), and non‐specific antigens were blocked with 10% normal goat serum (Cat. No. 16210072, Gibco, USA), followed by incubation with primary antibody at 4 °C for 12 h. The primary antibodies included anti‐rabbit PKCA (1:200, abcam), anti‐rabbit TSPAN4 (1:600, Invitrogen), and anti‐mouse NDST1 (1:500, Santa Cruz Biotechnology). Sections were rinsed with PBS and incubated with goat anti‐rabbit secondary antibody (Abcam, ab6721, 1:5000, UK) and goat anti‐mouse secondary antibody (Abcam, ab205719, 1:3000). The antigens were visualized using 3,3′‐diaminobenzidine (Cat. No. D8001, Millipore, Billerica, MA, USA), and the slides were counter‐stained with hematoxylin (Cat. No. H9627, Millipore, Billerica, MA, USA) at 25 °C for 2 min.

### Wheat‐Germ Agglutinin (WGA) Staining

Villous tissues and mouse placental tissues were frozen using a freezing microtome (CRYOSTAR NX50, Thermo Fisher) in optimal cutting temperature compound (4583, Solarbio) and were cut into multiple 10‐µm sections sequentially. The tissue sections were transferred into adhesive glass slides (188105, CITOTEST, Jiangsu, China) and were thawed in PBS at room temperature. The specimen was stained with WGA (1 µg mL^−1^, W11261, Invitrogen) at 37 °C for 10 min and then washed with PBS. Confocal snapshot images were acquired using a Zeiss laser scanning confocal micro‐scope (LSM880) at 1024 × 1024 pixels.

### Immunofluorescence (IF) Staining

Mouse placental tissues were fixed with 4% formaldehyde in PBS and permeabilized with 0.1% Triton X‐100. After washing with PBS thrice, the tissues were blocked with 5% bovine serum albumin for 1 h at room temperature followed by incubations with primary antibodies at 4 °C overnight. The primary antibodies included anti‐rabbit PKCA (1:200, abcam) and KRT7 (green; Abcam, ab181598; 1:500). KRT7 could be considered as a specific biomarker of mouse placental trophoblast cells in the placenta.^[^ ^67^ ^]^ Then, the tissues were washed thrice with PBS and then incubated with secondary antibodies for 1 h at room temperature. The secondary antibodies of PKCA and KRT7 were Goat Anti‐Rabbit IgG H&L (Red, Alexa Fluor 594) and Goat Anti‐Rabbit IgG H&L (Green, Alexa Fluor 488), respectively. Afterward, the tissues were further incubated with 4′,6‐diamidino‐2‐phenylindole (100 ng mL^−1^) for 10 min. Cell nuclei were stained with DAPI (blue, Invitrogen). Images were captured using a Zeiss laser scanning confocal micro‐scope (LSM880).

### High‐Throughput mRNA Sequencing and Data Processing

Swan 71 cells (5 × 10^6^ cells) were exposed with 100 µm BPB for 24 h and were used for high‐throughput transcriptome sequencing. Villous tissues (30 mg) from UM and HC groups were collected for high‐throughput mRNA sequencing. Total RNAs were extracted by Trizol reagent (Thermo Fisher Scientific, Waltham, MA, USA). The process included the removal of rRNA, synthesis of double‐stranded cDNA, end repair, degradation of one strand, and enrichment of the other strand by PCR. All sequencing were performed on the HiSeq 2000 sequencing platform (BGI‐Shenzhen, Shenzhen, China) according to the BGI commercial standard process (https://www.bgi.com/).^[^ ^28^ ^]^ Raw data were applied to the bioinformatic pipeline. To get high‐quality quality clean reads, reads were filtered by fastp (version 0.18.0) and mapped to the reference genome using HISAT2 (version 2.1.0). Transcriptome from RNA‐seq reads was reconstructed by Stringtie (version 1.3.4). In each transcription region, an FPKM (fragment per kilobase of transcript per million mapped reads) value was calculated to quantify its expression abundance and variations using RSEM (RNA‐Seq by Expectation Maximization) software. The libraries for quality control were built using the DNA 1000 assay Kit (Agilent Technologies, 5067‐1504) following the criteria: 1) concentration >2 ng µL^−1^, 2) a single peak shape with a size of 300–600 bp, and 3) the normally distributed library peak pattern. The differentially expressed mRNAs with differences >2‐fold and *p* <0.05 were generated from read counts using the online bioinformatic platform Dr. Tom provided by BGI (biosys.bgi.com/). Differentially expressed mRNAs were searched in the NCBI database (Gene Bank, Homo sapiens, GRCh38.p14) to determine their genome loci. These differentially expressed mRNAs were used for GO (Gene Onotology) analysis (http://geneontology.org/) to generate GO plots and for KEGG (Kyoto Encyclopedia of Genes and Genomes) analysis to generate KEGG pathway plots.

### Mice and Experimental Design

C57BL/6 mice with an age between 6 and 8 weeks (Charles River, Beijing, China) were raised in filter‐top cages with a 12 h light/dark cycle, autoclaved bedding, food, and water. C57BL/6 mice with a ratio of male to female of 1:3 were caged for mating overnight. The appearance of a vaginal plug was defined as day 1 (D1), which was further confirmed by monitoring their weight. To explore the effects of real environmental exposure dose (REED) of BPB on miscarriage, the actual daily intake dose of total bisphenols in the human body was calculated. The total levels of bisphenol analogues of 144 ng mL^−1^ in the serum of pregnant women in Beijing, China,^[^ ^22^ ^]^ could be considered as a representative of human real intake dose under the inadvertent environment. According to the body surface area coefficient (mouse/human = 12), the REED of BPB corresponded to 1.8 mg kg^−1^ day^−1^ BPB for the mouse. In animal models, subcutaneous injection of immature female rats with 200 mg kg^−1^ day^−1^ BPB altered the estrogenic effects.^[^ ^95^ ^]^ Treatment of male Sprague‐Dawley (35 days old) rats with 100 or 200 mg kg^−1^ day^−1^ BPB by oral gavage for 21 days decreased serum testosterone, luteinizing hormone, and follicle‐stimulating hormone levels, and also inhibited Leydig cell maturation in late puberty.^[^ ^92^ ^]^ Exposure of male rats to 50 mg kg^−1^ day^−1^ BPB for 28 days induced oxidative stress and toxic effects in testes and spermatogenesis.^[^ ^73^ ^]^ The LD50 of BPB in mice was estimated as 250 mg kg^−1^.^[^ ^62^ ^]^ Doses lower than LD50 were generally used to evaluate the acute toxicity responses in experimental animal models. Considering the doses used in literature, short lifespan of mice, and the feasibility of mouse model assays, in this study, it was selected that 0, 1.8 mg kg^−1^ day^−1^ (1‐fold REED), 18 mg kg^−1^ day^−1^ (10‐fold REED), 50 mg kg^−1^ day^−1^ (28‐fold REED, 1/5 of LD_50_, medium‐dose group), or 100 mg kg^−1^ day^−1^ (56‐fold REED, 2/5 of LD_50_, high‐dose group) BPB to construct BPB‐exposed mouse model (*n* = 6). Corn oil was used as a solvent of BPB. Two mouse miscarriage intervention models were also constructed.

**Model 1**, BPB‐exposed pregnant mice model was constructed: ① control group, treated with the same volume of saline solution; ② 1‐fold REED of BPB group, treated with 1.8 mg kg^−1^ d^−1^ BPB; ③ 10‐fold REED of BPB group, treated with 18 mg kg^−1^ d^−1^ LiCl; ④ 28‐fold REED of BPB group, treated with 50 mg kg^−1^ d^−1^ BPB; ⑤ 56‐fold REED of BPB group, treated with 100 mg kg^−1^ d^−1^ BPB. Mice were daily given BPB or an equal volume of corn oil by oral gavage from D1 to D13.

**Model 2**, Pkca intervention mouse model. Pregnant mice were randomly divided into four groups (each *n* = 6): 1) corn oil group, 2) BPB group, 3) BPB + Vector group, 4) BPB + pcDNA3.1‐Pkca group.

**Model 3**, Tspan4 intervention mouse model. Pregnant mice were randomly divided into two groups (each *n* = 6): 1) BPB + Vector group, 2) BPB + pcDNA3.1‐Tspan4 group.

Six groups were daily given 100 mg kg^−1^ BPB or corn oil from D1 to D13 by oral gavage. In addition, mice were also intraperitoneally injected with 20 mg kg^−1^ pcDNA3.1‐Pkca, pcDNA3.1‐Tspan4, or Vector (negative control of pcDNA3.1) once per three days from D1 to D13. Sequences of pcDNA3.1‐Pkca and pcDNA3.1‐Tspan4 were shown in Table S10 (Supporting Information). All the mice were euthanized by injection with nembutal (100 mg kg^−1^) for the collection of the uterus on D14. The miscarriage rate in each mouse was calculated as the number of embryo resorption divided by the number of total embryos, which had been widely used in the own papers and in other literature.^[^ ^50^, ^96^, ^97^ ^]^ The average miscarriage rate in each group was calculated. Alternatively, the ratios of the adsorbed embryo number vs the normal embryo number were also calculated to reflect miscarriage. A random placenta was collected from every mouse in each group for RNA and protein extraction. The research protocols were approved by the Medical Ethics Committee of the Eighth Affiliated Hospital, Sun Yat‐sen University.

### Enzyme‐Linked Immunosorbent Assay (ELISA) Analysis

The protein levels of TNF‐α, IL‐6, and IL‐1β in serum samples of pregnant mouse and mouse transfected with empty vector plasmid were analyzed using TNF‐α ELISA kits (RK00027, ABclonal Technology), IL‐6 ELISA kits (RK00008, ABclonal Technology), and IL‐1β ELISA kits (RK00006, ABclonal Technology), respectively. The purified mouse TNF‐α, IL‐6, or IL‐1β antibody was coated onto a microplate to form a solid‐phase antibody. Standard substance and serum samples were mixed in microplates at 37 °C for 2 h. After washing microplates thrice, the microplates were incubated with biotinylated TNF‐α, IL‐6, or IL‐1β antibody working solution for 1 h at 37 °C. HRP‐labeled TNF‐α, IL‐6, or IL‐1β antibody was then added to the microplate, and the mixture was incubated at 37 °C for 0.5 h. After thorough washing, TMB (3, 3′, 5, 5′‐Tetramethylbenzidine) substrate was added to the microplate. After incubation for 20 min, the absorbance was measured at 450 nm with a microplate reader. The concentrations of TNF‐α, IL‐6, and IL‐1β in serum (pg mL^−1^) were calculated based on a corresponding standard curve.

### Molecular Docking

Modeller v10.4^[^ ^98^, ^99^ ^]^ was used for homology modeling to repair the missing side chains of protein ER (PDB ID: 5gs4). Using the SAVES server (https://saves.mbi.ucla.edu/), Ramachandran plots were generated to assess the rationality of protein conformation. All amino acid residues were found within the allowed regions. Double‐stranded DNA (dsDNA) was built by Discovery Studio 2019 with the sequences 5′‐GGCATGAGCCACCCTGCCCA‐3′ and 3′‐CCGTACTCGGTGGGACGGGT‐5′. The dsDNA was docked to the protein ER using HDOCK v1.1.^[^ ^100^ ^]^ The docking results were assessed by the docking score and confidence score, which were −239.72 and 0.8575 in this study, respectively. A more negative docking score indicated a more likely binding. Confidence score >0.7 meant very high probably binding, 0.5–0.7 meant moderate binding, and <0.5 meant less likely binding. The BPB 3D structure was made using ChemDraw (20.0). Molecular docking of BPB with ER using AutoDock Vina 1.2.5. Protein 3D structures were made by PyMOL v2.5.4.

### Molecular Dynamics

The binding stability of the complex was further analyzed by MD simulation using GROMACS v2020.6. The Amber03 force field was utilized to generate parameters and topology files for proteins and nucleic acids. The Amber GAFF force field was used for BPB. A periodic boundary condition (PBC) was set, and the simulation box dimensions were optimized. The protein was placed at the center of a cubic box with a minimum distance of 1.0 nm to the box edges, and the box was filled with water molecules (with TIP 3‐point as template). To maintain system neutrality, solvent water molecules were partially replaced with Na^+^ and Cl^−^ ions at a concentration of 0.15 m. The entire system's energy was minimized using the steepest descent minimization method to ensure a reasonable initial geometric configuration and solvent molecule orientation. To initiate actual dynamic simulations, pre‐equilibration of solvent and ions around the protein was performed to achieve a stable thermodynamic ensemble under desired conditions. The pre‐equilibration comprised two stages: to stabilize the system's temperature using an NVT system at 300 K for 100 ps and to stabilize the system's pressure using an NPT system at 1 bar for 100 ps. A leapfrog algorithm was employed for dynamic integration equilibration, and all MD simulations were conducted under isothermal‐isobaric conditions at 300 K and 1 bar and lasted for 100 ns. Subsequently, trajectory correction was performed with the protein as the center to conduct Root Mean Square Deviation (RMSD) analysis, which reflected the structural differences between the simulated and the initial structure as the reference. A smaller RMSD indicated greater similarity between the simulated and reference structures. Before RMSD calculation, structures were superimposed onto the reference structure using the least squares method to eliminate overall system motion. The Gibbs free‐energy change of binding between target‐lead was computed using the Molecular Mechanics Poisson‐Boltzmann Surface Area (MMPBSA) method implemented in the g_mmpbsa tool.

### Statistical Analysis

All experiments were independently performed thrice and the results were expressed as mean ± SD (standard deviation, *n* = 3). Statistical analysis was performed using one‐way analysis of variance with SPSS v24.0 software. The significance of differences between the two groups were determined using the LSD *t*‐test. Dunnett's or LSD test was used to examine the statistical difference among three or more groups. The Pearson test was used for correlation analysis. The odds ratio (OR) of each variable and the risk of unexplained miscarriage and its 95% confidence interval (95% CI) before adjusting potential confounders were analyzed by univariate logistic regression analysis. The OR and 95% CI after adjusting potential confounders were analyzed by multivariate logistic regression analysis. ROC curves were analyzed by SPSS v24.0 software and plotted using Graphpad Prism 8.0.2. ROC AUC was calculated as the area under the ROC curve. To examine the robustness of the association between BPB levels and miscarriage, the interactions between BPB levels and subpopulation indicators (e.g., age, BMI, education, household income, smoking, drinking, and taking canned food and bottled drinks) were explored.^[^ ^101^ ^]^ Restricted cubic spline (RCS) models were constructed based on the plot RCS and the knots were calculated using the 5, 50, or 95th of their levels, with the median values as the reference. The statistics and graphs were conducted using Graphpad Prism 8.0.2 *p* ≤ 0.05 was considered as significant difference.

## Conflict of Interest

The authors declare no conflict of interest.

## Author Contributions

W.H., M.W., and Y.S. contributed equally to this work. W.H., M.W., Y.S., X.S., and H.Z. designed the study. W.H., M.W., and Y.S. performed most of the experiments. W.H., M.W., Y.S., X.S., and H.Z. analyzed data and wrote the draft manuscript. X.Y. made molecular docking. Y.L., Q.F., and S.X. conducted the partial experiments. X.L., G.G., D.Z., X.M., and Z.Z. contributed to data analysis and constructive comments.

## Supporting information



Supporting Information

Supporting Information

Supporting Information

## Data Availability

The National Center for Biotechnology Information (NCBI) Blast (https://blast.ncbi.nlm.nih.gov/Blast.cgi), UCSC Genome Browser (http://genome.ucsc.edu), PROMO software Version 3.0.2 (http://alggen.lsi.upc.es/cgi‐bin/promo_v3/promo/promoinit.cgi?dirDB=TF_8.3), KEGG compound database (https://www.genome.jp/kegg/compound/), GO (Gene Onotology) analysis (http://geneontology.org/), RCSB PDB (https://www.rcsb.org/), and SAVES server (https://saves.mbi.ucla.edu/) were used in this study. The data supporting the findings of this study are available within the article and its Supplementary Information files and source data file.
