# Supplementary material for: Bisphenol B Exposure Induces Miscarriage by Suppressing Migration/Invasion and Migrasome Formation
Source: Adv Sci (Weinh). 2025 Nov 21;13(7):e04871. doi: 10.1002/advs.202504871 (PMC12866717; doi:10.1002/advs.202504871)
Supplement: Supplementary file 1 — Supporting Information [file ADVS-13-e04871-s001.pdf]

Fig. S1

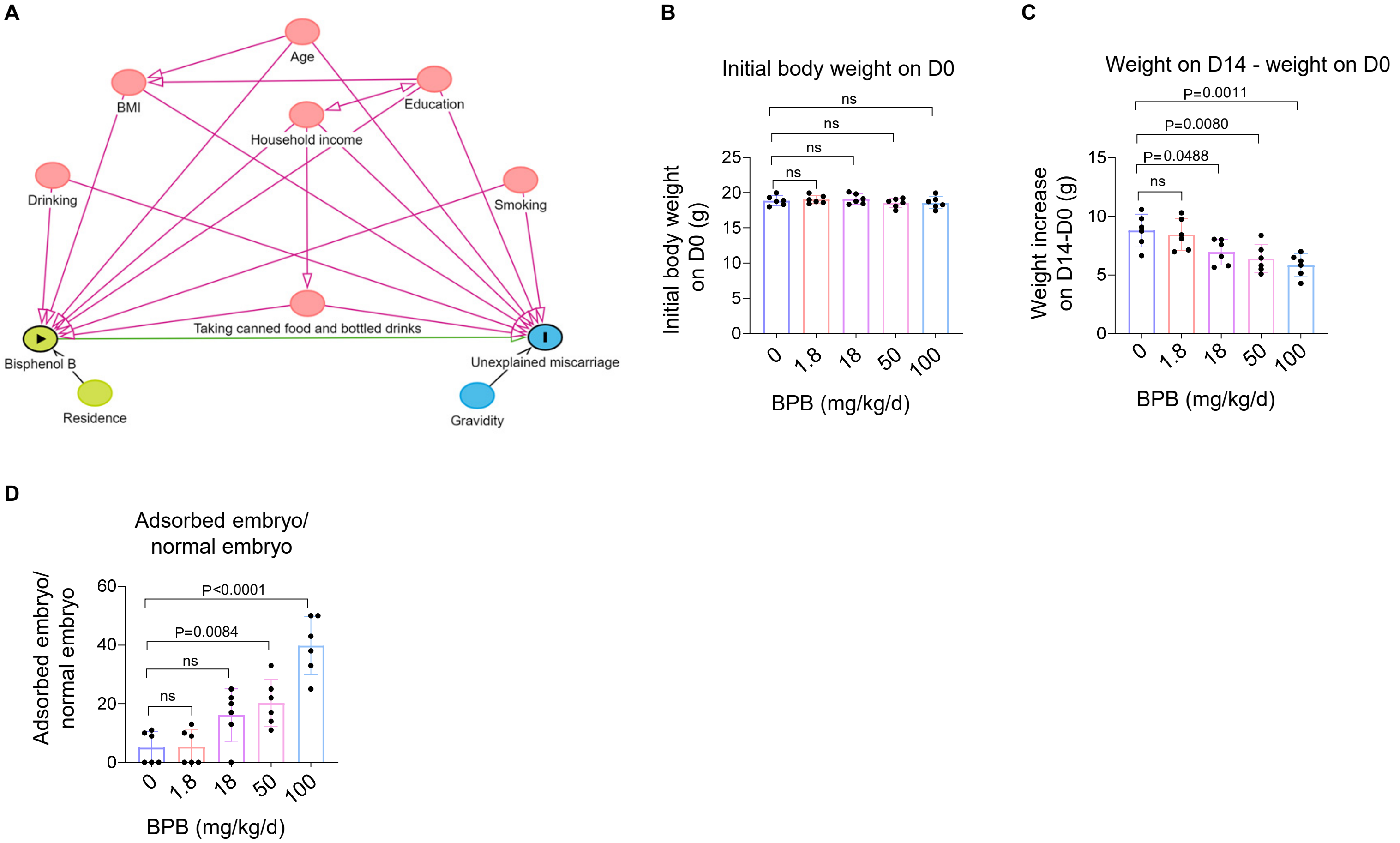
A
Age
BMI
Education
Household income
Drinking
Smoking
Taking canned food and bottled drinks
Bisphenol B
Unexplained miscarriage
Residence
Gravidity
B
Initial body weight on D0
Initial body weight on D0 (g)
BPB (mg/kg/d)
ns
ns
ns
ns
C
Weight on D14 - weight on D0
Weight increase on D14-D0 (g)
BPB (mg/kg/d)
P=0.0011
P=0.0080
P=0.0488
ns
D
Adsorbed embryo/ normal embryo
Adsorbed embryo/ normal embryo
BPB (mg/kg/d)
P<0.0001
P=0.0084
ns
ns

Fig. S2

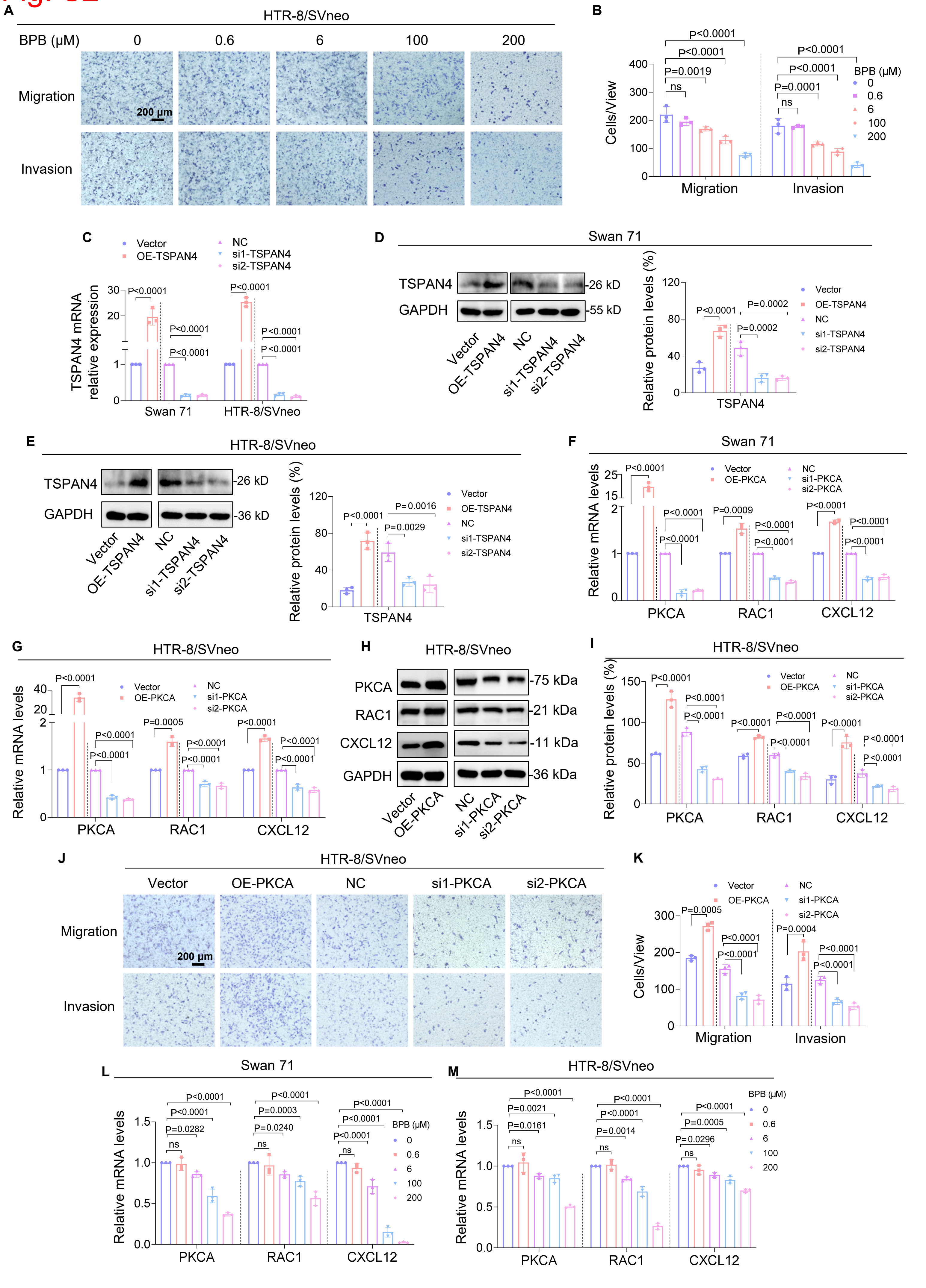

A
HTR-8/SVneo
BPB (μM) 0 0.6 6 100 200
Migration
Invasion
200 μm
B
Cells/View
Migration
Invasion
BPB (μM)
C
TSPAN4 mRNA relative expression
Swan 71
HTR-8/SVneo
Vector
OE-TSPAN4
NC
si1-TSPAN4
si2-TSPAN4
D
Swan 71
TSPAN4
GAPDH
-26 kD
-55 kD
Relative protein levels (%)
E
HTR-8/SVneo
-36 kD
F
Swan 71
Relative mRNA levels
PKCA
RAC1
CXCL12
OE-PKCA
si1-PKCA
si2-PKCA
G
HTR-8/SVneo
H
HTR-8/SVneo
-75 kDa
-21 kDa
-11 kDa
-36 kDa
I
HTR-8/SVneo
J
HTR-8/SVneo
K
L
Swan 71
M
HTR-8/SVneo

Fig. S2

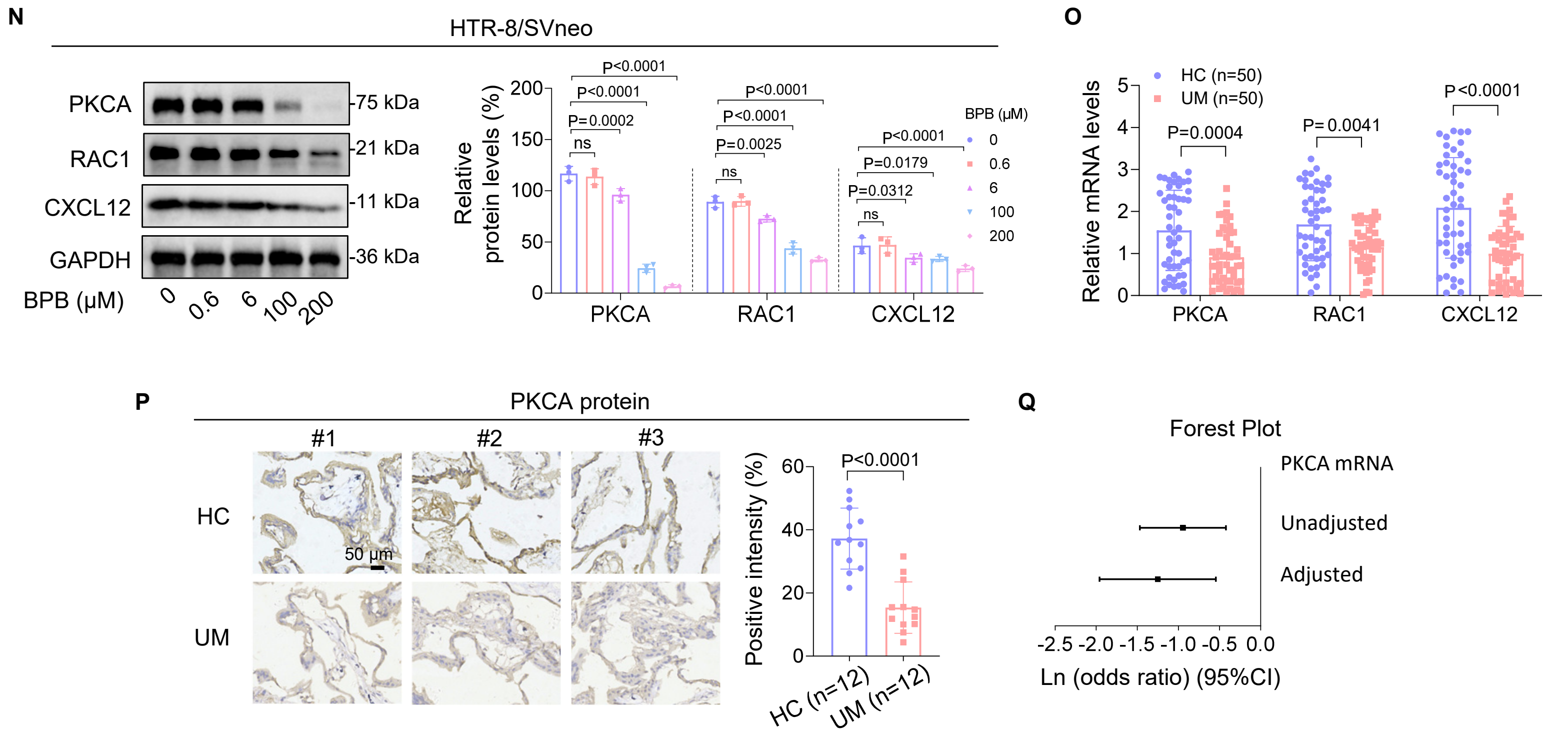
N
HTR-8/SVneo
PKCA
RAC1
CXCL12
GAPDH
75 kDa
21 kDa
11 kDa
36 kDa
BPB (μM)
0
0.6
6
100
200
Relative protein levels (%)
BPB (μM)
P<0.0001
P=0.0002
P=0.0025
P=0.0179
P=0.0312
ns
PKCA
RAC1
CXCL12
O
HC (n=50)
UM (n=50)
Relative mRNA levels
P=0.0004
P=0.0041
P<0.0001
P
PKCA protein
#1
#2
#3
HC
UM
50 μm
Positive intensity (%)
HC (n=12)
UM (n=12)
Q
Forest Plot
PKCA mRNA
Unadjusted
Adjusted
Ln (odds ratio) (95%CI)

Fig. S3-1

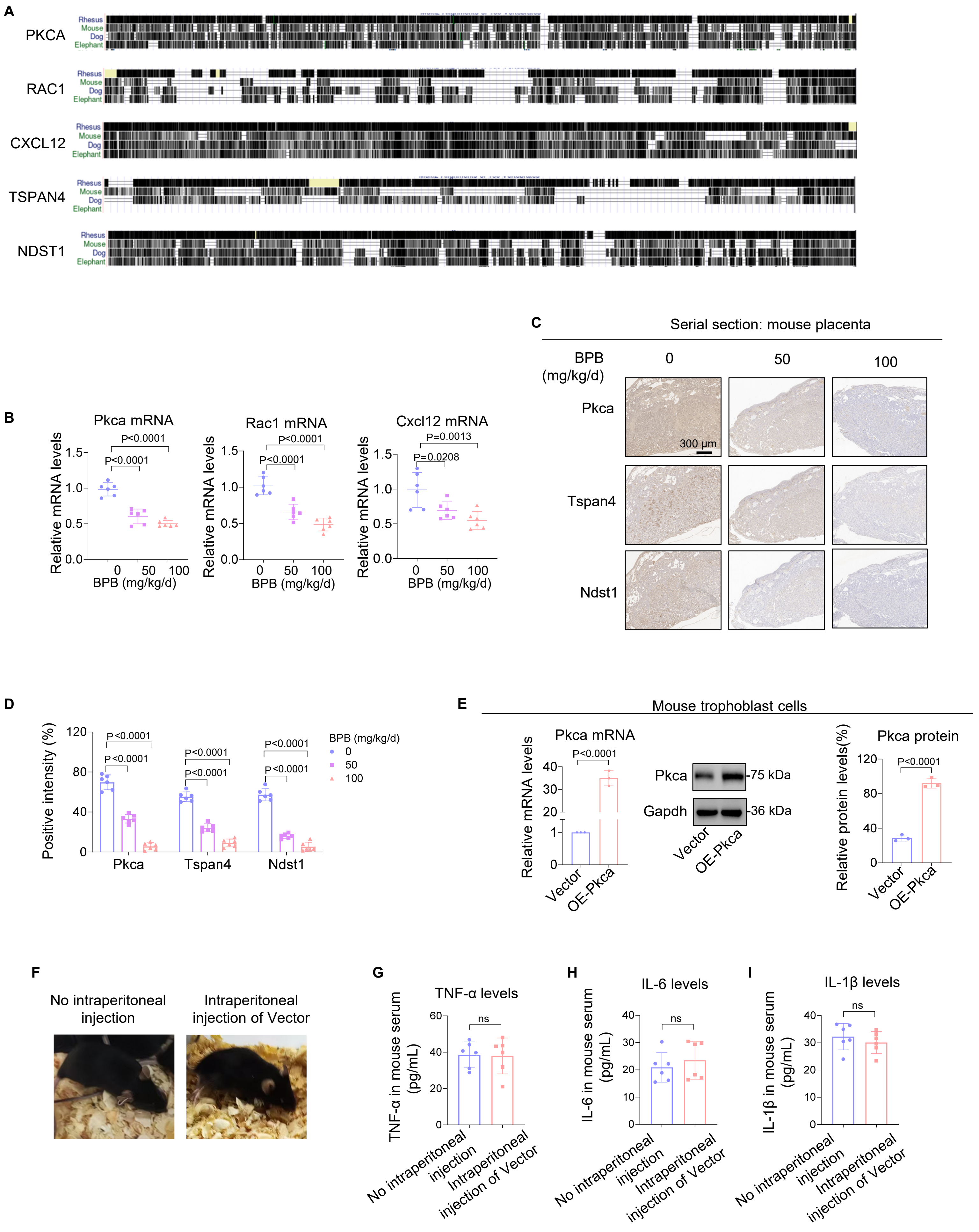

Fig. S3-2

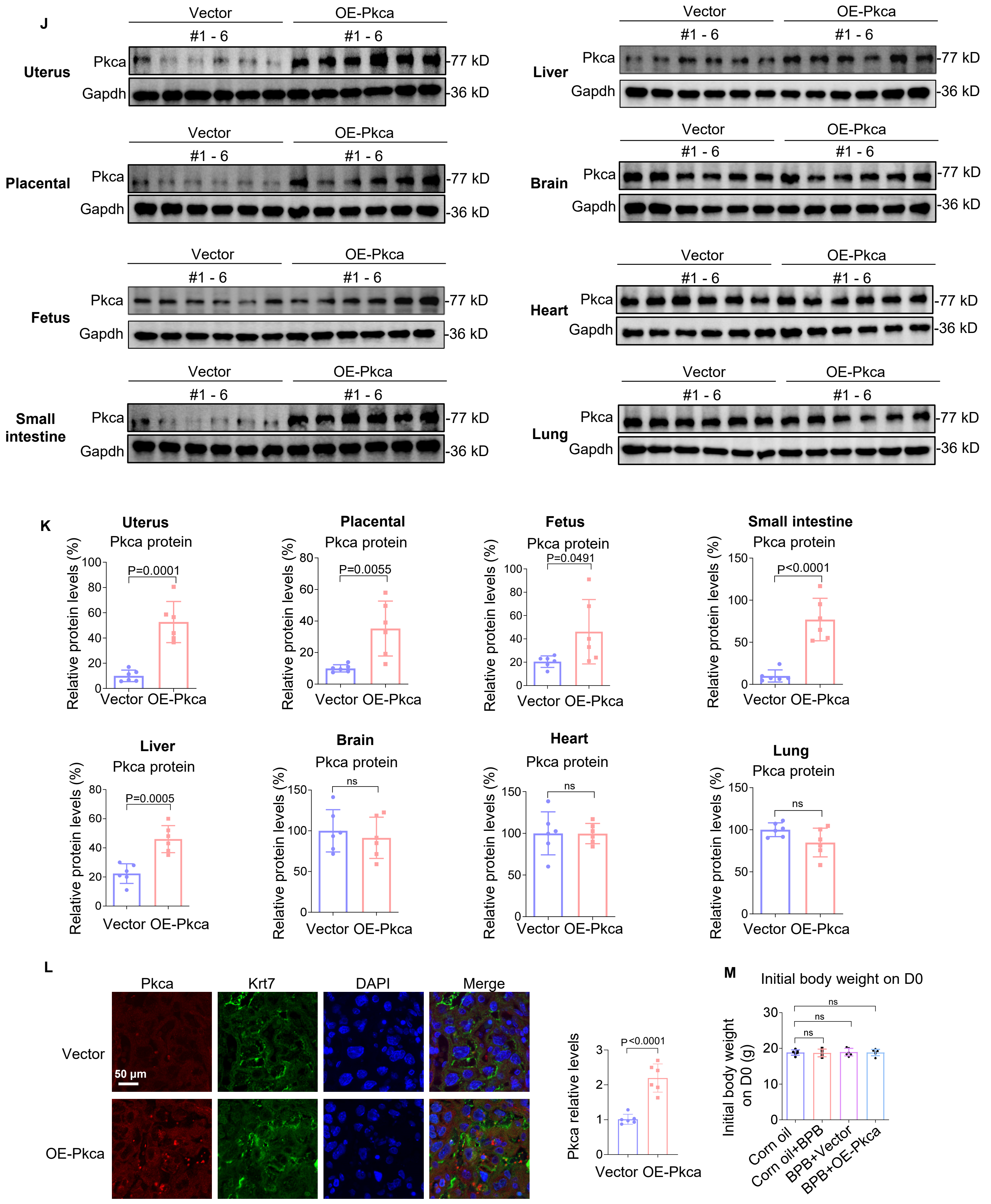

J
Uterus
Placental
Fetus
Small intestine
Liver
Brain
Heart
Lung
Vector
OE-Pkca
#1 - 6
Pkca
Gapdh
-77 kD
-36 kD
K
Pkca protein
Relative protein levels (%)
P=0.0001
P=0.0055
P=0.0491
P<0.0001
P=0.0005
ns
Vector OE-Pkca
L
Pkca
Krt7
DAPI
Merge
50 μm
Pkca relative levels
P<0.0001
M
Initial body weight on D0
Initial body weight on D0 (g)
Corn oil
Corn oil+BPB
BPB+Vector
BPB+OE-Pkca

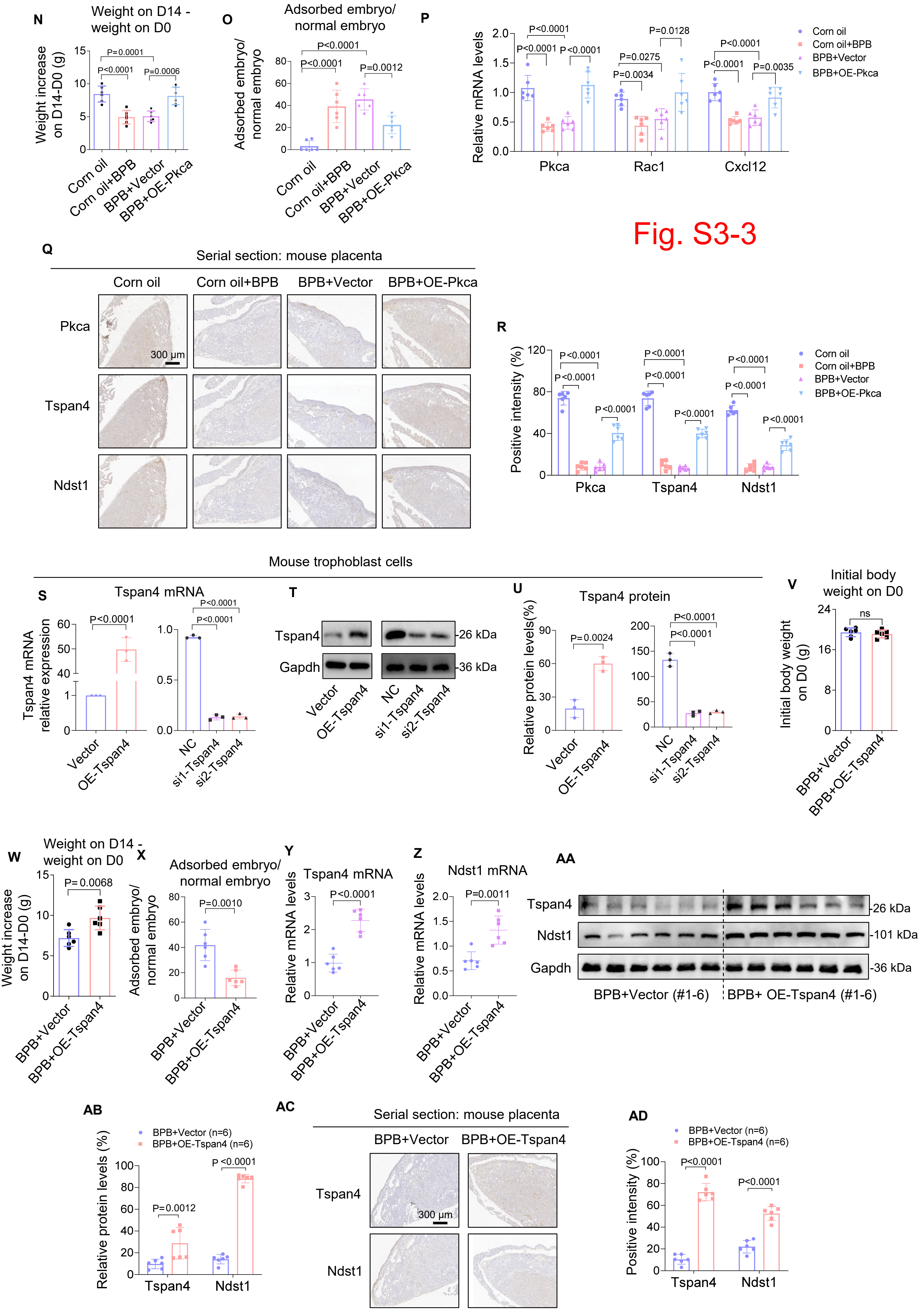
N
Weight on D14 - weight on D0
Weight increase on D14-D0 (g)
P=0.0001
P<0.0001
P=0.0006
Corn oil
Corn oil+BPB
BPB+Vector
BPB+OE-Pkca
O
Adsorbed embryo/ normal embryo
Adsorbed embryo/ normal embryo
P<0.0001
P<0.0001
P=0.0012
P
Relative mRNA levels
P<0.0001
P<0.0001
P<0.0001
P=0.0128
P=0.0275
P=0.0034
P<0.0001
P<0.0001
P=0.0035
Pkca
Rac1
Cxcl12
Q
Serial section: mouse placenta
Corn oil
Corn oil+BPB
BPB+Vector
BPB+OE-Pkca
Pkca
Tspan4
Ndst1
300 μm
R
Positive intensity (%)
P<0.0001
Mouse trophoblast cells
S
Tspan4 mRNA
Tspan4 mRNA relative expression
P<0.0001
Vector
OE-Tspan4
NC
si1-Tspan4
si2-Tspan4
T
Tspan4
Gapdh
26 kDa
36 kDa
U
Tspan4 protein
Relative protein levels(%)
P=0.0024
P<0.0001
V
Initial body weight on D0
Initial body weight on D0 (g)
ns
BPB+Vector
BPB+OE-Tspan4
W
Weight on D14 - weight on D0
Weight increase on D14-D0 (g)
P= 0.0068
X
Adsorbed embryo/ normal embryo
Adsorbed embryo/ normal embryo
P=0.0010
Y
Tspan4 mRNA
Relative mRNA levels
P<0.0001
Z
Ndst1 mRNA
Relative mRNA levels
P=0.0011
AA
Tspan4
Ndst1
Gapdh
26 kDa
101 kDa
36 kDa
BPB+Vector (#1-6)
BPB+ OE-Tspan4 (#1-6)
AB
BPB+Vector (n=6)
BPB+OE-Tspan4 (n=6)
Relative protein levels (%)
P=0.0012
P <0.0001
Tspan4
Ndst1
AC
Serial section: mouse placenta
BPB+Vector
BPB+OE-Tspan4
Tspan4
Ndst1
300 μm
AD
BPB+Vector (n=6)
BPB+OE-Tspan4 (n=6)
Positive intensity (%)
P<0.0001
P<0.0001
Tspan4
Ndst1

Fig. S3-3

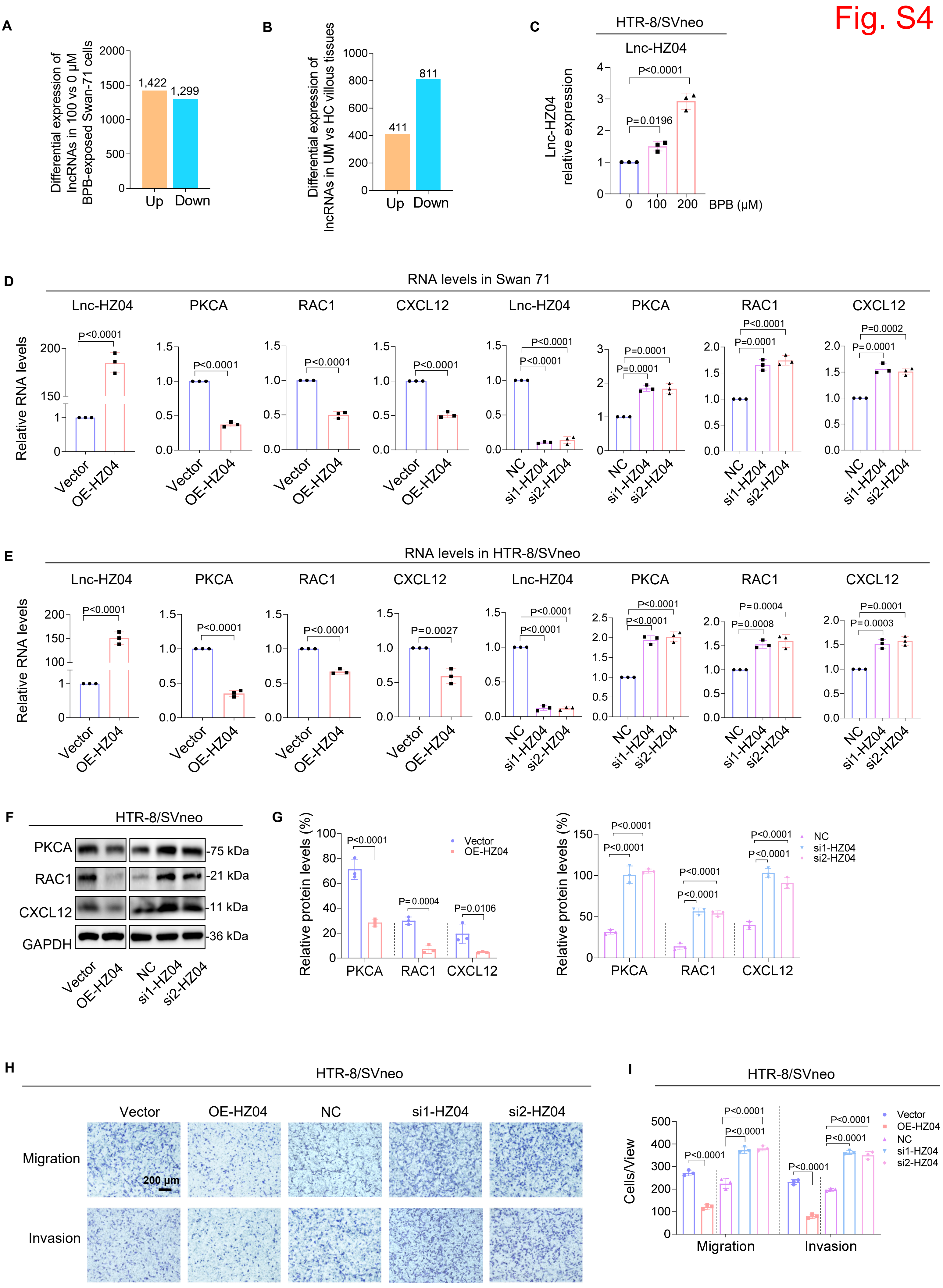
Fig. S4
A
Differential expression of lncRNAs in 100 vs 0 μM BPB-exposed Swan-71 cells
1,422
1,299
Up
Down
B
Differential expression of lncRNAs in UM vs HC villous tissues
411
811
Up
Down
C
HTR-8/SVneo
Lnc-HZ04
Lnc-HZ04 relative expression
P<0.0001
P=0.0196
BPB (μM)
D
RNA levels in Swan 71
Lnc-HZ04
PKCA
RAC1
CXCL12
Relative RNA levels
Vector
OE-HZ04
NC
si1-HZ04
si2-HZ04
E
RNA levels in HTR-8/SVneo
F
HTR-8/SVneo
PKCA
RAC1
CXCL12
GAPDH
-75 kDa
-21 kDa
-11 kDa
-36 kDa
G
Relative protein levels (%)
H
HTR-8/SVneo
Migration
Invasion
200 μm
I
HTR-8/SVneo
Cells/View

Fig. S5

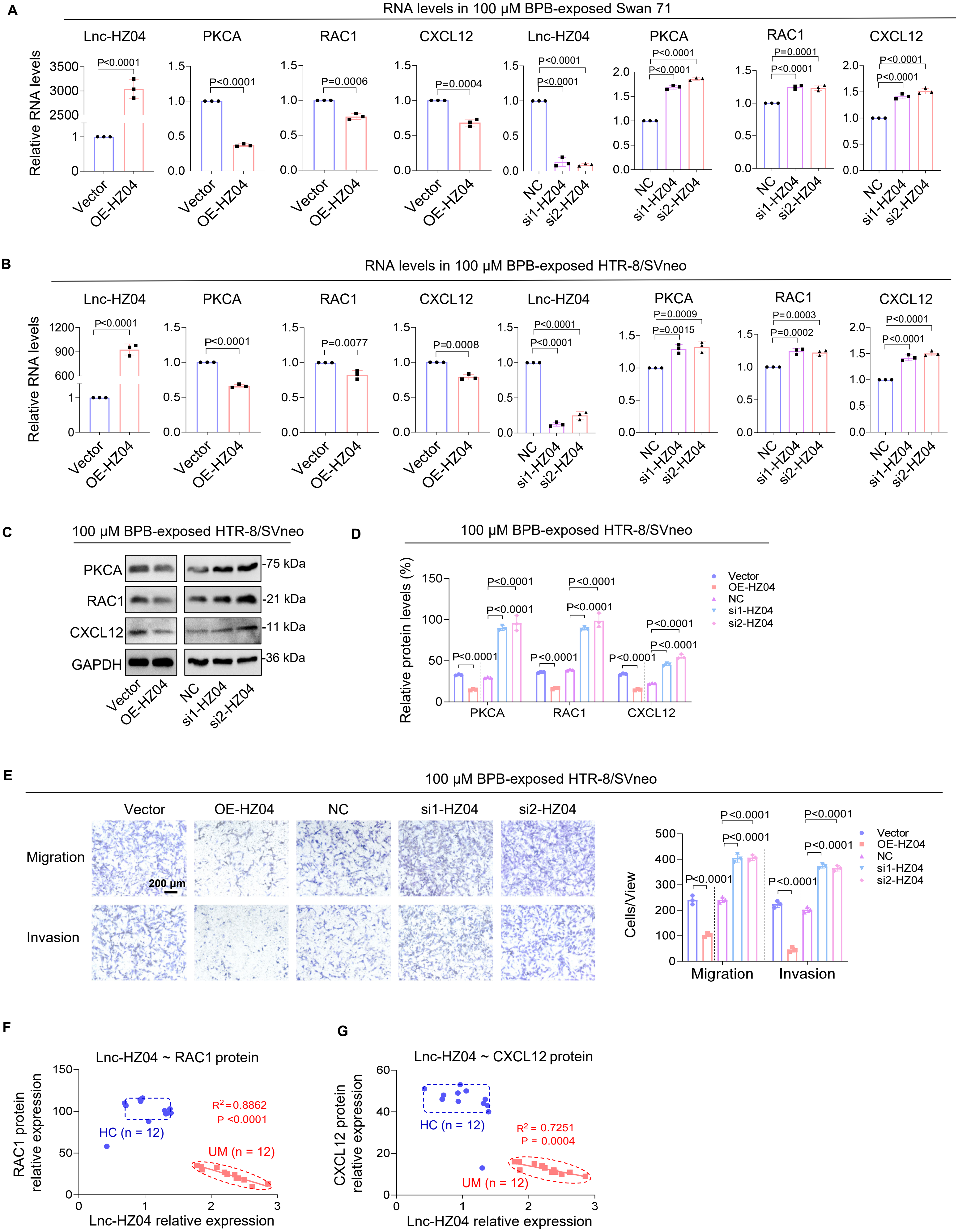

A
RNA levels in 100 μM BPB-exposed Swan 71
Lnc-HZ04
PKCA
RAC1
CXCL12
Relative RNA levels
Vector
OE-HZ04
NC
si1-HZ04
si2-HZ04
B
RNA levels in 100 μM BPB-exposed HTR-8/SVneo
C
100 μM BPB-exposed HTR-8/SVneo
GAPDH
-75 kDa
-21 kDa
-11 kDa
-36 kDa
D
100 μM BPB-exposed HTR-8/SVneo
Relative protein levels (%)
E
100 μM BPB-exposed HTR-8/SVneo
Migration
Invasion
200 μm
Cells/View
F
Lnc-HZ04 ~ RAC1 protein
RAC1 protein relative expression
HC (n = 12)
UM (n = 12)
R2=0.8862
P <0.0001
Lnc-HZ04 relative expression
G
Lnc-HZ04 ~ CXCL12 protein
CXCL12 protein relative expression
R2 = 0.7251
P = 0.0004

Fig. S6-1

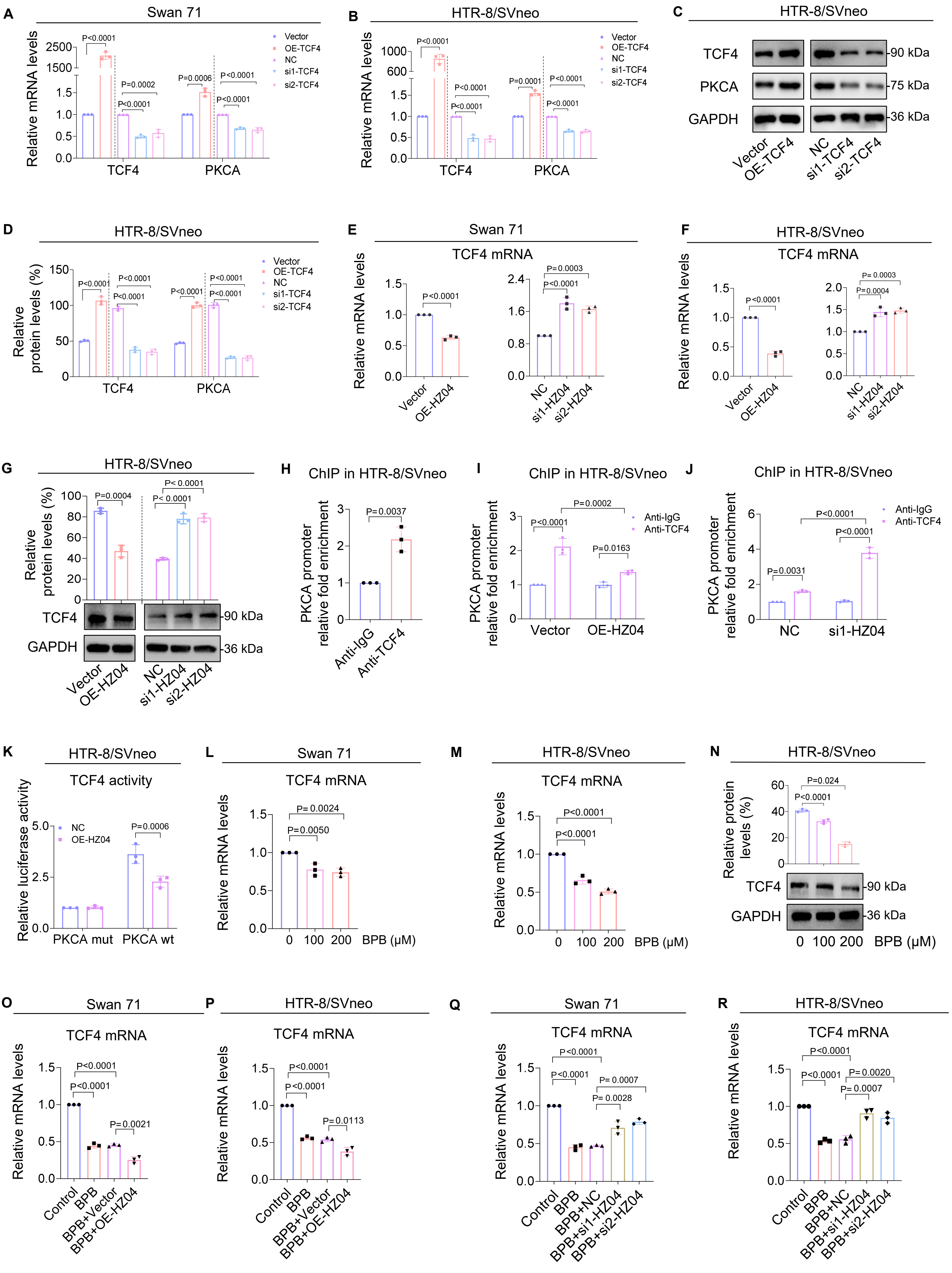

A
Swan 71
Relative mRNA levels
Vector
OE-TCF4
NC
si1-TCF4
si2-TCF4
P<0.0001
P=0.0002
P<0.0001
P=0.0006
P<0.0001
P<0.0001
TCF4
PKCA
B
HTR-8/SVneo
Relative mRNA levels
P<0.0001
P<0.0001
P<0.0001
P=0.0001
P<0.0001
P<0.0001
TCF4
PKCA
C
HTR-8/SVneo
TCF4
PKCA
GAPDH
-90 kDa
-75 kDa
-36 kDa
Vector
OE-TCF4
NC
si1-TCF4
si2-TCF4
D
HTR-8/SVneo
Relative protein levels (%)
P<0.0001
P<0.0001
P<0.0001
P<0.0001
P<0.0001
P<0.0001
TCF4
PKCA
E
Swan 71
TCF4 mRNA
Relative mRNA levels
P<0.0001
P=0.0003
P<0.0001
Vector
OE-HZ04
NC
si1-HZ04
si2-HZ04
F
HTR-8/SVneo
TCF4 mRNA
Relative mRNA levels
P<0.0001
P= 0.0003
P=0.0004
Vector
OE-HZ04
NC
si1-HZ04
si2-HZ04
G
HTR-8/SVneo
Relative protein levels (%)
P=0.0004
P< 0.0001
P< 0.0001
TCF4
GAPDH
-90 kDa
-36 kDa
Vector
OE-HZ04
NC
si1-HZ04
si2-HZ04
H
ChIP in HTR-8/SVneo
PKCA promoter relative fold enrichment
P=0.0037
Anti-IgG
Anti-TCF4
I
ChIP in HTR-8/SVneo
PKCA promoter relative fold enrichment
P=0.0002
P<0.0001
P=0.0163
Anti-IgG
Anti-TCF4
Vector
OE-HZ04
J
ChIP in HTR-8/SVneo
PKCA promoter relative fold enrichment
Anti-IgG
Anti-TCF4
P<0.0001
P<0.0001
P=0.0031
NC
si1-HZ04
K
HTR-8/SVneo
TCF4 activity
Relative luciferase activity
NC
OE-HZ04
P=0.0006
PKCA mut
PKCA wt
L
Swan 71
TCF4 mRNA
Relative mRNA levels
P= 0.0024
P=0.0050
0
100
200
BPB (μM)
M
HTR-8/SVneo
TCF4 mRNA
Relative mRNA levels
P<0.0001
P<0.0001
0
100
200
BPB (μM)
N
HTR-8/SVneo
Relative protein levels (%)
P=0.024
P<0.0001
TCF4
GAPDH
-90 kDa
-36 kDa
0
100
200
BPB (μM)
O
Swan 71
TCF4 mRNA
Relative mRNA levels
P<0.0001
P<0.0001
P=0.0021
Control
BPB
BPB+Vector
BPB+OE-HZ04
P
HTR-8/SVneo
TCF4 mRNA
Relative mRNA levels
P<0.0001
P<0.0001
P=0.0113
Control
BPB
BPB+Vector
BPB+OE-HZ04
Q
Swan 71
TCF4 mRNA
Relative mRNA levels
P<0.0001
P<0.0001
P= 0.0007
P= 0.0028
Control
BPB
BPB+NC
BPB+si1-HZ04
BPB+si2-HZ04
R
HTR-8/SVneo
TCF4 mRNA
Relative mRNA levels
P<0.0001
P<0.0001
P= 0.0020
P= 0.0007
Control
BPB
BPB+NC
BPB+si1-HZ04
BPB+si2-HZ04

Fig. S6-2

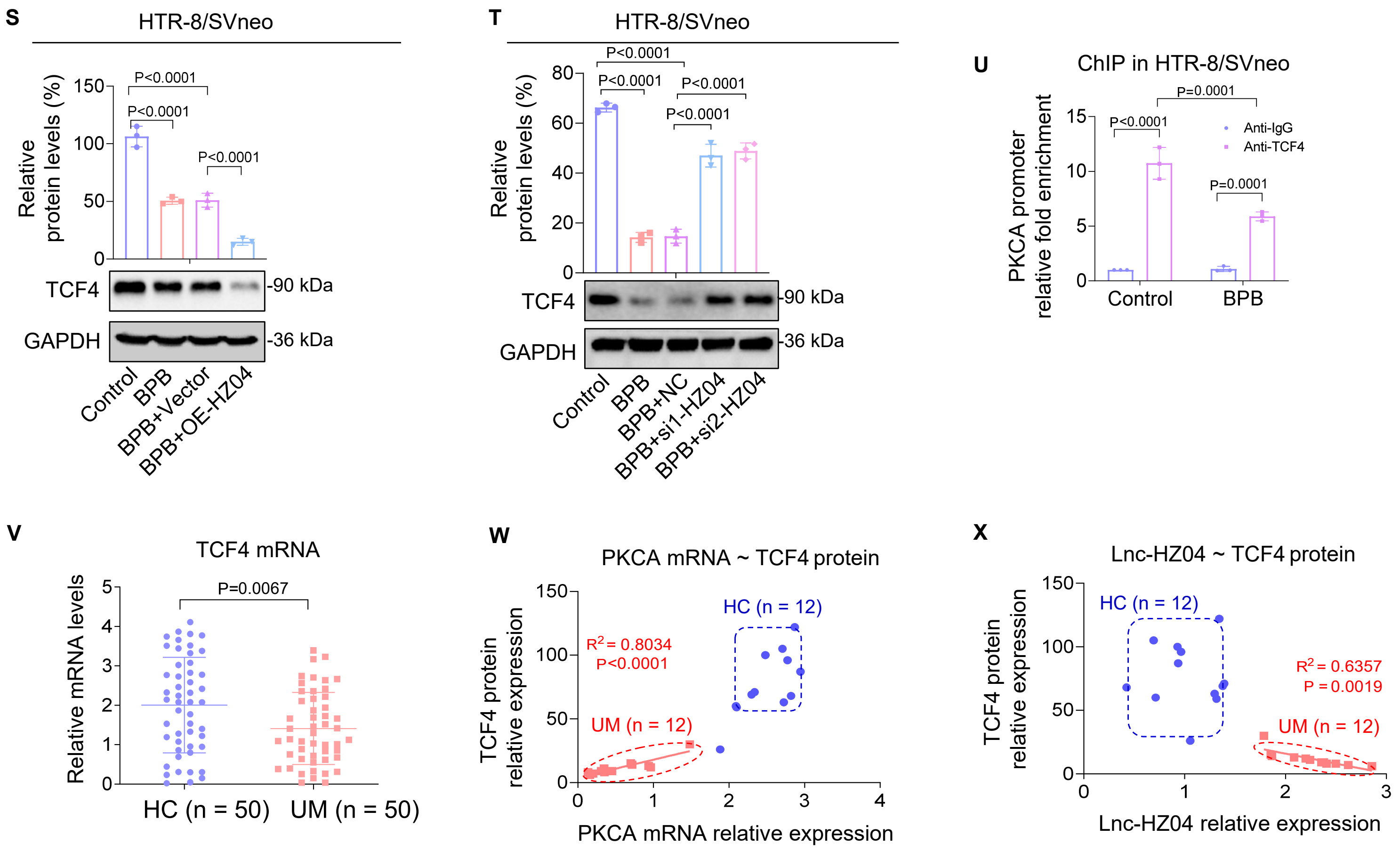
S
HTR-8/SVneo
Relative protein levels (%)
P<0.0001
P<0.0001
P<0.0001
TCF4
-90 kDa
GAPDH
-36 kDa
Control
BPB
BPB+Vector
BPB+OE-HZ04
T
HTR-8/SVneo
Relative protein levels (%)
P<0.0001
P<0.0001
P<0.0001
P<0.0001
TCF4
-90 kDa
GAPDH
-36 kDa
Control
BPB
BPB+NC
BPB+si1-HZ04
BPB+si2-HZ04
U
ChIP in HTR-8/SVneo
PKCA promoter relative fold enrichment
P<0.0001
P=0.0001
P=0.0001
Anti-IgG
Anti-TCF4
Control
BPB
V
TCF4 mRNA
Relative mRNA levels
P=0.0067
HC (n = 50)
UM (n = 50)
W
PKCA mRNA ~ TCF4 protein
TCF4 protein relative expression
HC (n = 12)
R2 = 0.8034
P<0.0001
UM (n = 12)
PKCA mRNA relative expression
X
Lnc-HZ04 ~ TCF4 protein
TCF4 protein relative expression
HC (n = 12)
R2 = 0.6357
P = 0.0019
UM (n = 12)
Lnc-HZ04 relative expression

Fig. S7

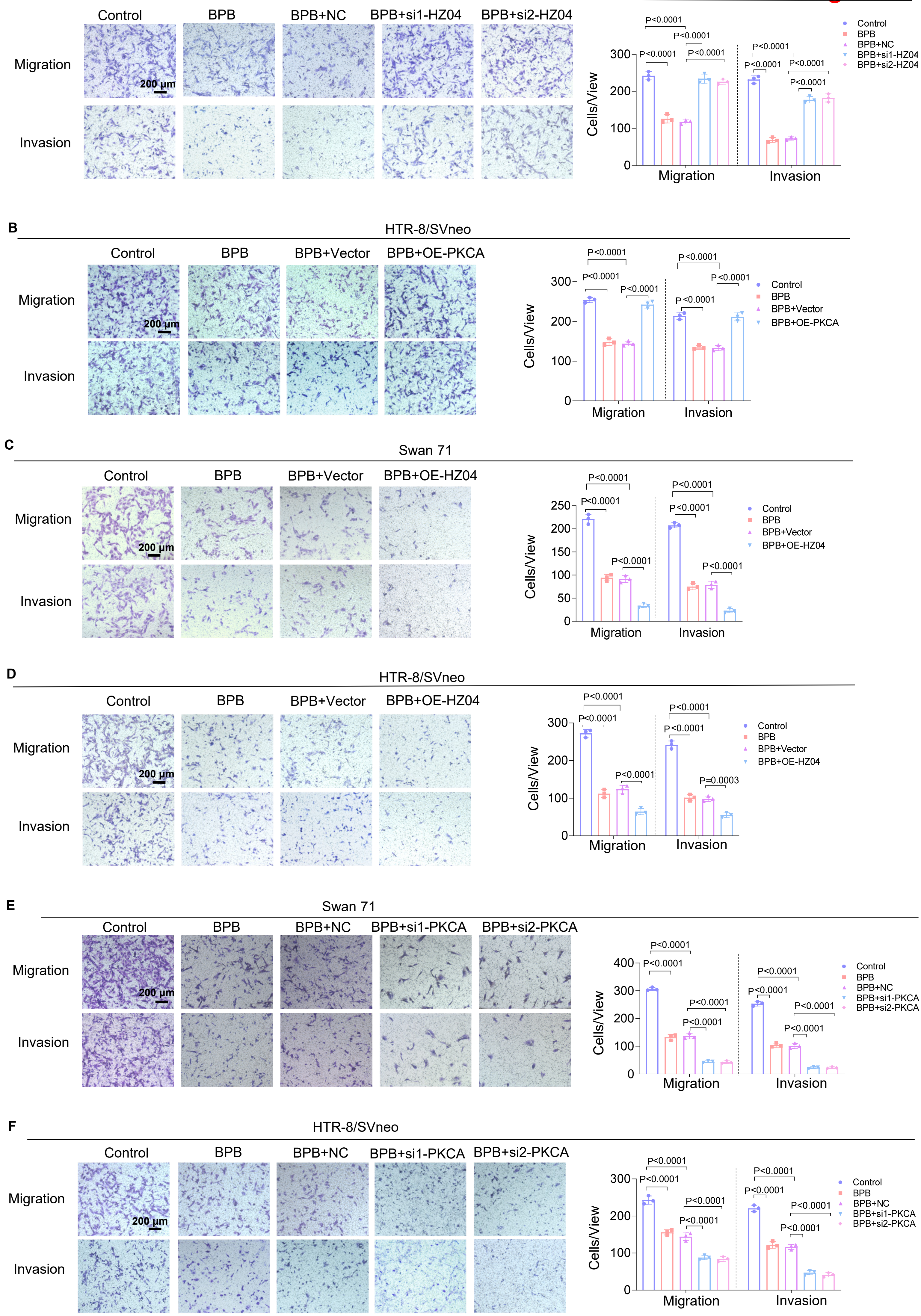
A
HTR-8/SVneo
Control
BPB
BPB+NC
BPB+si1-HZ04
BPB+si2-HZ04
Migration
Invasion
200 μm
Cells/View
P<0.0001
B
HTR-8/SVneo
Control
BPB
BPB+Vector
BPB+OE-PKCA
Migration
Invasion
200 μm
Cells/View
P<0.0001
C
Swan 71
Control
BPB
BPB+Vector
BPB+OE-HZ04
Migration
Invasion
200 μm
Cells/View
P<0.0001
D
HTR-8/SVneo
Control
BPB
BPB+Vector
BPB+OE-HZ04
Migration
Invasion
200 μm
Cells/View
P<0.0001
P=0.0003
E
Swan 71
Control
BPB
BPB+NC
BPB+si1-PKCA
BPB+si2-PKCA
Migration
Invasion
200 μm
Cells/View
P<0.0001
F
HTR-8/SVneo
Control
BPB
BPB+NC
BPB+si1-PKCA
BPB+si2-PKCA
Migration
Invasion
200 μm
Cells/View
P<0.0001

Fig. S8-1

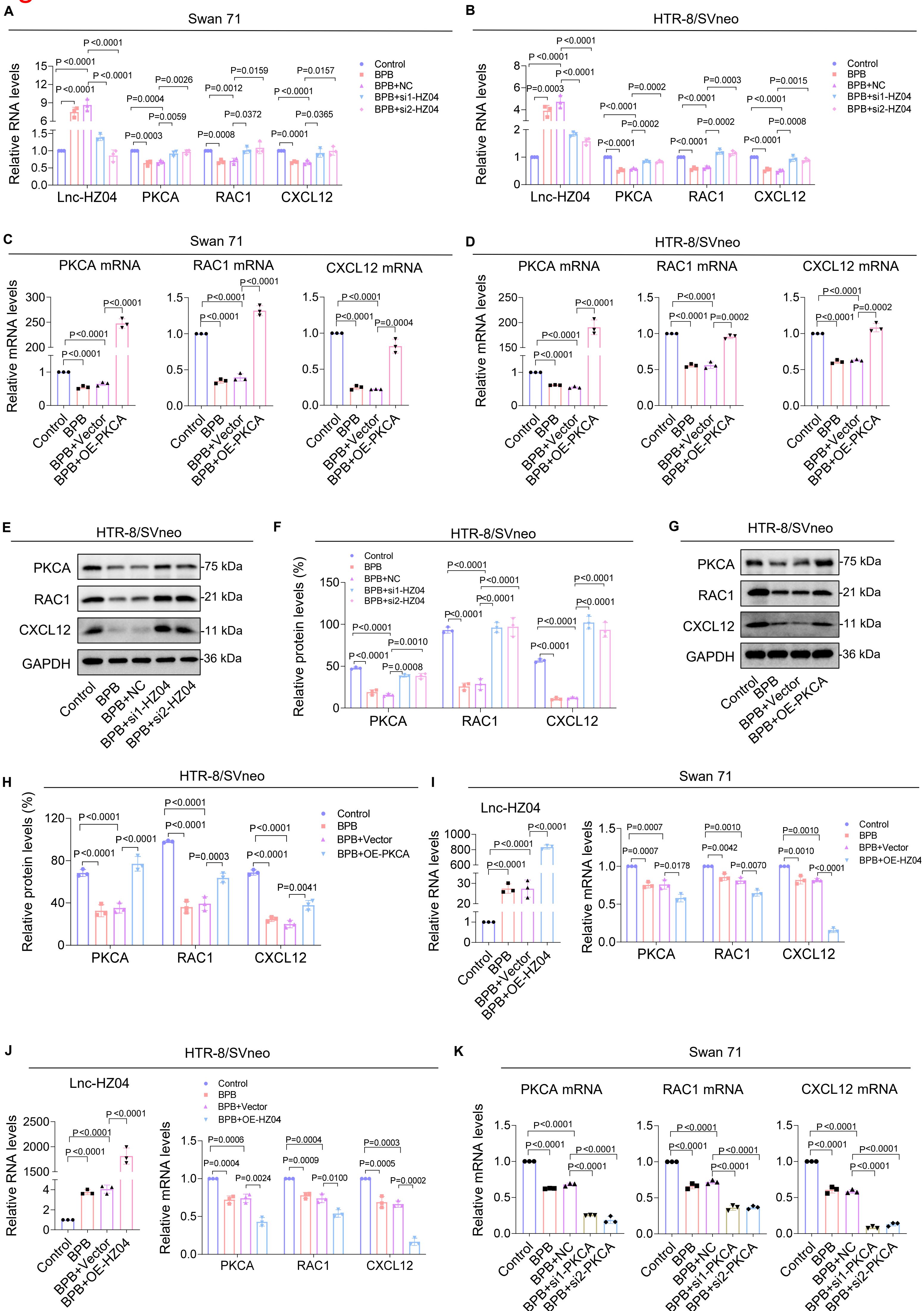

A
Swan 71
Relative RNA levels
Lnc-HZ04
PKCA
RAC1
CXCL12
Control
BPB
BPB+NC
BPB+si1-HZ04
BPB+si2-HZ04
P <0.0001
P=0.0004
P=0.0026
P=0.0059
P=0.0003
P=0.0012
P=0.0159
P=0.0372
P=0.0008
P=0.0157
P=0.0365
B
HTR-8/SVneo
Relative RNA levels
P=0.0003
P<0.0001
P=0.0002
P=0.0015
P=0.0008
C
Swan 71
PKCA mRNA
RAC1 mRNA
CXCL12 mRNA
Relative mRNA levels
Control
BPB
BPB+Vector
BPB+OE-PKCA
P<0.0001
P=0.0004
D
HTR-8/SVneo
PKCA mRNA
RAC1 mRNA
CXCL12 mRNA
Relative mRNA levels
P=0.0002
E
HTR-8/SVneo
PKCA
RAC1
CXCL12
GAPDH
75 kDa
21 kDa
11 kDa
36 kDa
Control
BPB
BPB+NC
BPB+si1-HZ04
BPB+si2-HZ04
F
HTR-8/SVneo
Relative protein levels (%)
P=0.0010
P=0.0008
G
HTR-8/SVneo
BPB+Vector
BPB+OE-PKCA
H
HTR-8/SVneo
Relative protein levels (%)
P=0.0003
P=0.0041
I
Swan 71
Lnc-HZ04
Relative RNA levels
Relative mRNA levels
BPB+OE-HZ04
P=0.0007
P=0.0178
P=0.0010
P=0.0042
P=0.0070
J
HTR-8/SVneo
Lnc-HZ04
P=0.0006
P=0.0004
P=0.0024
P=0.0009
P=0.0100
P=0.0003
P=0.0005
P=0.0002
K
Swan 71
PKCA mRNA
RAC1 mRNA
CXCL12 mRNA
BPB+si1-PKCA
BPB+si2-PKCA

Fig. S8-2

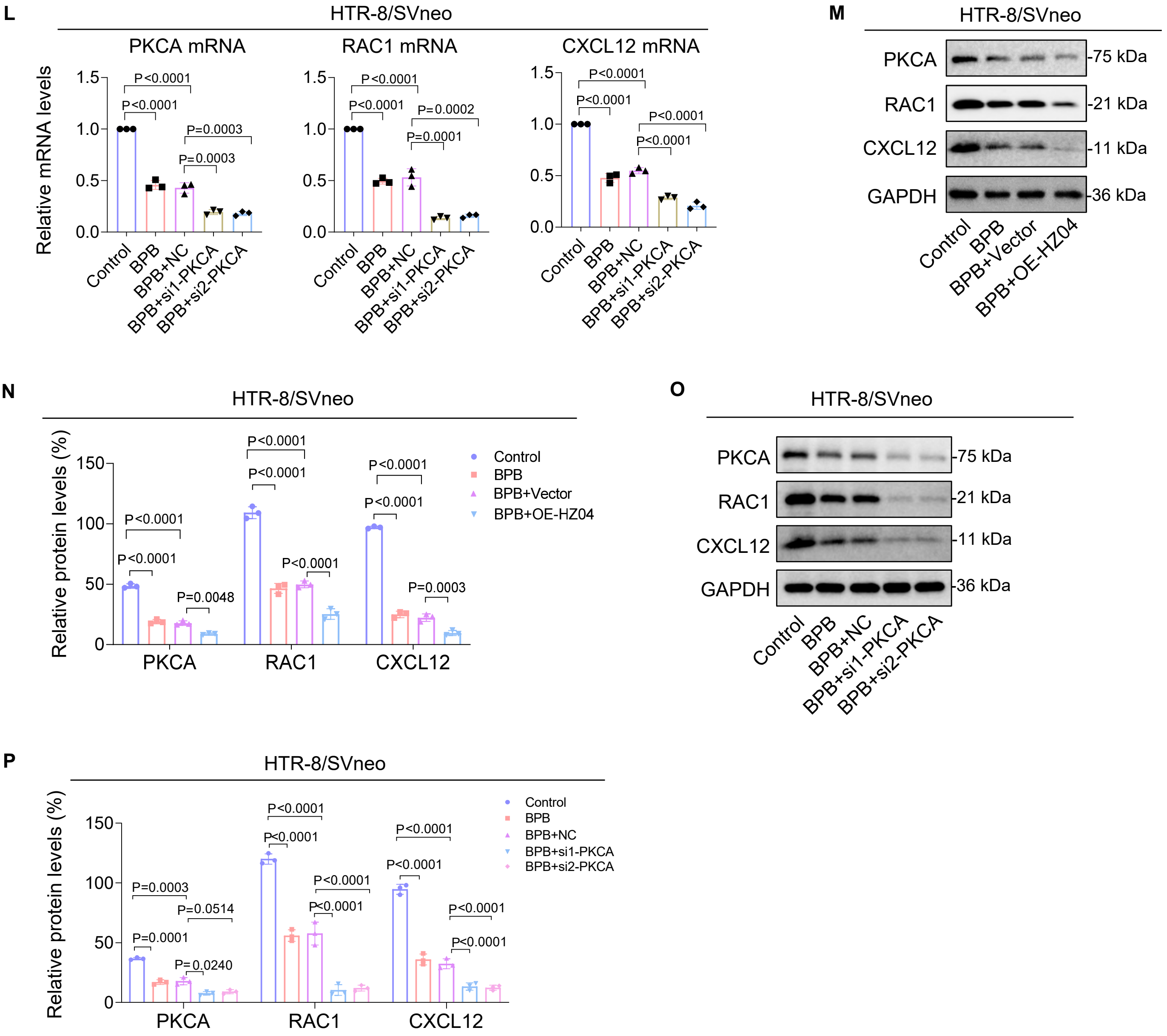
L
HTR-8/SVneo
PKCA mRNA
RAC1 mRNA
CXCL12 mRNA
Relative mRNA levels
1.5
1.0
0.5
0.0
P<0.0001
P=0.0003
P=0.0002
P=0.0001
Control
BPB
BPB+NC
BPB+si1-PKCA
BPB+si2-PKCA
M
HTR-8/SVneo
PKCA
RAC1
CXCL12
GAPDH
75 kDa
21 kDa
11 kDa
36 kDa
Control
BPB
BPB+Vector
BPB+OE-HZ04
N
HTR-8/SVneo
Relative protein levels (%)
150
100
50
0
P<0.0001
P=0.0048
P=0.0003
Control
BPB
BPB+Vector
BPB+OE-HZ04
PKCA
RAC1
CXCL12
O
HTR-8/SVneo
PKCA
RAC1
CXCL12
GAPDH
75 kDa
21 kDa
11 kDa
36 kDa
Control
BPB
BPB+NC
BPB+si1-PKCA
BPB+si2-PKCA
P
HTR-8/SVneo
Relative protein levels (%)
150
100
50
0
P=0.0003
P=0.0514
P=0.0001
P=0.0240
P<0.0001
Control
BPB
BPB+NC
BPB+si1-PKCA
BPB+si2-PKCA
PKCA
RAC1
CXCL12

Fig. S9

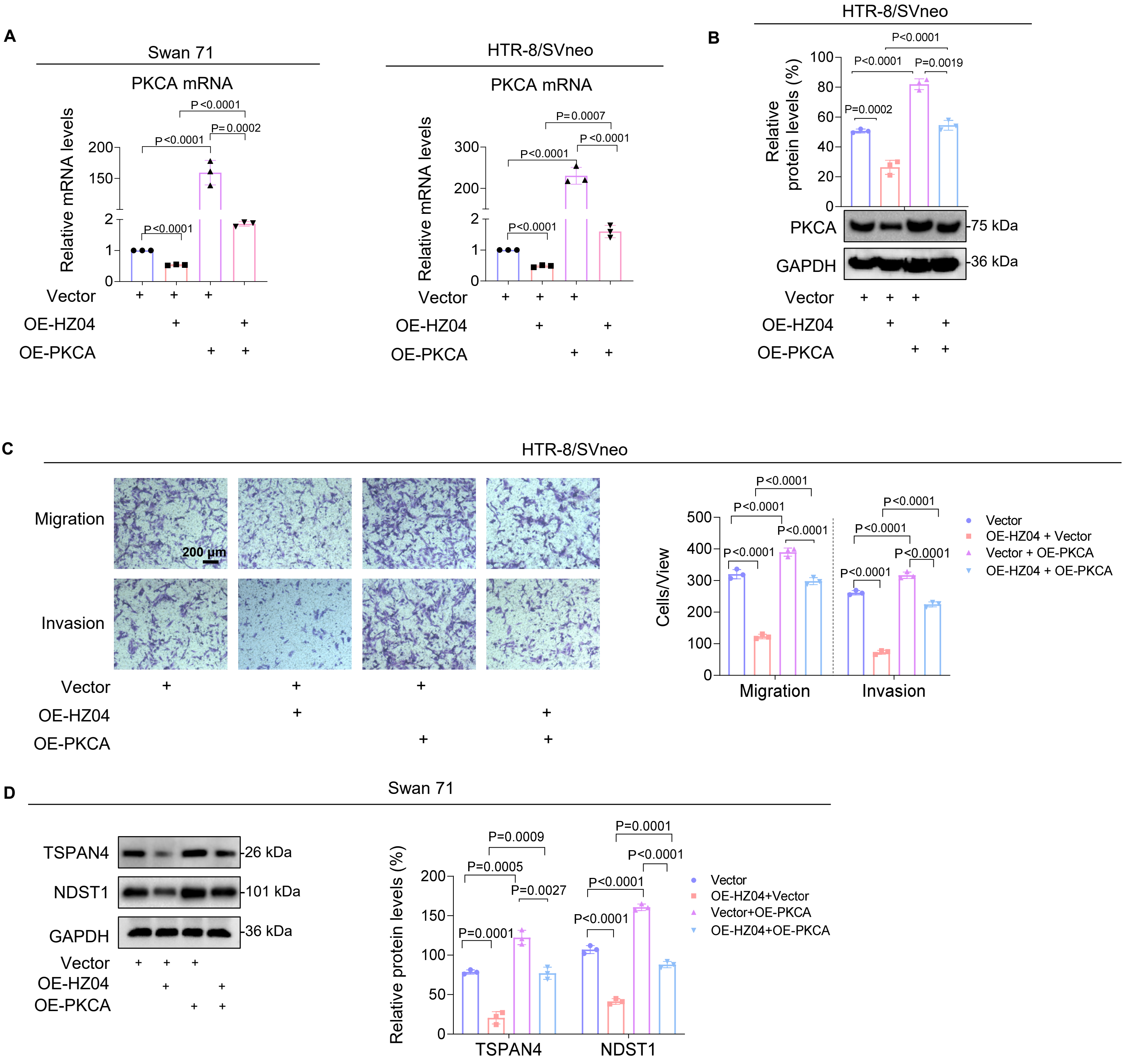

A
Swan 71
PKCA mRNA
Relative mRNA levels
P<0.0001
P=0.0002
P<0.0001
P<0.0001
HTR-8/SVneo
PKCA mRNA
P=0.0007
P<0.0001
P<0.0001
P<0.0001
Vector
OE-HZ04
OE-PKCA
B
HTR-8/SVneo
Relative protein levels (%)
P<0.0001
P<0.0001
P=0.0019
P=0.0002
PKCA
GAPDH
75 kDa
36 kDa
C
HTR-8/SVneo
Migration
Invasion
200 μm
Cells/View
P<0.0001
Vector
OE-HZ04 + Vector
Vector + OE-PKCA
OE-HZ04 + OE-PKCA
D
Swan 71
TSPAN4
NDST1
GAPDH
26 kDa
101 kDa
36 kDa
Relative protein levels (%)
P=0.0009
P=0.0005
P=0.0027
P=0.0001
P<0.0001
P<0.0001
P=0.0001
P<0.0001
OE-HZ04+Vector
Vector+OE-PKCA
OE-HZ04+OE-PKCA

Fig. S10

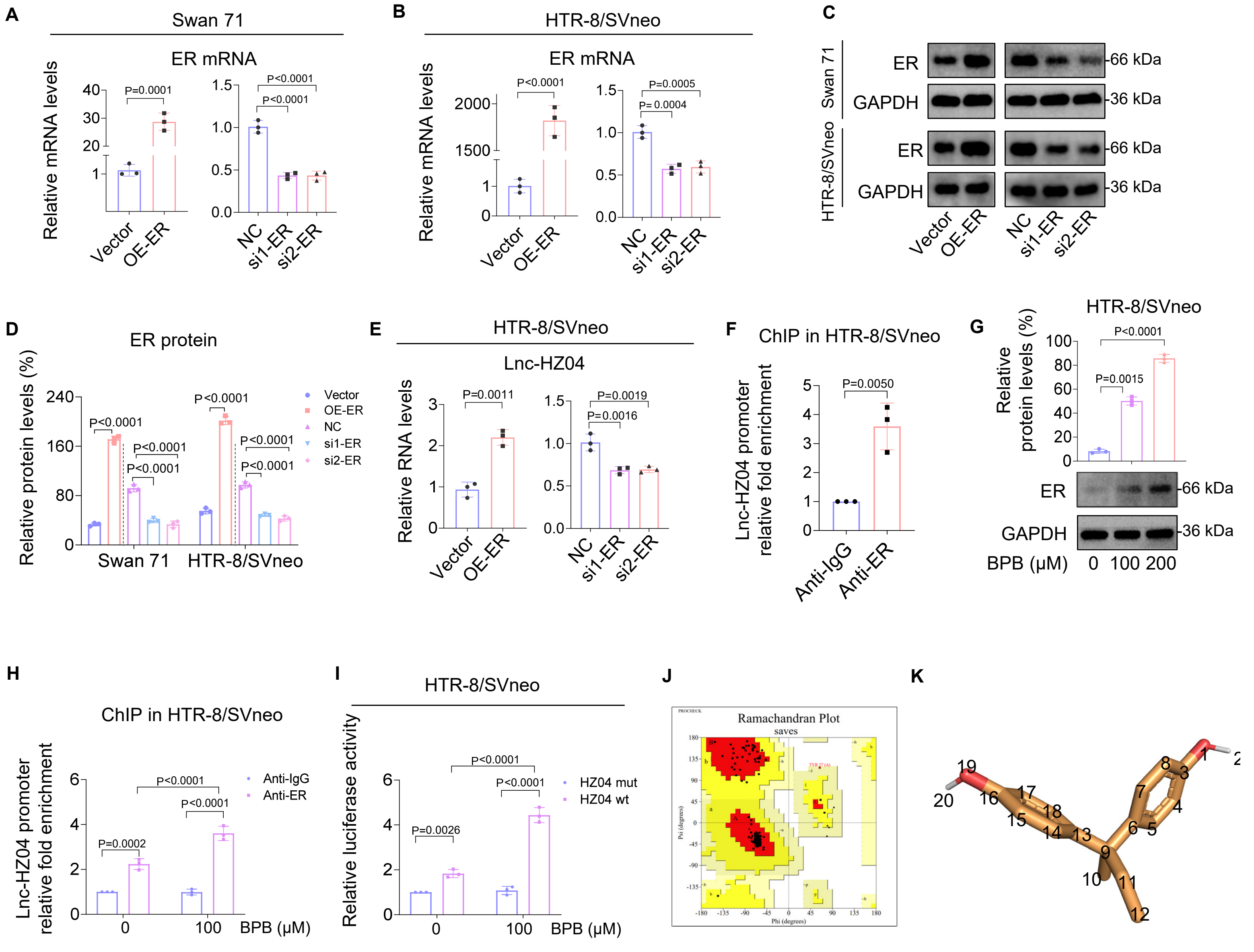
A
Swan 71
ER mRNA
Relative mRNA levels
P=0.0001
P<0.0001
P<0.0001
Vector
OE-ER
NC
si1-ER
si2-ER
B
HTR-8/SVneo
ER mRNA
Relative mRNA levels
P<0.0001
P=0.0005
P=0.0004
Vector
OE-ER
NC
si1-ER
si2-ER
C
Swan 71
HTR-8/SVneo
ER
GAPDH
66 kDa
36 kDa
Vector
OE-ER
NC
si1-ER
si2-ER
D
ER protein
Relative protein levels (%)
P<0.0001
Vector
OE-ER
NC
si1-ER
si2-ER
Swan 71
HTR-8/SVneo
E
HTR-8/SVneo
Lnc-HZ04
Relative RNA levels
P=0.0011
P=0.0019
P=0.0016
Vector
OE-ER
NC
si1-ER
si2-ER
F
ChIP in HTR-8/SVneo
Lnc-HZ04 promoter
relative fold enrichment
P=0.0050
Anti-IgG
Anti-ER
G
HTR-8/SVneo
Relative
protein levels (%)
P<0.0001
P=0.0015
ER
GAPDH
66 kDa
36 kDa
BPB (μM) 0 100 200
H
ChIP in HTR-8/SVneo
Lnc-HZ04 promoter
relative fold enrichment
P<0.0001
P<0.0001
P=0.0002
Anti-IgG
Anti-ER
0
100
BPB (μM)
I
HTR-8/SVneo
Relative luciferase activity
P<0.0001
P<0.0001
P=0.0026
HZ04 mut
HZ04 wt
0
100
BPB (μM)
J
Ramachandran Plot
saves
K

Fig. S11

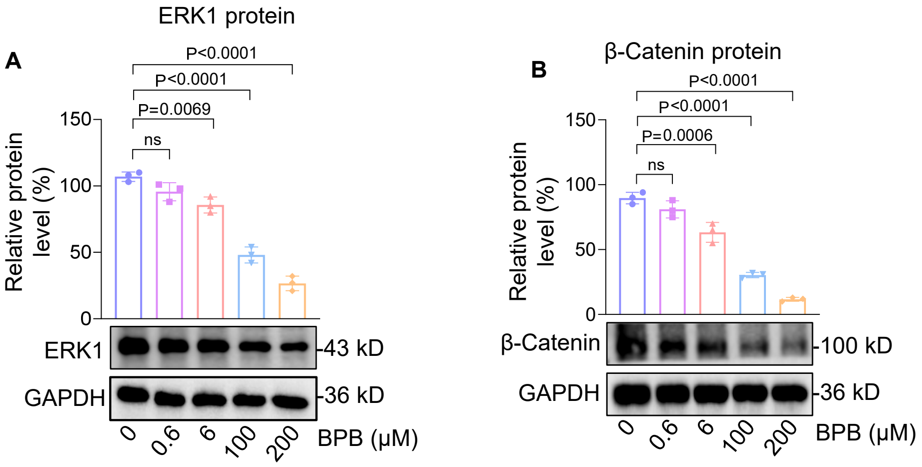

A
ERK1 protein
P<0.0001
P<0.0001
P=0.0069
ns
Relative protein level (%)
150
100
50
0
ERK1
-43 kD
GAPDH
-36 kD
0
0.6
6
100
200
BPB (μM)
B
β-Catenin protein
P<0.0001
P<0.0001
P=0.0006
ns
Relative protein level (%)
150
100
50
0
β-Catenin
-100 kD
GAPDH
-36 kD
0
0.6
6
100
200
BPB (μM)
