# Supplementary material for: Bisphenol B Exposure Induces Miscarriage by Suppressing Migration/Invasion and Migrasome Formation
Source: Adv Sci (Weinh). 2025 Nov 21;13(7):e04871. doi: 10.1002/advs.202504871 (PMC12866717; doi:10.1002/advs.202504871)
Supplement: Supplementary file 2 — Supporting Information [file ADVS-13-e04871-s002.docx]

**Supplementary Table**

Bisphenol B exposure induces miscarriage by suppressing migration/invasion and migrasome formation

Wenxin Huang1,#, Manli Wang1,#, Yi Sun1,#, Xiaoping Yue3, Xun Li4, Yanbing Lin1, Geng Guo5, Depeng Zhao6, Qigang Fan1, Xiaoling Ma7, Zhihong Zhang8, Shuaishuai Xing1, Xiaoting Shen2,*, and Huidong Zhang1,*

1Research Center for Environment and Female Reproductive Health, the Eighth Affiliated Hospital, Sun Yat-sen University, Shenzhen 518033, China
2NHC Key Laboratory of Male Reproduction and Genetics, Guangdong Provincial Reproductive Science Institute (Guangdong Provincial Fertility Hospital), Guangzhou 510600, China.
3Key Laboratory of Pharmaceutical Quality Control of Hebei Province, College of Pharmaceutical Sciences, Hebei University, Baoding 071002, China
4Department of General Surgery, The First Hospital of Lanzhou University, Lanzhou 730000, China
5Department of Emergency; Cerebrovascular disease center, First Hospital of Shanxi Medical University, Taiyuan, 030001, China
6Department of Reproductive Medicine, Shenzhen Maternity and Child Healthcare Hospital, Women and Children's Medical Center, Southern Medical University, Shenzhen, 518033, Guangdong Province, China
7Department of Reproductive Medicine, The First Hospital of Lanzhou University, the First Clinical Medical School of Lanzhou University, Lanzhou 730000, China
8MOE Key Laboratory of Coal Environmental Pathogenicity and Prevention, Shanxi Medical University, Taiyuan, 030001, China.

#These authors contributed equally to this work.
*Corresponding author. E-mail: shenxt@gdszjk.org.cn (X. S.) and zhanghd29@mail.sysu.edu.cn (H. Z.)

Xiaoting Shen, ORCID ID: 0000-0001-9684-7852
Huidong Zhang, ORCID ID: 0000-0001-7845-3331

**Table S1. The characteristic of UM and HC women.**

| **Characteristic** | **UM*^e^* (n = 100)** | **HC*^f^* (n = 100)** | **P-value** |
| --- | --- | --- | --- |
| **Age (years)** | 27.9 ± 1.41*^g^* | 27.73 ± 1.48 | 0.558***^i^*** |
| **Gestational weeks** | 7.06 ± 0.84 | 7.03 ± 0.80 | 0.796 |
| **Gravidity** | 1.80 ± 0.943 | 1.63 ± 0.80 | 0.171 |
| **BMI*^a^*** | 21.7 ± 2.31 | 21.8 ± 2.07 | 0.755 |
| **RBC*^b^* (10^12^/L)** | 4.71 ± 0.25 | 4.68 ± 0.27 | 0.416 |
| **WBC*^c^* (10^9^/L)** | 6.82 ± 1.61 | 6.75 ± 1.59 | 0.757 |
| **Hb*^d^* (g/L)** | 139 ± 8.52 | 141 ± 8.43 | 0.097 |
| **Residence** |  |  | 0.320 ***^j^*** |
| Rural | 42*^h^* | 49 |  |
| Urban | 58 | 51 |  |
| **Education** |  |  | 0.648 |
| < High school | 30 | 33 |  |
| ≥ High school | 70 | 67 |  |
| **Taking canned food and bottled drinks in the past 3 months** | | | 0.253 |
| 0-4 times per week | 39 | 47 |  |
| ≥ 4 times per week | 61 | 53 |  |
| **Household income, RMB Yuan/month** | | | 0.457 |
| < 5000 | 37 | 32 |  |
| ≥ 5000 | 63 | 68 |  |
| **Smoking in the past 3 months** | |  | 0.645 |
| No | 68 | 71 |  |
| Yes | 32 | 29 |  |
| **Drinking in the past 3 months** | |  | 0.345 |
| No | 75 | 69 |  |
| Yes | 25 | 31 |  |

*^a^*BMI: Body Mass Index.

*^b^*RBC: Red blood cell.

*^c^*WBC: White blood cell.

*^d^*Hb: Hemoglobin.

*^e^*UM: unexplained miscarriage group.

*^f^*HC: healthy control group.

*^g^*mean ± standard deviation (n = 100 in each UM or HC group).

*^h^*number of women (n = 100 in each UM or HC group).

*^i^*Student's t-test.

*^j^*Chi-square test.

**Table S2. Multivariate logistic regression analysis of the characteristic of UM and HC women***^a^***.**

| **Characteristic** | **P-value** | **OR***^b^* | **95% CI** |
| --- | --- | --- | --- |
| Unadjusted BPB levels in urine | 0.035 | 1.483 | 1.027 - 2.141 |
| Adjusted BPB levels in urine | 0.034 | 1.507 | 1.030 - 2.205 |
| Unadjusted PKCA mRNA levels in villous tissues | < 0.001 | 0.389 | 0.230 - 0.656 |
| Adjusted PKCA mRNA levels in villous tissues | 0.001 | 0.286 | 0.141 - 0.580 |
| Unadjusted lnc-HZ04 levels in villous tissues | < 0.001 | 3.278 | 1.889 - 5.689 |
| Adjusted lnc-HZ04 levels in villous tissues | < 0.001 | 4.469 | 2.174 - 9.188 |
| Unadjusted lnc-HZ04 levels in serum | < 0.001 | 1.250 | 1.105 - 1.415 |
| Adjusted lnc-HZ04 levels in serum | 0.001 | 1.300 | 1.108 - 1.525 |

*^a^*Adjusted for age, BMI, education, household income, smoking, drinking, taking canned food and bottled drinks.

*^b^*Adjusted OR.

**Table S3. Sequence conservation analysis of the mRNA and protein in human with those in other various species*^a^***

|  |  |  | **Rhesus** | **Mouse** | **Dog** | **Elephant** |
| --- | --- | --- | --- | --- | --- | --- |
| **mRNA** | PKCA | Per.Ident*^b^* | 94.32% | 83.55% | 92.73% | 89.67% |
|  | RAC1 | Per.Ident | 77.52% | 95.41% | 87.60% | 93.96% |
|  | CXCL12 | Per.Ident | 98.50% | 89.46% | 89.62% | 88.22% |
|  | TSPAN4 | Per.Ident | 94.60% | 86.6% | 85.98% | 88.55% |
|  | NDST1 | Per.Ident | 97.95% | 88.83% | 92.71% | 91.28% |
| **Protein** | PKCA | Per.Ident | 99.70% | 98.21% | 99.11% | 98.96% |
|  | RAC1 | Per.Ident | 100% | 100% | 100% | 99.32% |
|  | CXCL12 | Per.Ident | 94.24% | 92.50% | 93.30% | 84.95% |
|  | TSPAN4 | Per.Ident | 99.16% | 100% | 83.61% | 92.44% |
|  | NDST1 | Per.Ident | 99.38% | 97.73% | 98.75% | 97.73% |

*^a^*Sequence conservation of PKCA, RAC1, CXCL12, TSPAN4, and NDST1 was explored using NCBI Blast (<https://blast.ncbi.nlm.nih.gov/Blast.cgi>).

*^b^*Per.Ident means the ratio of the number of the matched bases to that of the total bases.

**Table S4. Hydrophobic interactions between BPB and ER protein.**

| **Amino acid residues** | **BPB atom listed in Fig. S10L** | **Distance (**Å**)** |
| --- | --- | --- |
| Leu-42 | 14 | 3.45 |
| Leu-42 | 11 | 3.61 |
| Thr-43 | 15 | 3.77 |
| Ala-46 | 17 | 3.56 |
| Trp-79 | 17 | 4.00 |
| Leu-80 | 18 | 3.59 |
| Leu-83 | 4 | 3.77 |
| Leu-87 | 4 | 3.44 |
| Phe-100 | 11 | 3.60 |
| Ile-120 | 12 | 3.92 |
| Phe-121 | 12 | 3.92 |
| Leu-221 | 17 | 3.74 |

**Table S5. H-bonds between BPB and ER.**

| **Amino acid residues** | **DistanceH-A***^a^* | **DistanceD-A***^b^* |
| --- | --- | --- |
| Glu-49 | 2.20 | 2.90 |
| Arg-90 | 2.55 | 3.48 |

*^a^*Distance H-A: The distance between hydrogen atom and the atom that accepts hydrogen atom.

*^b^*Distance D-A: The distance between the two atoms of the entire H-bond.

**Table S6. Pi-Stacking interactions between BPB and ER.**

| **Amino acid residues** | **Distance (Å)** | **Angle (°)** | **Stacking Type** | **BPB atoms listed in Fig. S10L** |
| --- | --- | --- | --- | --- |
| Phe-100 | 4.95 | 84.84 | T-Stacking | 3, 4,5, 6, 7,8 |

**Table S7. The dsDNA promoter region of lnc-HZ04 recognized by ER.**

| **Lnc-HZ04 promoter region** |
| --- |
| **5'-**GGCATGAGCCACCCTGCCCA**-3'/** **3’-CCGTACTCGGTGGGACGGGT-5’** |

**Table S8. Hydrogen bonding interactions between amino acid residues in ER and bases in dsDNA (promoter region of lnc-HZ04)**

| **Amino acid residues** | **Bases***^a^* | **H-bond distance (Å)** |
| --- | --- | --- |
| His-172 | DG-2 | 2.3 |
| His-172 | DC-3 | 2.9 |
| Asn-151 | DG-12 | 3.4 |
| Tyr-155 | DG-11 | 3.6 |
| Tyr-155 | DG-11 | 2.5 |

*^a^*Bases were listed in Fig. 8L.

**Table S9. The binding energy of partial key residues in ER with bases in dsDNA.**

| **Key residue** | **Total Energy (kcal/mol)** | | **Δ Energy***^a^* **(kcal/mol)** |
| --- | --- | --- | --- |
|  | **Complex-ER-dsDNA** | **Complex-ER-dsDNA-BPB** |  |
| Arg-108 | -1.59 | -0.28 | 1.31 |
| Lys-112 | -0.58 | -0.15 | 0.43 |
| Ala-126 | -0.26 | -0.28 | -0.02 |
| Arg-130 | -2.73 | -3.92 | -1.18 |
| Arg-132 | -1.24 | -0.30 | 0.94 |
| Met-133 | -2.96 | -0.50 | 2.46 |
| Met-134 | -0.79 | -0.26 | 0.53 |
| Leu-193 | -1.12 | -0.15 | 0.97 |
| Gln-194 | -2.09 | -0.72 | 1.37 |
| Gln-195 | -1.04 | -0.17 | 0.87 |
| His-197 | -0.10 | -4.69 | -4.59 |
| Gln-198 | -0.71 | -3.45 | -2.74 |
| Arg-199 | -0.77 | -0.63 | 0.14 |
| Gln-202 | -0.91 | -2.43 | -1.52 |
| Leu-205 | -0.14 | -5.21 | -5.07 |
| Arg-211 | -0.10 | -0.67 | -0.57 |
| Lys-216 | -0.22 | -0.39 | -0.17 |

*^a^*Energy: The changes in binding energy of each residue with DNA after binding with BPB.

**Table S10. DNA sequences used for construction of pcDNA3.1 overexpression plasmids.**

| **Species** | **Plasmid name** | **Gene name** | **Sequence region** |
| --- | --- | --- | --- |
| Human | pcDNA3.1- HZ04 | Lnc-HZ04 | Full sequence, NCBI No. MT821846 |
|  | pcDNA3.1-PKCA | PKCA | CDS region (NM_002737.3) |
|  | pcDNA3.1-TCF4 | TCF4 | CDS region (NM_001243234.2) |
|  | pcDNA3.1-ER | ER | CDS region (NM_000125.4) |
| Mouse | pcDNA3.1-Pkca | Pkca | CDS region (NM_011101.3) |
|  | pcDNA3.1-Tspan4 | Tspan4 | CDS region (NM_001252588.2) |

**Table S11. RNA sequences used for RNA transfection.**

| **Name** | **Sense (5'-3')** | **Antisense (5'-3')** |
| --- | --- | --- |
| si1-HZ04 | CCUACCUUCUUCGGUUUCUTT | AGAAACCGAAGAAGGUAGGTT |
| si2-HZ04 | GCCGUGAUCAGCUGUUGUUTT | AACAACAGCUGAUCACGGCTT |
| si1-PKCA | CCCGUCUUAACACCACCUGAUTT | AUCAGGUGGUGUUAAGACGGGTT |
| si2-PKCA | AUCAGGUGGUGUUAAGACGGGTT | UAUUCCAUGACGAAGUACAGCTT |
| si1-TCF4 | CUAUCAGUAUUCUAGCAAUAATT | UUAUUGCUAGAAUACUGAUAGTT |
| si2-TCF4 | CGAAUUGAAGAUCGUUUAGAATT | UUCUAAACGAUCUUCAAUUCGTT |
| si1-ER | GCCCUACUACCUGGAGAACGATT | UCGUUCUCCAGGUAGUAGGGCTT |
| si2-ER | CUCUACUUCAUCGCAUUCCUUTT | AAGGAAUGCGAUGAAGUAGAGTT |
| NC | UUCUCCGAACGUGUCACGUTT | ACGUGACACGUUCGGAGAATT |

**Table S12. Primer sequences used for RT-qPCR.**

| **Species** | **Gene** | **Forward (5'-3')** | **Reverse (5'-3')** |
| --- | --- | --- | --- |
| Human | Lnc-HZ04 | TAAATCCAGCGCGCAAGAAC | CCGAAGAAGGTAGGAGAGGGA |
|  | PKCA | GTCCACAAGAGGTGCCATGAA | AAGGTGGGGCTTCCGTAAGT |
|  | RAC1 | ATGTCCGTGCAAAGTGGTATC | CTCGGATCGCTTCGTCAAACA |
|  | CXCL12 | ATTCTCAACACTCCAAACTGTGC | ACTTTAGCTTCGGGTCAATGC |
|  | TCF4 | GCCTGGAGTCAGGAATGCTT | TCAAACCGCCTGTCACTCTC |
|  | ER | GGGAAGTATGGCTATGGAATCTG | TGGCTGGACACATATAGTCGTT |
|  | GAPDH | TGTGTCCGTCGTGGATCTGA | GCAGCTGTGACACACAGTA |
|  | PKCA promoter | AGCGAGTGATTAGCTGGTGC | GAGTGCAGCCAGTACGTTTG |
|  | Lnc-HZ04 promoter | TAGGTTGGTCTCATTCCCGAC | GAATCCGGAGGTGGTGGTT |
| Mouse | Pkca | GTTTACCCGGCCAACGACT | TCTTTCACCTCATGCACGTTC |
|  | Rac1 | GTCCGTGCAAAGTGGTATCCT | GCACCGATCTCTTTCGCCAT |
|  | Cxcl12 | TGCATCAGTGACGGTAAACCA | CACAGTTTGGAGTGTTGAGGAT |
|  | Tspan4 | TACCTCATGTTCGCCTTCAAC | GATAAGGTGGCAAAGTTTCCCT |
|  | Ndst1 | CGGAGAGGATTGGACTGTGTT | GTGTGGGATGGATTCGGAGG |
|  | Gapdh | TGGCCTTCCGTGTTCCTAC | GAGTTGCTGTTGAAGTCGCA |

**Table S13. The wild-type or mutant promoter sequence of PKCA and lnc-HZ04**

| **Genes** | **Wild-type (5’-3’)** | **Mutant (5’-3’)** |
| --- | --- | --- |
| PKCA | CCCACCTGCG | GGGTGGACGC |
| Lnc-HZ04 | GGCATGAGCCACCCTGCCCA | CCGTACTCGGTGGGACGGGT |
