# Supplementary material for: Bisphenol B Exposure Induces Miscarriage by Suppressing Migration/Invasion and Migrasome Formation
Source: Adv Sci (Weinh). 2025 Nov 21;13(7):e04871. doi: 10.1002/advs.202504871 (PMC12866717; doi:10.1002/advs.202504871)
Supplement: Supplementary file 3 — Supporting Information [file ADVS-13-e04871-s003.docx]

Survey Form Number: □□-□□-□□-□□-□□

Name: Case ID:

Pre-Diagnosis Survey Form

Dear patient,

To ensure the quality of your medical treatment, to timely identify causes, make more accurate etiological diagnoses, and thus provide targeted treatment and prognosis rehabilitation, Sun Yat-sen University Eighth Affiliated Hospital has launched a personalized treatment plan. We understand the impact of your life, behavior, and environmental factors on your health status through personalized questionnaires, and provide you with a free health status file. With the file number, you will have a VIP card with priority medical treatment in this outpatient department. We will then conduct long-term follow-up surveys, closely monitor your health status, and provide you with personalized prevention and health care advice as well as individualized treatment. This survey will only take about twenty minutes, and the information obtained in the survey will be completely confidential.

All information in this questionnaire is a confidential document.

Only research staff have the right to read it.

Researchers who wish to use this questionnaire

Please contact: Teacher Huang huangwx76@mail2.sysu.edu.cn

Sun Yat-sen University Eighth Affiliated Hospital

Date: .

I. General Information

1. Name: , Date of Birth: , Ethnicity: , Height: , Weight: , Contact Phone: Email: .

2. Your level of education:

① Primary school ② Junior high school ③ High school or technical secondary school

④ College or undergraduate ⑤ Master's or above ⑥ Other

3. Your occupation is:

① Service industry personnel ② Science/Medical/Teacher ③ Hairdresser ④ Farmer

⑤ Worker ⑥ Clerk ⑦ Housewife ⑧ Courier ⑨ Individual business owner

⑩ Other, please specify:

4. Your place of residence?

① Rural ② Urban

5. Your personal monthly income?

① <2000 ② 2000～3000 ③ 3000～5000 ④ 5000～10000

⑤ 10000～20000 ⑥ More than 20000

6. Have you had a premarital medical examination before marriage?

① Yes (If the premarital medical examination is abnormal, please state the diagnosis ) ② No ③ Unmarried

7. Do you have medical insurance?

① Yes ② No

7. Age at menarche: years old;

Your age at first marriage: years old

8. How often do you menstruate?

① 1 week ② 2 weeks ③ 3-5 weeks ④ >5 weeks ⑤ Irregular

9. How many days does your menstruation last each time?

① 1 day ② 2 days ③ 3-8 days ④ 9 days or more

10. How is your menstrual flow?

① Scant ② Normal ③ Moderate ④ Heavy

11. Does your dysmenorrhea severely affect your life and work?

① Yes ② No

II. Pregnancy Conditions

1. Up to now, how many children have you given birth to? Number of children is .

2. Have you experienced the following during your pregnancy and childbirth?

① Premature birth ② Full-term birth ③ Post-term birth ④ Spontaneous abortion

⑤ Stillbirth ⑥ Never given birth

3. Have you ever had a miscarriage before?

① Yes, times miscarriage ② No

4. If you have had a miscarriage, what type of miscarriage was it diagnosed as?

① Threatened abortion ② Inevitable abortion ③ Incomplete abortion

④ Complete abortion ⑤ Missed abortion ⑥ Induced abortion ⑦ Abortion with infection ⑧ Habitual abortion

5. Have your offspring had the following conditions?

① Low birth weight (< 2500 g/less than 5 pounds) ② Congenital deformities ③ Neonatal encephalopathy ④ Neonatal death ⑤ Other ⑥ None of the above

6. Was your current pregnancy unplanned or planned?

① Unplanned pregnancy ② Planned pregnancy

7. If it was an unplanned pregnancy, did you take contraceptives in the 6 months before getting pregnant?

① Yes ② No

8. In the 6 months before pregnancy, did you take any ovulation-inducing drugs?

① Clomiphene citrate ② Estrogen and progesterone ③ Human chorionic gonadotropin ④ Luteinizing hormone-releasing hormone ⑤ Bromocriptine ⑥ Traditional Chinese herbal medicine 'Duozai Pill' (known for its claimed effect on increasing the chance of multiple births) ⑦ Other ⑧ Not taken

9. If you have taken ovulation drugs, how long did you take them for? days

10. Before this pregnancy, did you have any of the following diseases? (Multiple choices are available)

① Hypertension ② Diabetes ③ Heart disease ④ Preeclampsia ⑤ Abortion ⑥ Intrauterine growth restriction ⑦ Autoimmune diseases (such as: systemic lupus erythematosus, rheumatoid arthritis, scleroderma, polyarteritis nodosa) ⑧ Endometriosis ⑨ Uterine fibroids ⑩ Other diseases.

11. If you have any of the above diseases, what is the name of the medication? Started taking it in the month before pregnancy; Duration of medication days

12. Do you often take anti-inflammatory drugs (antibiotics)?

① Yes ② No

13. Have you taken any of the following anti-inflammatory drugs in the past six months? Check the categories you have taken.

① Penicillins: Penicillin G, Cloxacillin, Dicloxacillin, Bicloxacillin, Ampicillin, Amoxicillin;

② Cephalosporins: Cefazolin, Cefalexin, Cefuroxime, Cefprozil, Ceftriaxone, Ceftazidime, Cefoperazone, etc.

③ Aminoglycosides: Streptomycin, Gentamicin, Tobramycin, Netilmicin, Spectinomycin;

④ Macrolides: Erythromycin, Roxithromycin, Azithromycin, Clarithromycin;

⑤ Quinolones: Ofloxacin, Ciprofloxacin, Moxifloxacin, Gatifloxacin, Pazufloxacin;

⑥ Carbapenems and Oxapenems: Imipenem, Meropenem, Panipenem, Ertapenem;

14. How long have you been taking the above-mentioned medications?

① Less than 3 days ② 3-7 days ③ 7-14 days ④ More than 14 days

15. Did you have any of the following during pregnancy?

① Gestational diabetes ② Gestational hypertension ③ Pregnancy with heart disease ④ Preeclampsia ⑤ Fetal growth restriction ⑥ Fetal deformity ⑦ Macrosomia ⑧ Antinuclear antibody positivity (with titer as ) ⑨ None

16. Have you taken any medication to maintain pregnancy before?

① Yes ② No

17. How often did you take folic acid in the 3 months before pregnancy?

① Rarely ② 1-4 times a week ③ 4 or more times a week

18. How much folic acid did you take in the 3 months before pregnancy?

① Rarely ② 0.4 mg per day ③ More than 0.4 mg per day

19. How often did you take multivitamins in the 3 months before pregnancy?

① Rarely ② 1-4 times a week ③ 4 or more times a week

20. How often did you take iron and calcium in the 3 months before pregnancy?

① Rarely ② 1-4 times a week ③ 4 or more times a week

III. Smoking and Drinking

1. Do you smoke (referring to at least one cigarette a day, for half a year or more)?

① Non-smoker ② Less than 2 years ③ More than 2 years

2. In the past six months, how many cigarettes do you usually smoke per day?

① Less than 5 ② 5-10 ③ 10-20 ④ 20-30 ⑤ More than 30

3. In the past six months, have you been exposed to secondhand smoke for more than 15 minutes every day?

① No ② Less than 1 day/week ③ 1～3 days/week ④ 3 days or more/week ⑤ Almost every day

4. Do you have a habit of drinking alcohol?

① Never or almost never drink ② Occasionally drink, 1-4 times a week ③ Often drink, more than 4 times a week

5. On average, how much alcohol do you drink daily or weekly?

Baijiu (liang) (per day or week), Beer (bottle) (per day or week),

Red wine (glass) (per day or week)

III. Lifestyle

1. What type of water do you usually drink?

① Tap water ② Barreled purified water ③ Barreled mineral water ④ Alkaline water

2. How often do you use disposable tableware for food/drinks/water?

① Daily ② Often (more than 7 times/week) ③ Usually (5-7 times/week) ④ Occasionally (1-4 times/week) ⑤ Almost never

3. How often do you order takeout in a week?

① Daily ② Often (more than 7 times/week) ③ Usually (5-7 times/week) ④ Occasionally (1-4 times/week) ⑤ Never order takeout

4. Have you dyed your hair in the past three months?

① Yes ② No

III. Medical History and Family History

1. Has your mother ever given birth to premature or malformed children?

① Yes ② No ③ Don't know

2. Has your husband's mother ever given birth to premature or malformed children?

① Yes ② No ③ Don't know

3. Have any of your family members or relatives ever had or currently have the following diseases? (Multiple choices allowed)

① Coronary heart disease ② Cerebral stroke ③ Hypertension ④ Diabetes ⑤ Kidney disease ⑥ Cancer ⑦ Autoimmune diseases (such as: systemic lupus erythematosus, rheumatoid arthritis, scleroderma, polyarteritis nodosa) ⑧ Allergic diseases (rhinitis, asthma) ⑨ Other diseases:

IV. Home Environment

1. Has your home been renovated?

① Yes ② No

2. If yes, how long ago was the renovation?

① ≤6 months ② 6-12 months ③ 1-2 years ④ ≥2 years

3. During the indoor renovation, which of the following methods were used? (Multiple choices allowed)

① Paint ② Wooden floor ③ Floor tiles ④ Carpet ⑤ Wall paint or wallpaper ⑥ Purchase of new furniture ⑦ Whole house customization (materials: particle board, multilayer board, solid wood) ⑧ Other
